# Systematic review and meta-analysis of diagnostic methods for occlusal surface caries

**DOI:** 10.1007/s00784-021-04024-1

**Published:** 2021-06-14

**Authors:** Svetlana Kapor, Mila Janjic Rankovic, Yegane Khazaei, Alexander Crispin, Ina Schüler, Felix Krause, Adrian Lussi, Klaus Neuhaus, Florin Eggmann, Stavroula Michou, Kim Ekstrand, Marie-Charlotte Huysmans, Jan Kühnisch

**Affiliations:** 1 Department of Conservative Dentistry and Periodontology, University Hospital, Ludwig-Maximilian University, Munich, Germany; 2 Department of Orthodontics and Dentofacial Orthopedics, University Hospital, Ludwig-Maximilian Universität München, Munich, Germany; 3 Institute of Medical Biometry and Epidemiology, Ludwig-Maximilian University of Munich, Munich, Germany; 4 Department of Orthodontics, Section of Preventive and Paediatric Dentistry, University Hospital, Jena, Germany; 5 Clinic for Operative Dentistry, Periodontology and Preventive Dentistry, University Hospital RWTH Aachen, Aachen, Germany; 6 Department of Operative Dentistry and Periodontology, Faculty of Dentistry, University Medical Centre, Freiburg, Germany; 7 School of Dental Medicine, University of Bern, Bern, Switzerland; 8 Clinic of Periodontology, Endodontology and Cariology, University Centre for Dental Medicine Basel, University of Basel, Basel, Switzerland; 9 Department of Dermatology, Inselspital-Bern University Hospital, Bern, Switzerland; 10 Department of Odontology, University Copenhagen, Copenhagen, Denmark; 11 Department of Dentistry, Radboud University Medical Centre, Nijmegen, the Netherlands; 12 Poliklinik Für Zahnerhaltung Und Parodontologie, Klinikum Der Universität München, LMU München, Goethestraße 70, 80336 München, Germany

**Keywords:** Occlusal caries, Pit and fissure caries, Caries detection, Caries diagnostics, Visual examination, Bitewing radiography, Laser fluorescence measurements, Fibre-optic transillumination, Systematic review, Meta-analysis, Diagnostic performance, Accuracy, Sensitivity, Specificity

## Abstract

**Aim:**

This systematic review and meta-analysis aimed to assess the diagnostic performance of commonly used methods for occlusal caries diagnostics, such as visual examination (VE), bitewing radiography (BW) and laser fluorescence (LF), in relation to their ability to detect (dentin) caries under clinical and laboratory conditions.

**Materials and methods:**

A systematic search of the literature was performed to identify studies meeting the inclusion criteria using the PIRDS concept (*N* = 1090). A risk of bias (RoB) assessment tool was used for quality evaluation. Reports with low/moderate RoB, well-matching thresholds for index and reference tests and appropriate reporting were included in the meta-analysis (*N* = 37; 29 in vivo/8 in vitro). The pooled sensitivity (SE), specificity (SP), diagnostic odds ratio (DOR) and areas under ROC curves (AUCs) were computed.

**Results:**

SP ranged from 0.50 (fibre-optic transillumination/caries detection level) to 0.97 (conventional BW/dentine detection level) in vitro. AUCs were typically higher for BW or LF than for VE. The highest AUC of 0.89 was observed for VE at the 1/3 dentin caries detection level; SE (0.70) was registered to be higher than SP (0.47) for VE at the caries detection level in vivo.

**Conclusion:**

The number of included studies was found to be low. This underlines the need for high-quality caries diagnostic studies that further provide data in relation to multiple caries thresholds.

**Clinical relevance:**

VE, BW and LF provide acceptable measures for their diagnostic performance on occlusal surfaces, but the results should be interpreted with caution due to the limited data in many categories.

**Supplementary Information:**

The online version contains supplementary material available at 10.1007/s00784-021-04024-1.

## Introduction

Over the last several decades, occlusal surfaces have been found to be one of the most prevalent sites for caries development in children and adolescents, mainly due to their anatomical susceptibility [1–6]. Because a valid and reproducible caries diagnosis and assessment could not be made by visual examination (VE) alone, there was a consistent demand for additional diagnostic devices for caries detection and diagnostics in pits and fissures. In addition to VE, conventional bitewing radiography (conventional BWR), digital bitewing radiography (digital BWR) and laser fluorescence (LF) measurements [7] were used in clinical practice or specifically introduced on the dental market in order to overcome the limitations of visual and/or tactile examination as well as to image and/or quantify the caries process to a certain degree [8]. On the basis of the acquired diagnostic information, the clinician should be enabled to make individual decisions about caries monitoring, prevention and/or operative intervention [9–11].

Numerous in vitro and in vivo caries detection, diagnostic, assessment and/or monitoring studies have been designed, conducted and published during the last few decades to describe the diagnostic performance of test methods in terms of validity (the diagnostic accuracy in relation to a reference standard) and intra-/inter-examiner reliability (the reproducibility of a diagnosis between different time points and examiners). Most recently, systematic reviews and meta-analyses have merged the available data and drawn conclusions mainly separately for each diagnostic method [12–16]. In addition, this author group [13–15] has mentioned substantial heterogeneity between the included diagnostic studies, and problematically, little attention has been paid to this important methodological issue so far; therefore, potential methodological sources of bias might be undetected and, furthermore, may also potentially skew meta-analysis data. Regarding this aspect, each diagnostic trial should ideally be designed similarly and should use equal scientific standards and protocols to generate comparable results that decrease the risk of bias (RoB) as much as possible. In contrast, previously published systematic reviews describe and report heterogeneity but do not exclude studies with a potentially high RoB. Therefore, this systematic review of the literature and meta-analysis was aimed, first, to identify caries diagnostic studies on pits and fissures that are tested with commonly used diagnostic methods, second, to evaluate study quality and identify only those studies with low/moderate RoB and, finally, to provide meta-analytic data on the diagnostic performance of clinically relevant detection and diagnostic methods.

## Material and methods

The methodology of this systematic review was influenced by several recommendations or guidelines. The QUADAS 2 tool [17, 18], which was designed for the quality assessment of diagnostic accuracy studies, provided the basis for the RoB assessment. Here, the most recently published draft of the ‘Cochrane Handbook for Diagnostic Test Accuracy Reviews’ was also used [19]. The writing of this systematic review strictly followed the PRISMA-DTA statement (Preferred Reporting Items for Systematic Reviews and Meta-Analyses of Diagnostic Test Accuracy Studies) for diagnostic studies in its latest version [20]. The PRISMA-DTA group developed criteria to evaluate the validity and applicability of diagnostic studies and to enhance the replicability of systematic reviews in this area. The present systematic review was registered on the PROSPERO platform (CRD42017069894).

### Search strategy

The research question, inclusion and exclusion criteria and search strategy were conducted on the basis of the PIRD concept [21]. Basically, this systematic review of the literature included in vitro and in vivo diagnostic studies that tested the diagnostic accuracy and/or reliability of different diagnostic methods for primary caries detection and assessment in human permanent posterior teeth (premolars and molars). In vivo studies were included regardless of the age of the population and the number of included patients or teeth. Studies containing information on primary teeth or teeth with restorations, secondary caries or artificially induced caries lesions were excluded. With respect to its clinical relevance, the following index tests were included in the search: VE, conventional BWR, digital BWR, LF measurements (DIAGNOdent 2095 or 2190, KaVo, Biberach, Germany), fibre-optic transillumination (FOTI, IC Lercher, Stockach, Germany) and quantitative light-induced fluorescence (QLF, Inspektor Research Systems, Amsterdam, The Netherlands). Other index test methods were not considered in this review. An essential characteristic of studies on diagnostic accuracy was the inclusion of a reference test, frequently also named the ‘gold standard’ or ‘reference standard’. The included in vitro studies had to use any histological technique to validate the ‘true’ caries extension; otherwise, the studies were excluded. Under in vitro conditions, several histological techniques, e.g. slices, grinding, hemisection or microradiography, are well-established which fulfil the before-mentioned prerequisite. In clinical studies, cavity preparation or biopsy can be considered equivalent to provide proof about the presence of any (dentin) caries [22]. As dental radiography was commonly applied under clinical conditions as well, it was, therefore, also included [23, 24]. In relation to the previously formulated aims and the corresponding inclusion and exclusion criteria, a structured search of the literature was initiated in accordance with the mnemonic PIRD recommendations [21]. This concept included information about the study material or population, the selected index tests, possible reference tests and diagnoses of interest (outcomes). The final consented search items are shown in Table 1.

**Table 1:** Documentation of keywords according to the PIRDS concept (Campbell et al. 2015)

Population/problem (P)		Index test (I)		Reference test (R)		Diagnose and study type (D/S)
Caries Decay AND Occlusal Fissure	AND	Visual Clinical Clinically Inspect* ICDAS Bitewing Conventional Radiography Digital Radiography Radiogra* Film Analo* X ray Xray Speed Roentge* Radiology Radiol* Laser fluorescence Diagnodent FOTI DiFOTI Di(FOTI) Fiber Fibre Transillumination Optic Opti* QLF Quantit* Laser Light Induced	AND	Validity Validation Valid* Accuracy Sensitivity Specificity SE SP ROC Az Reproducibility Reproducib* Reliab* Reliability Kappa Threshold Cut off Performance Histolog* Micro Micro computed CT *CT	AND	Systemati* Review Meta-Analysis Diagnos* Diagnost* Detection Detect Detect* Assessm* Vivo Vitro Study Studies

MeSH terms which were used to search the PubMed and EMBASE databases: ((Caries or Decay) AND (Occlusal or Fissure) AND (Visual or Clinical or Clinically or Inspect* or ICDAS or Ekstrand or Bitewing or Conventional or Digital or Radiography or Film or Radiogra* or Analo* or Speed* or X Ray or Xray or Radiology or Radiol* or Roentge* or Laser or Fluorescence or Diagnodent or FOTI or DiFOTI or Fiber or Fibre or Transillumination or Optic or Opti* or QLF or Quantit* or Laser or Light or Induced) AND (Validity or Validation or Valid* or Accuracy or Sensitivity or Specificity or SE or SP or ROC or Az or Reproducib* or Reproducibility or Reliability or Reliab* or Kappa or Threshold or Cutoff or Performance or Histolog* or Micro or Micro-computed or CT or *CT) AND (Systemat* or Review or Meta-Analysis or Diagnos* or Diagnost* or Detection or Detect or Detect* or Assessm* or Vivo or Vitro or Study or Studies))

### Basic literature search and study selection according to PRISMA recommendations

The systematic search of the literature was performed in the MEDLINE (via PubMed) and EMBASE (via Ovid) electronic literature databases using the consented search terms (Table 1) according to standard procedures [20, 25]. The search included all publications that were listed until 31 December 2018 in the databases and were written in English. Grey literature was not included. Additionally, reference lists of included studies and reviews were screened to identify any studies that may have been missed. A few studies (*N* = 4) were found in result of manual searches.

### Identification of the relevant literature

All identified bibliographies (PubMed *N* = 946, EMBASE *N* = 836), including titles and abstracts, were exported to a bibliographic software package (X7.8 for Windows, Thomson Reuters). The imported set of records from each database, including hand searches, was merged into one core database to remove duplicate records and to facilitate retrieval of relevant articles. In the next step, duplicates (*N* = 696) were removed, and the title (and, if needed, the abstract of each bibliography) was checked as to whether it met the inclusion criteria; otherwise, the study was excluded. After the primary identification of includable studies and the removal of duplicates, 1090 records were identified.

### Screening and eligibility check

The titles and abstracts were screened by two reviewers (SK, MJR) independently. The reviewers were not blinded to the names of the authors, institutions, journal or results of each publication. All records were counterchecked in relation to the initially consented inclusion and exclusion criteria. If papers met the inclusion criteria completely or partially, their full-text documents were obtained. Doubts or disagreements were continuously resolved by discussion with an experienced researcher (JK). After review of the titles and abstracts, records that were found to be irrelevant were excluded from further proceedings (*N* = 894). At this step, 196 records were identified for full-text reading. Studies (*N* = 56) that were found to be irrelevant after their full texts were read were excluded from further analysis (supplemental Table S0). Finally, 140 studies met the inclusion criteria and were read in detail.

### Data collection from the selected studies

Following the recommendation for diagnostic test accuracy [26], the following relevant items were extracted: study type (in vivo or in vitro studies), study population and teeth (number and age of patients, type and number of permanent teeth used in the study), index test methods (methods, scoring criteria and cut-offs), reference standard method (type of histological validation method, scoring criteria and cut-offs), validity and/or intra- and inter-examiner reliability data for the overall caries detection level (D0 versus D1-D4; Marthaler 1966), dentin caries detection level (D0-2 versus D3-4, Marthaler 1966) [27] and 1/3 dentin caries detection level (D0-2 versus D3-4, Ekstrand et al. 1997) [28]. Two reviewers (SK, MJR) independently extracted the required data from all primary studies. Any doubts or disagreements were continuously resolved by discussion with an experienced researcher (JK) until a consensus was reached. All data were systematically entered into an EpiData database [29] (EpiData software version 2.0.9.57, EpiData Association, Denmark).

### RoB assessment

To date, no suitable set of criteria exists for assessing RoB among caries diagnostic studies. Therefore, existing checklists and proposals [21, 30–32] were analysed and adapted to clinical/laboratory caries diagnostic studies. The developed set of criteria includes 16 signalling questions divided into four main domains used for RoB assessments during the review (supplemental Table S7). Using the RoB assessment tool, all included studies were re-evaluated and assessed independently by two reviewers (SK, MJR). An additional and blind assessment was performed by two other colleagues from the workgroup (FE, SM). All RoB assessments are listed in supplemental Tables S8a/b–S13a/b.

In addition to the initially performed systematic search and selection of the literature, all identified papers were further selected according to their RoB status. Here, seven core domains were selected (tooth selection, index test criteria, reference test criteria, incorporation bias, partial verification bias, differential verification bias, bias in the analysis), and each study had to show a low or moderate inclusion in these domains; otherwise, the study was excluded from further analysis. In the next step, the remaining studies were crosschecked for the availability of sufficient validity data reporting cross-tabulation, sensitivity (SE), specificity (SP), positive predictive (PPV), negative predictive values (NPV) or areas under the receiver operating characteristic curve (AUC).

### Data handling, statistical procedures and meta-analysis

All data were entered into a database and later exported to an Excel spreadsheet (Excel 2010, Microsoft Corporation, Redmond, WA, USA). Descriptive analyses were performed using Microsoft Excel 2010 and the statistical package mada version 0.5.9. [33] for RStudio [34]. If the included studies provided contingency tables, the data were used directly. If not, true positives (SE), true negatives (SP), PPV and NPV were calculated from the results in the original publication. If this calculation was not possible, the corresponding study was excluded. Corrections of tables with zero cells were also made; when, for example, the value for the true positives is zero, *R* itself makes a correction by changing the zero to 0.5 (a very small number) because RStudio cannot deal with zero cells. In some reports, statistical information was given for more than one examiner. However, in those cases, a mean was calculated by logit transformation.

Meta-analytic statistics were calculated for all included diagnostic test methods and commonly used diagnostic thresholds. Diagnostic accuracy and their 95% confidence intervals (95% CI) were calculated from the pooled data from all included studies, in terms of SE, SP and the diagnostic odds ratio (DOR). A bivariate diagnostic random-effects meta-analysis suggested by Reitsma et al. [35] was used to provide pooled estimates of SE and SP for the respective subgroups along with their 95% CI. This method can take the heterogeneity between studies into account by jointly analysing the logit transformation of SEs and SPs [36]. Finally, the pooled DOR was calculated using a random-effects model following the approach by DerSimonian and Laird [37] and aimed at describing the performance of the included diagnostic tests. An uninformative test shows a DOR value of 1; as the DOR increases, the test has more discriminatory power [38]. The area under the curve (AUC) of summary receiver operating characteristics (sROC) was reported to create an overview of the results within each subgroup. The AUC value quantifies the overall ability of a diagnostic test to discriminate between individuals with the disease and those without the disease [39]. The ideal test would have an AUC value of 1, whereas a random guess would have an AUC of 0.5; the larger the area under the ROC curve, the more accurate the diagnostic test. In addition, sROC plots and forest plots were computed to illustrate the diagnostic performance and heterogeneity, respectively [39].

## Results

**Fig. 1:**
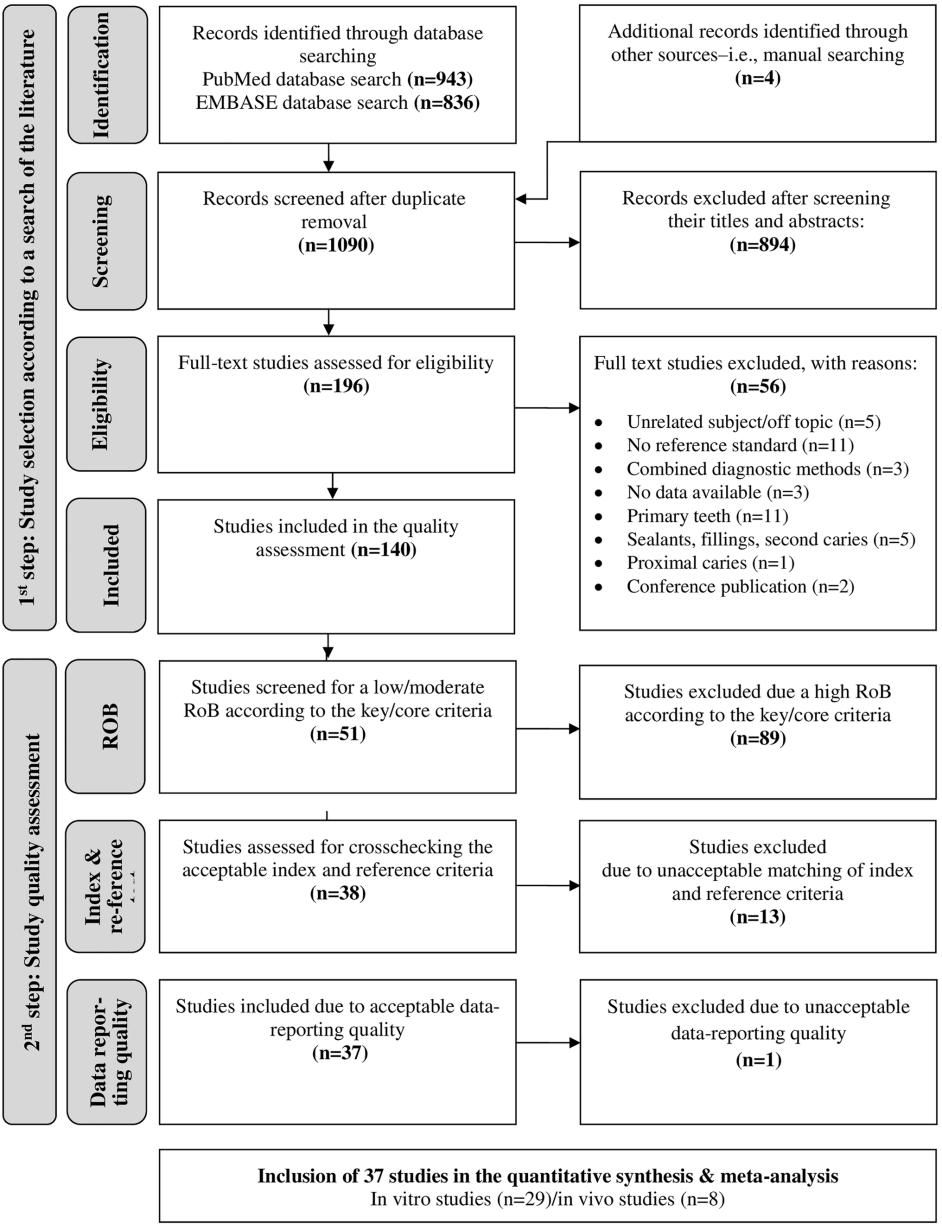
Flow diagram detailing our search and study selection process applied during the systematic literature search (*1st step*) and study quality assessment (*2nd step)*

**Fig. 2:**
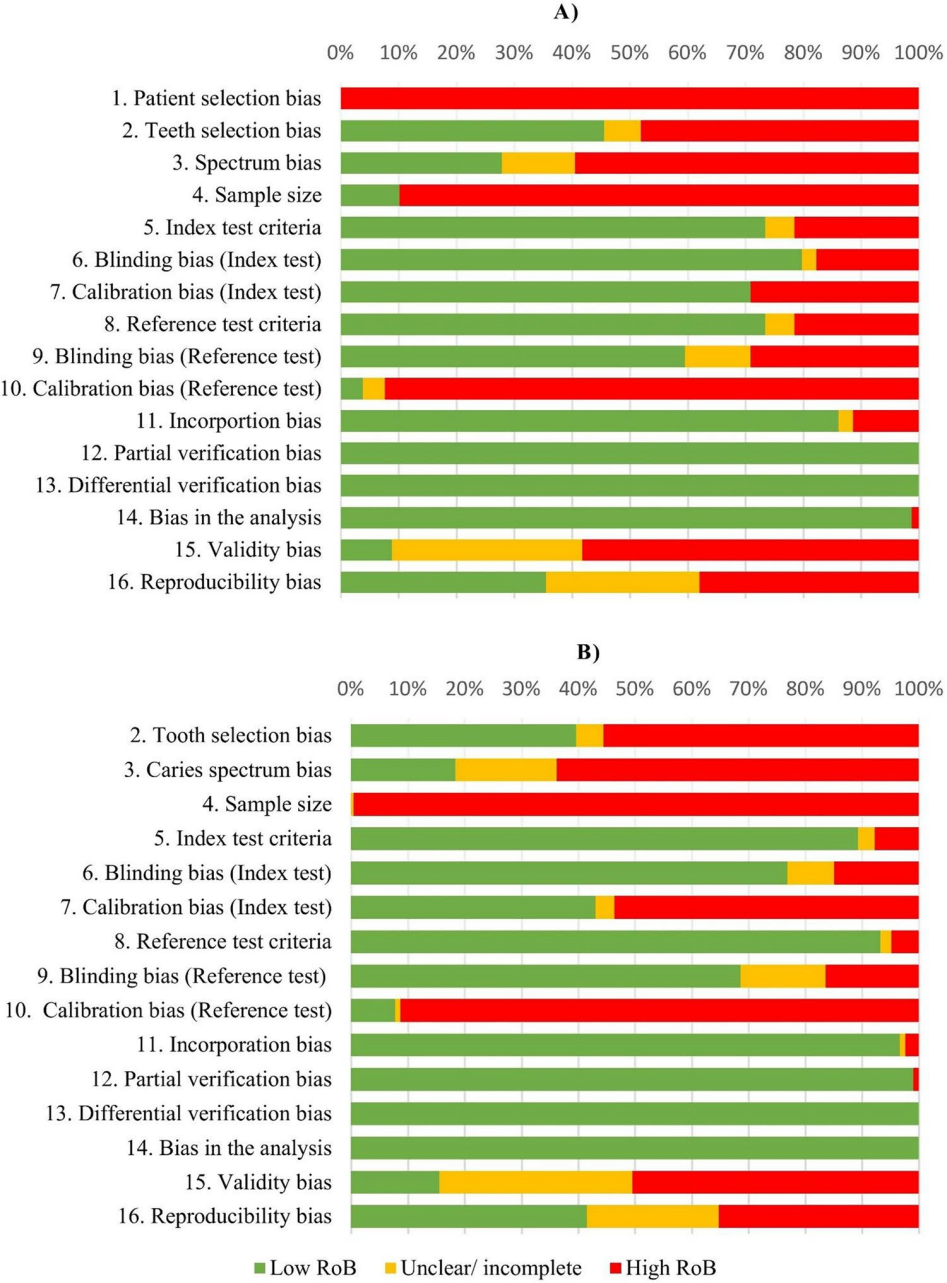
RoB graph across included in vivo (**A**) and in vitro (**B**) caries diagnostic studies for occlusal surfaces. Item no 1 (*patient selection bias)* is only available for clinical diagnostic studies

According to the workflow recommended by the PRISMA guidelines, 140 (108 in vitro and 32 in vivo) studies were initially identified (Fig. 1). After further consideration of the results from the RoB assessment (supplemental Tables S8a/b–S13a/b), an additional 103 publications needed to be excluded due to high RoB or insufficient data reporting (supplemental Tables S8c/d–S13c/d); the summary graphs from the RoB assessment are depicted in Fig. 2. Finally, 29 in vitro and 8 in vivo studies [40–76] were selected according to the described stepwise process and were found to fulfil the inclusion criteria for meta-analysis (Fig. 1, Table 2). Only two studies were identified to use FOTI, and none used QLF.

**Table 2:** Overview of the identified diagnostic studies in relation to the method used and characteristics of the study set-up with stepwise included studies for meta-analysis

1st step			2nd step		
Study inclusion according to the systematic search of the literature			Study inclusion according to the quality assessment		
Studies on diagnostic methods	Study set-up	Specification (N according to PRISMA)	Low/moderate RoB	Acceptable index and reference test	Acceptable data reporting quality
VE (*N* = 106)	In vivo (*N* = 27)	Without a probe (*N* = 22)	10	4	3
		With a probe (N = 5)			
	In vitro (*N* = 79)	Without a probe (*N* = 66)	23	14	13
		With a probe (*N* = 13)			
Conventional bitewing radiography (*N* = 63)	In vivo (*N* = 18)	D-speed (*N* = 10)	3	2	1
		E-speed (*N* = 3)	2	2	2
		F-speed (*N* = 1)	-	-	-
		Not specified (*N* = 4)	1	-	-
	In vitro (*N* = 45)	D-speed (*N* = 13)	4	3	3
		E-speed (*N* = 24)	5	2	2
		F-speed (*N* = 6)	2	2	2
		Not specified (*N* = 7)	1	1	1
Digital bitewing radiography (*N* = 19)	In vivo (*N* = 3)	Sensor (*N* = 0)	-	-	-
		Phosphor plate (*N* = 1)	-	-	-
		Not specified (*N* = 2)	1	-	-
	In vitro (*N* = 16)	Sensor (*N* = 9)	3	2	2
		Phosphor plate (*N* = 8)	2	1	1
		Not specified (*N* = 0)	-	-	-
LF measurement (*N* = 68)	In vivo (*N* = 22)	DIAGNOcam 2095 (*N* = 22)	9	3	3
		DIAGNOcam 2190/Pen (*N* = 5)	2	-	-
	In vitro (*N* = 46)	DIAGNOcam 2095 (*N* = 38)	18	10	10
		DIAGNOcam 2190/Pen (*N* = 12)	7	6	5
Fibre-optic transillumination (*N* = 8)	In vivo (*N* = 1)		-	-	-
	In vitro (*N* = 7)		3	3	3
Quantitative light-induced fluorescence (*N* = 7)	In vivo (*N* = 1)		1	-	-
	In vitro (*N* = 6)		2	-	-

Meta-analytic validity data are presented for all included caries detection and diagnostic methods in relation to the three chosen caries detection levels for laboratory and clinical studies in Tables 3 and 4, respectively. Most data sets originated from in vitro studies (*N* = 29, Table 3) rather than clinical investigations (*N* = 8, Table 4). In the in vitro results for all diagnostic methods at the caries detection and dentin caries level, a higher SP than SE value was typically found (Table 3). AUCs were characteristically higher for additional diagnostic methods, e.g. radiography or LF, than for VE. The highest diagnostic performance was observed for VE at the 1/3 dentin caries detection level (AUC = 0.89). The DOR values ranged from 1.94 to 37.77 (dentin caries detection level/in vitro, Table 3), 2.14 to 60.37 (caries detection level/in vivo, Table 4) and 11.79 to 127.56 (dentin caries detection level/in vivo, Table 4).

**Table 3:** Bivariate diagnostic random-effects meta-analysis for the finally included in vitro studies for all diagnostic methods at different caries detection levels

Meta-analytical diagnostic performance		In vitro		
		Caries detection level	Dentin detection level	1/3 dentin detection level
VE	N SE (95% CI) SP (95% CI) AUC (Reitsma) DOR	3 0.59 (0.52–0.67) 0.83 (0.70–0.92) 0.59 5.55 (1.88–16.38)	8 0.46 (0.20–0.73) 0.87 (0.72–0.95) 0.79 5.93 (3.11–11.31)	2 0.69 (0.51–0.82) 0.88 (0.83–0.92) 0.89 16.6 (4.85–56.79)
Conventional bitewing radiography (D-speed)	N SE (95% CI) SP (95% CI) AUC (Reitsma) DOR	-	1 0.42 (0.18–0.69) 0.73 (0.53–0.87) 0.60 1.94 (0.46–8.17)	-
Conventional bitewing radiography (E-speed)	N SE (95% CI) SP (95% CI) AUC (Reitsma) DOR	-	2 0.48 (0.21–0.77) 0.95 (0.53–0.997) 0.75 10.69 (3.67–31.15)	-
Conventional bitewing radiography (F-speed)	N SE (95% CI) SP (95% CI) AUC (Reitsma) DOR	-	2 0.50 (0.22–0.79) 0.97 (0.71–0.998) 0.82 23.60 (8.28–67.24)	-
Digital bitewing radiography (phosphor plates)	N SE (95% CI) SP (95% CI) AUC (Reitsma) DOR	-	2 0.48 (0.24–0.73) 0.95 (0.59–0.995) 0.73 15.57 (0.47–515.27)	-
LF 2095	N SE (95% CI) SP (95% CI) AUC (Reitsma) DOR	6 0.75 (0.58–0.86) 0.76 (0.60–0.87) 0.81 10.28 (4.35–24.28)	7 0.68 (0.54–0.79) 0.78 (0.68–0.85) 0.79 8.01 (4.04–15.88)	-
LF pen 2190	N SE (95% CI) SP (95% CI) AUC (Reitsma) DOR	2 0.78 (0.44–0.94) 0.77 (0.62–0.87) 0.77 11.83 (2.66–52.63)	4 0.63 (0.37–0.83) 0.77 (0.62–0.88) 0.78 5.85 (1.77–19.30)	-
Fibre-optic transillumination FOTI	N SE (95% CI) SP (95% CI) AUC (Reitsma) DOR	1 0.97 (− 0.92–0.99) 0.50 (0.34–0.66) 0.92 38.33 (10.15–144.77)	2 0.49 (0.20–0.79) 0.97 (0.89–0.994) 0.92 37.77 (13.69–104.19)	-

**Table 4:** Bivariate diagnostic random-effects meta-analysis for the finally included in vivo studies for all diagnostic methods at different caries detection levels

Meta-analytical diagnostic performance		In vivo		
		Caries detection level	Dentin detection level	1/3 dentin detection level
VE	N SE (95% CI) SP (95% CI) AUC (Reitsma) DOR	2 0.70 (0.59 − 0.80) 0.47 (0.26 − 0.70) 0.70 2.14 (0.73 − 6.28)	-	3 0.72 (0.52 − 0.86) 0.77 (0.67 − 0.85) 0.77 10.18 (3.94 − 26.29)
Conventional bitewing radiography (D-speed)	N SE (95% CI) SP (95% CI) AUC (Reitsma) DOR	1 0.65 (0.57 − 0.73) 0.58 (0.42 − 0.72) 0.65 2.59 (1.24 − 5.44)	2 0.79 (0.41 − 0.96) 0.75 (0.68 − 0.82) 0.77 11.79 (2.43 − 57.24)	-
Conventional bitewing radiography (E-speed)	N SE (95% CI) SP (95% CI) AUC (Reitsma) DOR	1 0.80 (0.71 − 0.87) 0.94 (0.46 − 0.996) 0.94 60.37 (3.31v1100.70)	2 0.76 (0.61 − 0.87) 0.98 (0.79 − 0.998) 0.90 127.56 (7.38 − 2203.70)	-
Conventional bitewing radiography (F-speed)	N SE (95% CI) SP (95% CI) AUC (Reitsma) DOR	-	-	-
Digital bitewing radiography	N SE (95% CI) SP (95% CI) AUC (Reitsma) DOR	-	-	-
LF 2095	N SE (95% CI) SP (95% CI) AUC (Reitsma) DOR	1 0.88 (0.81 − 0.93) 0.71 (0.55 − 0.83) 0.88 18.33 (7.57 − 44.37)	2 0.91 (0.86 − 0.95) 0.78 (0.46 − 0.94) 0.92 35.90 (13.43 − 96.00)	-
LF pen 2190	N SE (95% CI) SP (95% CI) AUC (Reitsma) DOR	-	-	-
Fibre-optic transillumination FOTI	N SE (95% CI) SP (95% CI) AUC (Reitsma) DOR	-	-	-

A meta-analysis was conducted for in vivo studies as well (Table 4). Here, SE (0.70) was registered to be higher than SP (0.47) for VE at the caries detection level. The SE (0.72) and SP (0.77) were higher at the 1/3 dentin caries detection level. The meta-analytic diagnostic performance of conventional bitewing radiography (F-speed) and LF was found to be excellent.

In addition to the fact that comparisons between in vitro and in vivo studies should be performed with caution with respect to the imbalance of included studies, a few trends were observed. While on the one hand, the diagnostic performance of VE tended to be higher under laboratory conditions than in clinical settings, on the other hand, the diagnostic performance of VE was not perfect and was lower than that of additional diagnostic methods. Here, conventional radiography (E-speed) and LF measurements showed higher performance data under clinical conditions. Furthermore, for all methods, there seemed to be a tendency towards a higher SE in clinical studies. SP was found to be comparable under laboratory and clinical conditions; only in the case of VE were higher values registered in vitro. Again, full comparisons could not be made due to incompleteness of the data (Tables 3 and 4). In addition, SROC curves and forest plots were computed and are presented in the additional online material (supplemental Tables S14–S17).

## Discussion

This study project summarized the diagnostic accuracy of occlusal caries lesion detection, diagnostic, assessment and/or monitoring methods that were investigated under in vitro and in vivo conditions in permanent, posterior teeth. Therefore, a systematic search of the literature was conducted; potential sources of bias were considered; and finally, a meta-analysis was performed to compare commonly used caries diagnostic methods instead of analysing each method separately [12–16, 77–81]. When considering the quantity and quality of the systematically searched literature, it should be noted that there was a remarkable reduction in includable studies with each additional selection step (Fig. 1). Finally, 37 studies were included in the meta-analysis [40–76], and unfortunately, these studies were not equally distributed over all test methods, study setups and considered thresholds (Tables 2, 3 and 4). Most studies were conducted under laboratory conditions (Fig. 1, Table 2) and investigated the diagnostic accuracy using the dentin caries detection threshold (Tables 3 and 4). VE, BWR and LF were tested most frequently than other additional diagnostic methods. This heterogenetic information pattern suggests that it is substantially necessary to conduct caries diagnostic studies that include different test methods and thresholds on pits and fissures. This demand is even more crucial for clinical studies.

The diverging methodology of each trial—technologies, thresholds, index and reference test criteria (supplemental Tables S1–S6)–and several sources of bias (Fig. 2, supplemental Tables S7–S13b) resulted in the exclusion of numerous studies, which ultimately lowered the number of includable studies and illustrated the heterogeneity between studies. This fact underlines the need for standardization and the necessity to conduct well-designed and well-powered caries diagnostic and detection studies in the future.

Regarding the meta-analytic diagnostic performance of the included diagnostic methods (Tables 3 and 4), it must be emphasized that for some methods, only a limited number of studies were identified. Exceptions were VE, BWR and LF (Tables 3 and 4). When viewing these data, a few trends can be discussed, but it should be mentioned from the outset that the results of this meta-analysis should not be overrated due to the limited number of includable studies for each of the relevant caries detection categories (Table 2). Nevertheless, a few conclusions can be drawn from the available data. The data support the generally and repeatedly published assumption that VE of pits and fissures is not perfect and needs to be accompanied by additional diagnostic methods. Nevertheless, more recently published criteria (ICDAS, UniViSS) that summarize the whole spectrum of non-cavitated caries lesions may help to overcome this drawback [16, 82–84]. Under in vitro conditions, VE showed mostly high SP values, while SE varied between the different methods and thresholds. A large difference between SE values was registered for VE under in vitro and in vivo conditions (Tables 3 and 4), which was also reported by Gimenez et al. [15]. Therefore, VE under in vitro conditions results in higher SP values. Vice versa, clinical evaluations probably include more details, which may result in higher diagnostic SE values especially for enamel caries.

It should be further noted that VE is the method that enables the clinician to collect important diagnostic co-variables, e.g. presence of biofilm or lesion appearance, enables differential diagnoses and provides finally information about the caries lesions activity [85, 86]. The latter aspect potentially influences the individual caries management strategy and it’s consideration has become mandatory in clinical practice [87–89]. Contrary, with respect to the methodological difficulties and missing standards to validate caries activity, it was decided to exclude the activity assessment from the present systematic search of literature and meta-analysis.

In vitro data from Ekstrand and co-workers [28, 90, 91] pointed to the fact that non-cavitated occlusal lesions depth (histological assessed), either, was restricted to the enamel or penetrated the dentin, but then restricted to the outer 1/3 towards the pulp. To raise the accuracy, e.g. in terms of SE and SP, Ekstrand et al. [28] suggested to move the standard thresholds - enamel versus dentin caries - to lesions reaching the middle or inner 1/3 of the dentin. Thus, combined SP and SP values amounted to 175 [91]. The new threshold is much more relevant to the clinicians than the old one, as non-cavitated lesions without an obvious shadow should receive non-operative care if the lesions are assessed as active, while more mature active lesions should receive operative [16].

BWR is the most commonly used additional caries lesion detection method in daily dental practice. However, its validity on occlusal surfaces is often questioned, especially in the early stages of caries [92]. Here, the anatomy of the tooth crown results in superimposed images on the two-dimensional (bitewing) radiographs, making the detection of early dentin caries lesions harder in comparison to that on proximal sides [93]. Surprisingly, the results of the present meta-analysis did not show a striking difference in SE and SP values between different X-ray types assessed in this review. However, the difference in accuracy parameters was obvious compared to those of LF. However, due to the limited number of studies belonging to each BWR category, these results should be interpreted with caution. Unlike previously published reviews [13], this review considered separate studies using conventional film-based BWR and digital BWR (including their different modalities) with the aim of reducing bias. Unfortunately, this approach resulted in a low number of includable studies in each category.

LF has been used as an adjunct caries detection method for incipient lesions that otherwise could not be detected by VE alone [94]. The results of our study revealed high SE and SP values for LF under in vitro conditions, which is in line with previously reported findings by Gimenez et al. [14] and Rosa et al. [12]. When considering the small number of includable data from in vivo studies (Table 4), these data should be treated with caution, but they are still comparable to previous findings from Pinheiro et al. [94]. In contrast to these reassuring results, LF alone is not sufficient for the correct diagnosis of caries and good standardization is essential to avoid overtreatment and false-positive readings due to other fluorescence sources [12, 14, 81, 84, 94].

The present study has strengths and limitations. First, one strength is that commonly used diagnostic methods for occlusal caries detection and diagnostics were analysed in one meta-analysis. Second, there was a strict study selection protocol, which was based on principles for performing systematic reviews and, in addition, a tailored RoB assessment that included only studies with a low RoB and excluded probable heterogenic publications. Third, the present study considered different thresholds independently for in vitro and in vivo studies. As a main limitation of the study selection process used, the exclusion of reports with a potentially high RoB from the meta-analysis and feasibly subjectivity of included selection criteria might be discussed for very low number of the included studies, especially in the clinical research. To our knowledge, such strict selection has not previously been performed because it is not part of the current recommendations for conducting a meta-analysis. While this step may result in the analysis of a homogenous pool of studies, it resulted, by contrast, in a substantial reduction in includable studies. It is further worth mentioning that several reports needed extensive discussion with respect to missing data or information. Therefore, the inclusion or exclusion of a single study remained in some cases a subjective procedure that could not be fully objectified. Because of the limited number of includable studies and the low sample size, the results from this meta-analysis should be interpreted with caution. This fact underlines the urgent need for well-designed and well-powered diagnostic studies that use multiple diagnostic procedures and different caries thresholds.

## Conclusions

There is an overall need for high-quality, well-designed and standardized studies on the detection, diagnosis, assessment and/or monitoring of occlusal surface caries. This need must be emphasized for diagnostic studies under in vivo conditions due to the limited number of clinical trials and the documented heterogeneity between published reports. When considering the meta-analytic results, VE, BWR and LF provide acceptable measures for their diagnostic performance on occlusal surfaces. Again, the present results should be interpreted with caution with respect to the limited data in many diagnostic categories.

## Supplementary Information

Below is the link to the electronic supplementary material.

Supplementary file1 (DOCX 1721 KB)
