# Supplementary material for: Systematic review and meta-analysis of diagnostic methods for occlusal surface caries
Source: Clin Oral Investig. 2021 Jun 14;25(8):4801–15. doi: 10.1007/s00784-021-04024-1 (PMC8342337; doi:10.1007/s00784-021-04024-1)
Supplement: Supplementary file 1 — Supplementary file1 (DOCX 1721 KB) [file 784_2021_4024_MOESM1_ESM.docx]

Supplemental content

**List of Content**

[1 Methodological background information 5](#_Toc41061144)

[Table S0 List of excluded studies with the reasons for exclusion from the systematical review and meta-analysis 6](#_Toc41061145)

[Table S1 Overview on frequently used semiquantitative classification criteria for the reference standard in validated caries diagnostic studies 7](#_Toc41061146)

[Table S2 Visual diagnostics criteria for caries detection on occlusal surfaces 10](#_Toc41061147)

[Table S3 Bitewing radiography diagnostics criteria for caries detection on occlusal surfaces 14](#_Toc41061148)

[Table S4 Digital bitewing radiography diagnostics criteria for caries detection on occlusal surfaces 15](#_Toc41061149)

[Table S5 Laser fluorescence criteria for caries detection on occlusal surfaces 16](#_Toc41061150)

[Table S6 FOTI criteria for caries detection on occlusal surfaces 17](#_Toc41061151)

[Table S7 QLF criteria for caries detection on occlusal surfaces 18](#_Toc41061152)

[2 Results from the study selection process according to PRISMA 19](#_Toc41061153)

[Table S1a Results of systematic search of the literature on occlusal surfaces for in vitro validation studies on visual examination 20](#_Toc41061154)

[Table S1b Results of systematic search of the literature on occlusal surfaces for in vivo validation studies on visual examination 23](#_Toc41061155)

[Table S2a Results of systematic search of the literature on occlusal surfaces for in vitro validation studies on conventional radiography 24](#_Toc41061156)

[Table S2b Results of systematic search of the literature on occlusal surfaces for in vivo validation studies on conventional radiography 26](#_Toc41061157)

[Table S3a Results of systematic search of the literature on occlusal surfaces for in vitro validation studies on digital radiography 27](#_Toc41061158)

[Table S3b Results of systematic search of the literature on occlusal surfaces for in vivo validation studies on digital bitewing radiography 28](#_Toc41061159)

[Table S4b Results of systematic search of the literature on occlusal surfaces for in vivo validation studies on laser fluorescence 31](#_Toc41061160)

[Table S5a Results of systematic search of the literature on occlusal surfaces for in vitro validation studies on FOTI 33](#_Toc41061161)

[Table S5b Results of systematic search of the literature on occlusal surfaces for in vivo validation studies on FOTI 34](#_Toc41061162)

[Table S6a Results of systematic search of the literature on occlusal surfaces for in vitro validation studies on QLF 35](#_Toc41061163)

[Table S6b Results of systematic search of the literature on occlusal surfaces for in vivo validation studies on QLF 36](#_Toc41061164)

[3 Results from the quality assessment of the selected diagnostic studies 37](#_Toc41061165)

[Table S7 The set of criteria used for risk of bias (RoB) rating applied during the review 38](#_Toc41061166)

[Table S8a Risk of bias assessment for visual examination of in vitro validation studies on occlusal surfaces 44](#_Toc41061167)

[Table S8b Risk of bias assessment for visual examination of in vivo validation studies on occlusal surfaces 47](#_Toc41061168)

[Table S9a Risk of bias assessment for conventional bitewing radiography of in vitro validation studies on occlusal surfaces 49](#_Toc41061169)

[Table S9b Risk of bias assessment for conventional bitewing radiography of in vivo validation studies on occlusal surfaces 51](#_Toc41061170)

[Table S10a Risk of bias assessment for digital bitewing radiography of in vitro validation studies on occlusal surfaces 52](#_Toc41061171)

[Table S10b Risk of bias assessment for digital bitewing radiography of in vivo validation studies on occlusal surfaces 53](#_Toc41061172)

[Table S11a Risk of bias assessment for laser fluorescence measurements of in vitro validation studies on occlusal surfaces 54](#_Toc41061173)

[Table S11b Risk of bias assessment for laser fluorescence measurements of in vivo validation studies on occlusal surfaces 56](#_Toc41061174)

[Table S12a Risk of bias assessment for FOTI of in vitro validation studies on occlusal surfaces 58](#_Toc41061175)

[Table S12b Risk of bias assessment for FOTI of in vivo validation studies on occlusal surfaces 59](#_Toc41061176)

[Table S13a Risk of bias assessment for QLF of in vitro validation studies on occlusal surfaces 60](#_Toc41061177)

[Table S13b Risk of bias assessment for QLF of in vivo validation studies on occlusal surfaces 61](#_Toc41061178)

[Table S8c Finally included result- visual examination of in vitro validation studies on occlusal surfaces 62](#_Toc41061179)

[Table S8d Final result- visual examination of in vivo validation studies on occlusal surfaces 63](#_Toc41061180)

[Table S9c Final result- conventional bitewing radiography of in vitro validation studies on occlusal surfaces 64](#_Toc41061181)

[Table S9d Final result- conventional bitewing radiography of in vivo validation studies on occlusal surfaces 65](#_Toc41061182)

[Table S10c Final result- digital bitewing radiography of in vitro validation studies on occlusal surfaces 66](#_Toc41061183)

[Table S10d Final result- digital bitewing radiography of in vivo validation studies on occlusal surfaces 67](#_Toc41061184)

[Table S11c Final result- laser fluorescence of in vitro validation studies on occlusal surfaces 68](#_Toc41061185)

[Table S11d Final result- laser fluorescence of in vivo validation studies on occlusal surfaces 69](#_Toc41061186)

[Table S12c Final result- FOTI of in vitro validation studies on occlusal surfaces 70](#_Toc41061187)

[Table S12d Final result- FOTI of in vivo validation studies on occlusal surfaces 70](#_Toc41061188)

[Table S13c Final result- QLF of in vitro validation studies on occlusal surfaces 71](#_Toc41061189)

[Table S13d Final result- QLF of in vivo validation studies on occlusal surfaces 71](#_Toc41061190)

[4 Detailed results from the meta-analysis 72](#_Toc41061191)

[Table S1c Final result- visual examination of in vitro validation studies on occlusal surfaces 73](#_Toc41061192)

[Table S1d Final result- visual examination of in vivo validation studies on occlusal surfaces 75](#_Toc41061193)

[Table S2c Final result- conventional radiographic examination of in vitro validation studies on occlusal surfaces 76](#_Toc41061194)

[Table S2d Final result- conventional radiographic examination of in vivo validation studies on occlusal surfaces 77](#_Toc41061195)

[Table S3c Final result- digital radiographic examination of in vitro validation studies on occlusal surfaces 78](#_Toc41061196)

[Table S3d Final result- digital radiographic examination of in vivo validation studies on occlusal surfaces 79](#_Toc41061197)

[Table S4c Final result- laser fluorescence measurement of in vitro validation studies on occlusal surfaces 80](#_Toc41061198)

[Table S4d Final result- laser fluorescence measurement of in vivo validation studies on occlusal surfaces 83](#_Toc41061199)

[Table S5c Final result- FOTI of in vitro validation studies on occlusal surfaces 85](#_Toc41061200)

[Table 5d Final result- FOTI of in vivo validation studies on occlusal surfaces 85](#_Toc41061201)

[Table S6c Final result- QLF of in vitro validation studies on occlusal surfaces 86](#_Toc41061202)

[Table S6d Final result- QLF of in vivo validation studies on occlusal surfaces 86](#_Toc41061203)

[5 SROC Curve and Forest Plots 87](#_Toc41061204)

[Table S14 SROC for different caries diagnostic methods- in vitro validation studies on occlusal surfaces 88](#_Toc41061205)

[Table S15 SROC for different caries diagnostic methods- in vivo validation studies on occlusal surfaces 92](#_Toc41061206)

[Table S16 Forest plots (DOR) for different caries diagnostic methods- in vitro validation studies on occlusal surfaces 95](#_Toc41061207)

[Table S17 Forest plots (DOR) for different caries diagnostic methods- in vivo validation studies on occlusal surfaces 98](#_Toc41061208)

[6 References 100](#_Toc41061209)

# Methodological background information

## Table S0 List of excluded studies with the reasons for exclusion from the systematical review and meta-analysis

| **Reason for exclusion** | **Diagnostic study** |
| --- | --- |
| **Combined diagnostic methods** | Lussi et al. 1996, Hintze et al. 2002, Krzyzostaniak et al. 2014 |
| **No data available** | Wenzel et al.1990, Ekstrand et al. 1995, Hintze et al. 1995, Machiulskiene et al. 1999, Espelid et al. 2001, Heinrich-Weltzien et al. 2003, Galcera Civera et al. 2007 Kuhnisch et al. 2007, Alkurt et al. 2008, Rodrigues et al. 2012 Ertas et al. 2014, Bottenberg et al. 2016, Abreu et al. 1999, Peycheva et al. 2016, Ramezani et al. 2016 |
| **Other or no reference standard** | Chong et al. 2003, Ekstrand et al. 2007, Boynton et al. 2009, Chu et al. 2009, Zafersoy-Akarslan et al. 2009, Chu et al. 2010, Kouchaji et al. 2012, Mendoza et al. 2012, Jablonski-Momeni et al. 2013 Abrams et al. 2017, Muller-Bolla et al. 2017 |
| **Out of subject** | Haak et al. 2004, Diniz et al. 2010, Ghaname et al. 2010, De Souza et al. 2014 |
| **Primary caries** | Braga et al. 2007, Maroto et al. 2008, Theocharopoulou et al. 2015, Zaidi et al. 2016 |
| **Sealants, fillings, secondary caries** | Duruturk et al. 2011, Pourhashemi et al. 2013 |
| **Proximal caries** | Lara-Capi et al. 2017 |
| **Conference publication** | Lupi-Pegurier et al. 2009, Matos et al. 2012, Gottlieb et al. 2014 |

## Table S1 Overview on frequently used semiquantitative classification criteria for the reference standard in validated caries diagnostic studies

| **No.** | **Criteria** | | | **No caries** | | **Enamel caries** | | | | | **Dentine caries** | | | | | | | | | |
| --- | --- | --- | --- | --- | --- | --- | --- | --- | --- | --- | --- | --- | --- | --- | --- | --- | --- | --- | --- | --- |
|  |  |  |  |  |  | **Outer half** | | **Inner half** | | | **Outer half** | | | | | **Inner half** | | | | |
| **1.** | Marthaler et al. (1966) | | | **D0**  Without demineralization – without caries | | **D1**  Demineralization on superficial enamel (up to the external half of the enamel) – caries in superficial enamel | | **D2**  Demineralization on deeper enamel (more than the internal half of the enamel without involving the DEJ) – caries in deep enamel | | | **D3**  Demineralization on the DEJ and up to the external half of the dentin – dentinal caries | | | | | **D4**  Demineralization involving the internal half of the dentin – dentinal caries | | | | |
| **2.** | Downer et al. (1975)  Ketley et al. (1993) | | | **S0**  No enamel demineralization or a narrow surface zone of opacity (edge phenomenon) | | **S1/2**  Enamel demineralization limited to the outer 50% of the enamel layer/ Demineralization involving the inner 50% of the enamel, up to the enamel-dentine junction | | | | | **S3**  Demineralization involving the outer 50% of the dentine | | | | | **S4**  Demineralization involving the inner 50% of the dentine | | | | |
| **3.** | Kay et al. (1988) | | | **0**  No dentinal decay | | | | | | | **1**  Dentinal decay | | | | | | | | | |
| **4.** | Nytun et al. (1992)  Gray et al. (1997) | | | **0**  No caries | | **1**  Enamel caries | | | | | **2**  Dentine caries | | | | | | | | | |
| **5.** | Lussi et al. (1991) | | | **1**  No caries | | **2**  Subsurface lesion | | **3**  Caries confined to enamel | | | **4**  Caries beyond the DEJ | | | | | | | | | |
| **6.** | Wenzel und Fejerskov (1992) | | | **0**  No caries | | **1**  Enamel caries | | | | | **2**  Caries reaching dentine, but involving just the outer half | | | | | **3**  Deep dentinal caries, half way or more to the pulp | | | | |
| **7.** | Wenzel et al. (1992) | | | **0/1**  No caries or caries confined to enamel | | | | | | | **2**  Caries in dentine just beyond DEJ | | | | | **3**  Caries deep in dentin halfway or more to the pulp | | | | |
| **8.** | | Tveit et al. (1994) | **0**  Sound enamel | | **1**  Lesion extending to less than half the enamel thickness | | **2/3**  Lesion extending beyond half the enamel thickness but not in dentin characterized by discoloration only/ characterized by softness with or without discoloration | | | **4/5/6**  Lesion extending into the outer third of dentine characterized by discoloration only the enamel lesion D2 or D3/ characterized by softness with or without discoloration | | | | | | | **7**  Lesion extending beyond half the dentin thickness,  characterized by softness with or without discoloration | | | |
| **9.** | | Ricketts et al. (1995a) | **0**  Sound | | 1  Caries into enamel only | | | | | 2  Caries into dentine | | | | | | | 3  Caries through to pulp | | | |
| **10.** | | Ekstrand et al. (1997) | 0  No enamel demineralization or a narrow surface zone of opacity  (egde fenomenon) | | 1  Enamel demineralization limited to the outer 50% of the enamel layer | | 2  Demineralization involving between 50% of the enamel and third of the dentine | | | 3  Demineralization involving the middle third of the dentine | | | | | | | | | | 4  Demineralization involving the inner third of the dentine |
| **11.** | | Ferreira-Zandona et al. (1998) | 0  Sound | | 1  Caries | | | | | | | | | | | | | | | |
| **12.** | | Ricketts et al. (1998)  Boye et al. (2002) | 0  Sound (caries-free) | | | | | | | 1  Caries into outer dentine | | | | | 2  Caries into inner dentine | | | | | 3*  Caries into pulp |
| **13.** | | Abreu et al. (1999) | 0  No demineralization | | 1  Outer enamel demineralization | | 2  Inner enamel demineralization | | | 3  Outer 1/3 demineralization of dentine | | | | | 4  Middle 1/3 demineralization of dentin | | | | | 5  Inner 1/3 demineralization of dentine |
| **14.** | | Grossman et al. (2002) | 0=Sound tooth | | 1= White lesion in outer half of enamel | | 2= White lesion extends to inner half of enamel but not beyond DEJ  3= Discolored lesion extends to inner half of enamel but not beyond DEJ | | | 4= Discolored lesion extends to outer half of dentine | | | | | | | | 5= Discolored lesion extends to inner half of dentine  6=Restored surface | | |
| **15.** | | Cortes et al. (2003) | 0  Sound | | 1  Outer enamel | | 2  Inner enamel | | 3  Demineralization at DEJ | | | | 4  Outer dentine | 5  Middle dentine | | | | | 6  Inner dentine | |
| **16.** | | Forgie et al. (2003) | 0  Sound | | 1 (2)  Enamel lesion (enamel cavity) | | | | | 3 (4)  Dentine lesion (dentine cavity) | | | | | | | | | | |
| **17.** | | Souza Zaroni et al. (2006) | 0  No caries | | 1  Incipient lesion in enamel | | | | 2  DEJ lesion | | | (3)  Superficial lesion in dentin (cavity) | | | | | | 4  Deep lesion in dentin | | |
| **18.** | | ICDAS II Classification (Ismail et al. (2007) | D0  Sound tooth | | D1  First visual change in enamel | | | | D2  Distinct visual change in enamel and/or localized enamel breakdown | | | D3  Underlying dark shadow from dentin | | | | | | (D4)  Distinct or extensive distinct cavity with visible dentin | | |
| **19.** | | Diniz Rodrigues et al. (2009) | D0/D1  No caries, or histological enamel caries limited to the outer half of the enamel thickness | | | | D2  Histological caries extending beyond the outer half, but confined to the enamel | | | D3  Histological dentinal caries limited to the outer half of the dentin thickness | | | | | | | | D4  Histological dentinal caries extending into the inner half of dentine thickness | | |
| **20.** | | Bussaneli et al. (2015) | 0  Healthy tooth | | 1  Enamel carious lesion | | | | 2  Carious lesion at the DEJ | | | | 3  Dentinal lesion | | | | | | | |
| *DEJ-dentin-enamel junction; *pulpal involvement; Downer criteria -originally used for approximal surfaces; () cavity* | | | | | | | | | | | | | | | | | | | | |

## Table S2 Visual diagnostics criteria for caries detection on occlusal surfaces

| **No.** | **Criteria** | **No caries** | **Enamel caries** | | | **Dentine caries** | | | | |
| --- | --- | --- | --- | --- | --- | --- | --- | --- | --- | --- |
|  |  |  | **Outer half** | | **Inner half** | **Outer half** | | | **Inner half** | |
| **1.** | Marthaler et al. (1966)/  5-Point anatomical scale | D0  No discoloration indicated a caries-free fissure | D1  Initial enamel lesion, a yellow to light brown discoloration | | D2  Deep enamel caries | D3  Dark brown was considered to correspond to dentinal caries | | | D4  Black discoloration  dentinal caries | |
| **2.** | Downer et al. (1975) **In vitro**: | D0  Sound - Normal texture of enamel | D1/2  Caries lesion in enamel - Opacity or discoloration, rough surface, without loss of substance | | | D3  Caries lesion in the outer half of dentin - Opacity or discoloration with microcavities and soft consistency and darkened dentin subsurface surrounding enamel fissure | | | D4  Caries lesion in the inner half of dentin. Opaque and soft fissure. Usually there is a loss of substance | |
|  | **In vivo:** Astvalddottr et al. (2004); Huth et al. (2008); Bozdemir et al. (2013)  Clinical lesion depth | 0  Sound enamel | 1  Enamel caries | | | 2  Shallow dentine caries | | | 3  Deep dentine caries | |
| **3.** | Pitts and Fyffe et al. (1988) | **0 = Surface sound**  No evidence of treated or untreated clinical caries (slight staining permitted) | **1 = Initial caries**  No clinically detectable loss of substance. There may be staining, discoloration, or rough spots in enamel, but where loss of substance cannot be positively diagnosed | | **2 = Enamel caries**  There is demonstrable loss of tooth surface in pits or fissures, but no undermined enamel, there is no evidence that cavitation has penetrated dentine | **3 = Caries of dentine**  There is the appearance of a carious lesion extending into dentine | | | **4* = Pulpal involvement**  Deep cavity | |
|  |  |  |  |  |  |  |  |  | *W*ith probable pulpal involvement* | |
| **4.** | Wenzel et al. (1990) | 0  No caries | 1  Caries in enamel but no cavity | | 2  Caries in enamel but broad dark line in fissure | 3  Small cavity | 4  Large cavity | | 5*  Very large cavity | |
|  |  |  |  |  |  |  |  |  | **Probably reaching the pulp* | |
| **5.** | Lussi et al. (1991) | 1  Fissure area had no caries | 2  Subsurface lesion | | 3  Caries confined to enamel | 4  Caries beyond the DEJ | | | | |
| **6.** | Nytun et al. (1992)  Lussi 1993 Costa et al. (2008) | - | 1  Enamel caries | | | 2  Dentin caries | | | | |
|  | **In vivo gold standard:**  Traneus et al. (2004); Akarsu et al. (2006); Costa et al. (2008); Bahroloomi et al. (2015) | 0  Sound enamel | 1  Enamel | | | 2  Dentine caries | | | | |
| **7.** | **In vivo**: Verdonshoot et al. (1992) | 0/1  No caries or caries restricted to enamel | | | | 2/3  Caries reaching DEJ or extending into dentin | | | | |
|  | **In vivo gold standard:** Verdonshoot et al. (1992); Abalos et al. (2009) | No dentinal decay | | | | Dentinal decay | | | | |
| **8.** | **“5 point- confidence rating scale”** (Wenzel et al. (1992), Verdonshoot et al. (1993), Hintze et al. (1994), Wenzel et al. (1995)) | Score 1 = Caries definitely absent  Score 2 = Caries probably absent  Score 3 = Unsure if present or absent  Score 4 = Caries probably present  Score 5 = Caries definitely present | | | | 1= Dentinal caries almost definitely not present,  2= Dentinal caries probably not present,  3= Unsure whether dentinal caries is present  4= Dentinal caries probably present,  5= Dentinal caries almost definitely present | | | | |
| **9.** | Wenzel und Fejerskov (1992) | 0  No caries | 1  Chalky/ and occasionally stained fissure indicative of a early enamel lesion | | | 2  Chalky and dark stained fissure and a greyish shadow indicate of a Dentinal lesion but with no evidence of cavitation along the fissure entrance | | (3)  Small surface defects microcavities | | |
| **10.** | Ketley et al. (1993) | v0= No visible caries | v1= Thin brown line in base of fissure no breakdown or visible change in walls of fissure  v2= One or both of the following: dark line at base of fissure, white change in walls of fissure  v3= No breakdown of enamel | | | | | | | |
| **11.** | Tveit et al. (1994) | O  The surface is sound | O1  White or brown discoloration of the enamel, but no clinical signs of cavity formation or defects in enamel | O2  Very small defect in the surface enamel (cavity formation) with or without a shadow or opacity in the enamel indicating caries in the underlying enamel | | O3  A small-size cavity formation with or without a shadow or opacity in the enamel indicating caries in the underlying dentin | | O4  Medium to large cavitation | | |
| **12.** | Ricketts et al. (1995a) | 0  Clinically sound | 1  Caries into enamel only | | | 2  Caries into dentine | | 3*  Caries through to pulp | | |
| **13.** | BASCD (Pitts et al. 1997) | 0  Sound  (caries-free) | 1  Arrested caries | | | 2  Having caries into dentine | | 3*  Having caries extending **into the pulp* | | |
| **14.** | Ekstrand et al. (1997)  Cortes et al. (2003)  Modified Ekstrand | 0  No or slightly change in enamel translucency after prolonged air drying, exposing the dentine | 1 = Opacity or discoloration hardly visible on the wet surface, but distinctly visible after air drying  2= Opacity or discoloration distinctly visible after air drying  3 = Localized enamel breakdown in opaque or discolored enamel and/or greyish discoloration from the underlying dentine  4 = Cavitation in opaque or discolored enamel exposing the dentine | | | | | | | |
| **15.** | Ferreira Zandona et al. (1998) | 2= Sound | 1= Caries | | | | | | | |
| **16.** | Nyvad et al. (1999) | Sound=Normal enamel translucency and texture (slight staining allowed in otherwise sound fissure) | 1 Active caries Surface of enamel is whitish/yellowish opaque with loss of luster; (intact surface) feels rough when the tip of the probe is moved gently across the surface; generally covered with plaque. No clinically detectable loss of substance. Smooth surface: Caries lesion typically located close to gingival margin. Fissure/pit: Intact fissure morphology; lesion extending along the walls of the fissure.  2. Active caries Same criteria as Score 1. Localized surface defect (microcavity) in enamel only. (surface discontinuity) No undermined enamel or softened floor detectable with the explorer.  3. Active caries Enamel/dentin cavity easily visible with the naked eye; surface of cavity feels soft (cavity) or leathery on gentle probing. There may or may not be pulpal involvement.  4 Inactive caries Surface of enamel is whitish, brownish or black. Enamel may be shiny and feels (intact surface) hard and smooth when the tip of the probe is moved gently across the surface. No clinically detectable loss of substance.Smooth surface: Caries lesion typically located at some distance from gingival margin. Fissure/pit: Intact fissure morphology; lesion extending along the walls of the fissure.  5 Inactive caries Same criteria as Score 4. Localized surface defect (microcavity) in enamel only (surface discontinuity) No undermined enamel or softened floor detectable with the explorer. 6 Inactive caries Enamel/dentin cavity easily visible with the naked eye; surface of cavity may be (cavity) shiny and feels hard on probing with gentle pressure. No pulpal involvement.  7 Filling (sound surface)  8 Filling + active caries Caries lesion may be cavitated or non-cavitated.  9 Filling + inactive caries Caries lesion may be cavitated or non-cavitated. | | | | | | | |
| *DEJ-dentin-enamel junction; *pulpal involvement; Downer criteria -originally used for approximal surfaces; () cavity* | | | | | | | | | |  |

| **No.** | **Criteria** | **No caries** | **Enamel caries** | | **Dentine caries** | |
| --- | --- | --- | --- | --- | --- | --- |
|  |  |  | **Outer half** | **Inner half** | **Outer half** | **Inner half** |
| **17.** | Alwas-Danowska et al. (2002)  Y.L.Ie et al. (1995) | 0  No caries lesion present | 1  Caries lesion restricted to the enamel | 2  Caries lesion reaching the DEJ | 3  Caries lesion in dentine | |
| **18.** | Tonioli et al. (2002) | 0/1  No caries/  no coalesced enamel or fissure lesion | 2  Caries confined to enamel | | 3  Caries beyond the DEJ | |
| **19.** | Anttonen et al. (2003) | 0  Sound normal enamel texture | 1 (Inactive) enamel caries  the fissure enamel is brownish or black; the surface feels hard and smooth; no loss of substance  2. (Active) enamel caries  the fissure enamel is opaque with loss of luster; the surface feels rough; no loss of substance  3. Dentinal caries  the fissure enamel is opaque and feels soft; the subsurface dentin around the fissure appears dark; often loss of substance, but not necessarily | | | |
| **20.** | Forgie et al. (2003) | 0  Sound | 1 (2)  Enamel lesion (cavity) | | 3 (4)  Dentine lesion (cavity) | |
| **21.** | Fung et al. (2004) | 0  Sound | 1  Caries | | | |
| **22.** | Mestriner et al. (2005) | 0  Absence of carious lesion | 1  Carious lesion in superficial enamel | 2  Carious lesion in deep  enamel | 3  Carious lesion in dentin without pulpal injury | 4*  Carious lesion in dentin **with pulpal injury* |
| **23.** | Erten et al. (2006) | 0  The occlusal surface was sound | 1/2  The occlusal surface had a subsurface or enamel lesion | | 3  The surface had a dentine carious lesion | |
| **24.** | **ICDAS-II classification** (Ismail et al. 2007) | 0  Sound | 1=First visual change in enamel (seen only after prolonged air drying or restricted to within the confines of a pit or fissure)  2=Distinct visual change in enamel  3=Localized enamel breakdown (without clinical visual signs of Dentinal involvement)  4=Underlying dark shadow from dentine  5=Distinct cavity with visible dentine  6=Extensive distinct cavity with visible dentine | | | |
| **25.** | **UniViss** (Kuehnisch et al. 2009, 2011)  Buecher et al. (2015) | Sound | First visible signs  Established lesion  Microcavity  Dentin exposure  Large cavity | | | |
| *DEJ-dentin-enamel junction; *pulpal involvement; Downer criteria -originally used for approximal surfaces; () cavity* | | | | | | |

## Table S3 Bitewing radiography diagnostics criteria for caries detection on occlusal surfaces

| **No.** | **Criteria** | **No caries** | **Enamel caries** | | **Dentine caries** | | | |  |
| --- | --- | --- | --- | --- | --- | --- | --- | --- | --- |
|  |  |  | **Outer half** | **Inner half** | **Outer half** | | **Inner half** | |  |
| **1.** | Marthaler et al. (1966)  5 point anatomical scale  Manton 2007 | 0  No caries | 1  Caries restricted to the enamel | 2  Caries reaching but not crossing the DEJ | 3  Caries in dentine < half way to pulp | | 4  Caries in dentine > half way to pulp | |  |
| **2.** | Downer et al. (1975) | R0  No radiolucency | R1/2  Radiolucent area confined to enamel, and not involving the DEJ | | R3  Radiolucent area involving enamel and reaching the DEJ or beyond, up to half the way through dentin | | R4  Radiolucent area involving enamel and more than half of the way through dentin | |  |
| **3.** | Pitts et al. (1984)  Russel and Pitts et al. (1993) | R0  No radiolucency | R1  Radiolucent area confined to outer half of enamel lesion | R2  Radiolucent area confined to enamel, including lesion extending up but not beyond to DEJ | R3  Radiolucent area involving enamel and reaching the DEJ or beyond, up to half the way through dentin | | R4*  Radiolucent area involving enamel and more than half of the way through dentin | |  |
|  |  |  |  |  |  |  | **with or without apparent pulpal involvement* | |  |
| **4.** | Wenzel et al. (1991)  Lussi (2003) | - | No caries in dentin (1)  Radiolucency restricted to enamel | | Dentinal caries (2)  Radiolucency involving dentin | | | |  |
| **5.** | 5 point- confidence rating scale  (Wenzel et al. 1992) | Score 1 = Caries definitely absent  Score 2 = Caries probably absent  Score 3 = Unsure if present or absent  Score 4 = Caries probably present  Score 5 = Caries definitely present | | | | | | |  |
| **6.** | Wenzel and Fejerskov (1992) | 1  No caries or changes confined in enamel | | | 2  Caries reaching dentine outer half | | 3  Deep dentinal caries half way or more to the pulp | |  |
| **7.** | In vivo: Verdonshot et al. (1992) ;In vitro: Lussi et al. (1993) | 0/1  No caries or caries restricted to the enamel | | | 2/3  Caries reaching into DEJ or extending into the dentine | | | |  |
| **8.** | Espelid et al. (1994)  Reis et al. (2004)  Rando-Mairelles et al. (2011) | 0=No radiolucency | 1=Radiolucency visible in the enamel | | 2=Radiolucency visible in the dentine but restricted to the outer 1/3 of the dentine | 3=Radiolucency extending to the middle 1/3 of the dentine | | 4=Radiolucency in the pulpal 1/3 of the dentine | |
| **9.** | **In vivo**: Ricketts et al. (1995b) | Sound | | | Radiolucency confined outer third of dentine | Radiolucency confined middle third of dentine | | Radiolucency in the pulpal third of dentine | |
| *DEJ-dentin-enamel junction; *pulpal involvement; Downer criteria -originally used for approximal surfaces; () cavity* | | | | | | | | |  |

## Table S4 Digital bitewing radiography diagnostics criteria for caries detection on occlusal surfaces

| **No.** | **Criteria** | **No caries** | **Enamel caries** | | | **Dentine caries** | | | | | | |  |
| --- | --- | --- | --- | --- | --- | --- | --- | --- | --- | --- | --- | --- | --- |
|  |  |  | **Outer half** | **Inner half** | | **Outer half** | | | | **Inner half** | |  |  |
| **1.** | **In vivo:** Lussi et al. (1995); Huth et al. (2008) | 0  No radiolucency visible | 1  Radiolucency visible in enamel | | | 2  Beyond DEJ  Radiolucency visible in the outer half of dentine | | | | 3  Deep dentine caries  Radiolucency visible in the inner half of dentine | |  |  |
|  | **In vitro:** Ekstrand et al. (1997) |  |  |  |  |  |  |  |  |  |  |  |  |
| **2.** | Gray et al. (1997) | 0  No radiolucency | 1  Radiolucency confined to enamel | | | 2  Radiolucency extending into dentine | | | | | | |  |
| **3.** | Huysmans et al. (1998) | 0  Surface sound, slight staining permitted | 1  Initial caries, white or –brown discoloration,  no detectable enamel loss | 2  Enamel lesion, enamel loss but no evidence of up to, but not beyond DEJ | | 3  Dentinal lesion, appearance of dentinal involvement or cavity extending into dentine | | | | | 4*  *Suspected pulpal involvement* | |  |
| **4.** | Rocha et al. (2005) | 0  No caries | (1)  Enamel cavity | | (2)  Cavity on the DEJ | | (3)  Dentin cavity | | | | | |  |
| **5.** | Souza Zaroni et al. (2006) | 0  No radiolucency (sound) | 1  Radiolucency in enamel | | | 2  Radiolucency in dentine (<2mm) | | 3  Radiolucency in dentine (>2mm) *without pulpal involvement* | | | 4*  *Possible pulpal involvement* | | |
| **6.** | Kuehnisch et al. (2009) | 0  Sound or enamel | | | | 2  Dentinal caries | | | | | | |  |
| **7.** | Krzyzostaniak et al. (2015) | 0  Sound (no caries detected) | 1  Radiolucency in the outer half of the enamel | 2  Radiolucency in the inner half of the enamel reaching, but not crossing, the DEJ | | 3  Radiolucency in the outer third of the dentin | | | 4  Radiolucency reaching deeper into the dentin | | | |  |
| **8.** | Pourhashemi et al. (2009) | 0/1  No radiolucency was observed (intact) | 2  Radiolucency  was observed in enamel | | | 3  Radiolucency was observed in dentin | | | | | | |  |
| **9.** | Braun et al. (2016) | R0  Sound | R1  Caries radiolucency in enamel | | | R2  Caries radiolucency in the outer half of dentin | | | | R3*  Caries radiolucency in the inner half of dentin | |  |  |
| *DEJ-dentin-enamel junction; *pulpal involvement; Downer criteria -originally used for approximal surfaces; () cavity* | | | | | | | | | | | | |  |

## Table S5 Laser fluorescence criteria for caries detection on occlusal surfaces

| **No.** | **Proposed Cut-offs**  **for the laser fluorescence measurement** | **No caries** | **Enamel caries** | | **Dentin caries** | |
| --- | --- | --- | --- | --- | --- | --- |
|  |  | D0 | D1 | D2 | D3 | D4 |
|  | **Initial proposals by the manufacturer** | | | | | |
| 1. | Clinical Guidelines (KaVo 1998) | 0-4 | 5-10 | | 11-20 | >20 |
| 2. | Clinical Guidelines (KaVo 1998) | 0-4 | 5-10 | | 11-24 | >24 or 30 |
| 3. | Clinical Guidelines (KaVo 1999&2002) | 0-4 | 5-25 | | 26-34 | >34 |
| 4. | Clinical Guidelines (KaVo 2001) | 0-9 | 10-17 | | >17 | |
|  | **Proposals from in vitro studies** | | | | | |
| 5. | Lussi et al. (1999) | 0-4 | | 4.1-10 | 10.1-18 | >18 |
| 6. | Joshi et al. (1999) | 0 | 1-25 | 25-45 | >45 | |
| 7. | Ando et al. (2000) | 0-8 | 9-15 | 16-30 | 31-50 | 51-99 |
| 8. | Shi et al. (2000) | 0-7 | 8-21 | | >21 | |
| 9. | Pereira et al. (2001) | - | >10 | | >10 | |
| 10. | Attrill et al. (2001) | 0-9 | 10-17 | | 18-99 | |
| 11. | Lussi et al. (2003) | 0-4 | | 5-12 | >12 | |
| 12. | Chong et al. (2003) | <5 | 5-25 | | 26-35 | |
| 13. | Ouellet et al. (2002) | 0-7 | 8-15 | 16-23 | 24-31 | >32 |
| 14. | Cortes et al. (2003) |  | >17 | | >40 | |
| 15. | Burin et al. (2005) | < 11 | | 12-16 | >16 | |
| 16. | Angnes et al. (2005) | <15 | | 15-19 | | >19 |
| 17. | Reis et al. (2006) | 0-13 | | 14-19 | >20 | |
| 18. | Souza Zaroni et al. (2006) | 0-5 | 6-10 | 11-20 | 21-26 | >27 |
| 19. | a.Lussi and Hellwig (2006) old DD tip A | 0-7 | 7.1-14 | 14.1-24 | >24 | |
|  | b.Lussi and Hellwig (2006) new DD cylindrical tip | 0-6 | 6.1-13 | 13.1-17 | >17 | |
|  | c.Lussi and Hellwig (2006) new DD cylindrical tip | 0-7 | 7.1-12 | 12.1-19 | >19 | |
| 20. | Sridhar et al. (2009) | 0-5 | 6-14 | 15-20 | 21-99 | |
| 21. | Seremidi et al. (2012) | <9 | 9-24 | 25-44 | >44 | |
| 22. | Achileos et al. (2013) | 0-13 | 14-20 | 21-29 | >30 | |
| 23. | Ozturk et al. (2015) | 0-12 | 13-24 | | >24 | |
| **No.** | **Proposed Cut-offs**  **for the laser fluorescence measurement** | **No caries** | **Enamel caries** | | **Dentin caries** | |
|  |  | D0 | D1 | D2 | D3 | D4 |
|  | **Proposals from in vivo studies** | | | | | |
| 24. | Lussi et al. (1999) | 0-15 | 16-25 | | >25 | |
| 25. | Verdonschot et al. (1999) | - | >19 | | >19 | |
| 26. | Lussi and (2000) Clinical guideline | 0-14 | 15-20 | | >20 | |
| 27. | Lussi et al. (2001) | 0-13 | 14-20 | | >20 | |
| 28. | Lussi et al. (2005) | 0-15 | 16-17 | | 18-31 | >31 |
| 29. | Diniz et al. (2009) | 0-14 | 15-21 | | 22-37 | >38 |
| 30. | Costa et al. (2008) | 0-20 | 21-29 | | >30 | |
| 31. | Rechmann et al. (2012) | 0-10 | 11-20 | | 21-99 | |

## Table S6 FOTI criteria for caries detection on occlusal surfaces

| **No.** | **Criteria** | **No caries** | **Enamel caries** | | | | **Dentine caries** | |
| --- | --- | --- | --- | --- | --- | --- | --- | --- |
|  |  |  | **Outer half** | | **Inner half** | | **Outer half** | **Inner half** |
| **1.** | Verdonshoot et al. (1992) ^in vivo^ | **0/1**  No caries or caries restricted to the enamel | | | | | **2**  Caries reaching into DEJ or extending into the dentine | |
| **2.** | 5-point confidence rating scale  (Wenzel et al. 1992) |  | | | | | 1= Dentinal caries almost definitely not present,  2= Dentinal caries probably not present,  3= Unsure whether Dentinal caries is present  4= Dentinal caries probably present,  5= Dentinal caries almost definitely present | |
| **3.** | Ashley et al. (1998) | - | If, on transillumination, there was a grey or yellow area expanding into enamel but not into dentine | | | | If a shadow extended into dentine | |
| **4.** | Cortes et al. (2000) | No shadow or stained area | Thin grey shadow appears when transilluminated | | Wide grey shadow appears when transilluminated | | Orange brown shadow appearing to be in dentine <2 mm in diameter | Orange brown shadow appearing to be in dentine >2 mm in diameter |
| **5.** | Cortes et al. (2003) | No shadow or stained area | Thin stain visible at the base of the fissure. | Large brown spot lesion | Thin grey shadow into enamel when transilluminated | Wide grey shadow into enamel when transilluminated | Orange/brown or  bluish/black shadow in dentine <2 mm  in diameter in bucco-lingual direction | Orange or brown  shadow appears into dentine >2 mm in  bucco-lingual direction and/or light is blocked |
| **6.** | Grossman et al. (2002) |  | Dark spot or shadow indicated caries with a 150 W halogen light source | | | |  | |
| DEJ-dentin-enamel junction; **pulpal involvement*; Downer criteria -originally used for approximal surfaces; () cavity | | | | | | | | |

## Table S7 QLF criteria for caries detection on occlusal surfaces

| **No.** | **Criteria** | **No caries** | **Enamel caries** | | **Dentine caries** | |
| --- | --- | --- | --- | --- | --- | --- |
|  |  |  | **Outer half** | **Inner half** | **Outer half** | **Inner half** |
| **1.** | Ando et al. (2000) | No change in enamel fluorescence | Slight change in enamel fluorescence | Loss of fluorescence distinctly visible without broken enamel | Loss of fluorescence distinctly visible with enamel broken | Loss of fluorescence distinctly visible with cavitation |
| **2.** | Allamari et al. (2013) | ΔF<12 | ΔF 12 to 23 | | ΔF 24 to 32 | |
| **3.** | Bussaneli et al. (2015)^A^ | ΔF -0,5 to -10 | ΔF -10,5 to -35 | | ΔF-35,5 to -45 | |
| **4.** | Gomez et al. (2013) | QLF Inspektor  ΔF < 0.08 | 0.08 <ΔF < 0.15 | | ΔF > 0.15 | |
|  |  | QLF-Custom  ΔF < 0.125 | 0.125 <ΔF < 0.193 | | ΔF <0.193 | |
| \| DEJ-dentin-enamel junction; **pulpal involvement*; Downer criteria -originally used for approximal surfaces; () cavity, A-modified criteria Allamari et al. (2013) \| \| --- \| | | | | | | |

# Results from the study selection process according to PRISMA

## Table S1a Results of systematic search of the literature on occlusal surfaces for in vitro validation studies on visual examination

| **Visual Examination** | **Study material** | | **Diagnostics** | **Validation methodology** | | | | **Validity** | | | | | |
| --- | --- | --- | --- | --- | --- | --- | --- | --- | --- | --- | --- | --- | --- |
| **In vitro validation studies on occlusal surfaces** | **Teeth**  **(N)** | **Molars/PM**  **(N)** | **Visual criteria^1^** | **Hard tissue processing** | **Caries staining** | **Visualisation /magnification** | **Scoring criteria^2^** | **Caries detection level** | | | **Dentin caries detection level** | | |
|  |  |  |  |  |  |  |  | **SE**  **(%)** | **SP**  **(%)** | **Az**  **(ROC)** | **SE**  **(%)** | **SP**  **(%)** | **Az**  **(ROC)** |
| Kay et al. (1988) | 30 | 30/- | nr | Slices | - | Microscopy | 3 | - | - | - | 24-100 | 23-85 | - |
| Wenzel et al. (1990) | 45 | -/- | 4 | Hemisection | - | Microscopy /Photo | 1 | - | - | - | - | - | - |
| Lussi et al. (1991) | 61 | 54/7 | 5 | Slices | Fuchsin/ALG | Microscopy /Photo | 5 | 82.5/83.3 | 65/61.8 | - | - | - | - |
| Nytun et al. (1992) | 30 | 30/- | 6 | Slices | - | Microscopy | 4 | - | - | - | 72 | 41 | - |
| Wenzel et al. (1992) | 81 | 81/- | 8 | Slices | - | Microscopy | 7 | - | - | 0.76 | - | - | 0.84 |
| Wenzel and Fejerskov (1992) | 78 | 78/- | 9 | Slices | - | Microscopy | 6 | - | - | - | 54 | 81 | - |
| Ketley and Holt (1993) | 100 | 100/- | 10 | Slices | - | Visually only | 2 | - | - | - | 31 | 98 | - |
| Lussi et al. (1993) | 63 | 52/11 | 6 | Slices/Ground | 4 Dyes^S^ | Microscopy /Photo | 3 | - | - | - | 12 | 93 | - |
| Verdonshoot et al. (1993) | 81 | 81/- | 8 | Slices | - | Microscopy | 3 | - | - | - | 48 | 89 | 0.76 |
| Tveit et al. (1994) | 131 | 94/37 | 11 | Grounding | - | nr | 8 | 100 | 17 | - | 44 | 97 | - |
| Wenzel et al. (1994) | 60 | 60/- | nr | Slices | - | Micro/Radiography | 6 | - | - | - | - | - | - |
| Deery et al. (1995) | 112 | 112/- | 3 | Hemisection | - | Microscopy | 1 | 77 | 73 | - | 56 | 92 | - |
| Ricketts et al. (1995a) | 48 | 48/- | 12 | Hemisection | - | Visually only | 9 | - | - | - | 48.7 | 89.6 | - |
| Gray and Paterson et al. (1997) | 35 | 35/- | 6 | Slices | - | Microscopy | 4 | - | - | - | 82 | 75 | - |
| Ekstrand et al. (1997) | 100 | 80/20 | 14 | Hemisection | - | Microscopy /Photo | 10 | - | - | - | 92-97^E^ | 85-93^E^ | - |
| Ashley et al. (1998) | 103 | 35/68 | 2 | Slices | - | Microscopy | 2 | 60 | 73 | - | 24 | 97 | - |
| Ferreira-Zandona et al. (1998) | 150 | -/150 | 15 | Slices | Rhodamine B | Microscopy/Photo | 11 | 1 | 100 | - | - | - | - |
| Huysmans et al. (1998) | 107 | - | 3 | Slices | - | Microscopy | 1 | 27 | 100 | 0.77 | - | - | - |
| Ando et al. (2000) ^E^ | 100 | 100/- | 14 | Slices | Rhodamine B | Microscopy/Photo | 10 | - | - | - | 90^E^ | 55.2^E^ | - |
| Cortes et al. (2000) | 59 | 59/- | 17 | Slices | - | Microscopy /Photo | 12 | - | - | - | 42 | 98 | 0.83 |
| El-Housseiny et al. (2001) | 46 | - | 6 | Hemisection | - | Microscopy/Photo | 4 | 69.8/72.9 | 48.6/51.4 | - | - | - | - |
| El-Housseiny et al. (2001) | 46 | - | 6^P^ | Hemisection | - | Microscopy/Photo | 4 | 58/63 | 66/85 | - | - | - | - |
| Pereira et al. (2001) | 101 | 101/- | 3 | Slices/Polish | Red 52^S^ | MicroscopyPhoto | 1 | 92 | 54 | - | 17/7 | 97/99 | 0.78/0.77 |
| Alwas-Danowska et al. (2002) | 49 | 49/- | 17 | Hemisection | - | Microscopy/Photo | nr | - | - | - | 40-60 | 88-94 | 0.83-0.90 |
| Costa et al. (2002) | 50 | 25/25 | 6 | Slices | - | Microscopy | 4 | 18.7 | 100 | - | 14.3 | 100 | - |
| Grosman et al. (2002) | 214 | - | 14 | Slices | - | Microscopy | 13 | - | - | - | 48/50 | 93/92 | - |
| Tonioli et al. (2002) | 29 | 29/- | 18 | Slices | - | Microscopy/Photo | 4 | - | - | 0.68 | - | - | - |
| Cortes et al. (2003) | 111 | 111/- | 14 | Slices | - | Microscopy | 14 | 98 | 38 | 0.87 | 55 | 90 | 0.83 |
| Forgie et al. (2003) | 80 | 40/40 | 20 | Slices | - | Microscopy | 16 | 43 | 91.7 | - | 26.8 | 96.6 | - |
| nr-not reported; ^1^See Table 1-Visual diagnostics criteria; ^2^See Table Semiquantitative histological criteria; ^S^Staining (methyl metha-acrilate-MMA, Rhodamine B, Basic Fuchsin, Acetyl Light Green-ALG: 4 Dyes); Red 52- ; To Dye For- glycol based Caries indicator dye ^P^Use Probe for visual diagnostic; ^E^Ekstrand criteria | | | | | | | | | | | | | |

| **Visual Examination** | **Study material** | | **Diagnostics** | **Validation methodology** | | | | **Validity** | | | | | |
| --- | --- | --- | --- | --- | --- | --- | --- | --- | --- | --- | --- | --- | --- |
| **In vitro validation studies on occlusal surfaces** | **Teeth**  **(N)** | **Molars/PM**  **(N)** | **Visual criteria^1^** | **Hard tissue processing** | **Caries Staining** | **Visualisation /magnification** | **Scoring criteria^2^** | **Caries detection level** | | | **Dentin caries detection level** | | |
|  |  |  |  |  |  |  |  | **SE**  **(%)** | **SP**  **(%)** | **Az**  **(ROC)** | **SE**  **(%)** | **SP**  **(%)** | **Az**  **(ROC)** |
| Kordic et al. (2003) | 61 | 36/25 | 1 | Hemisec/Poli | - | Microscopy | 1 | 100 | 37.5 | 0.76 | 81.8 | 68 | 0.75 |
| Lussi et al. (2003) | 95 | -/- | 1 | Grounding | 4 Dyes | Photography | 1 | - | - | - | 98 | 35 | - |
| Fung et al. (2004) | 25 | 25/- | 21 | Slices | - | Microscopy | 3 | - | - | - | 64-80^E^ | 85-88^E^ | - |
| Reis et al. (2004) | 45 | - | 14 | Slices | - | Microscopy | 10 | - | - | - | 80-100^E^ | 25-37^E^ | - |
| Angnes et al. (2005) ^E^ | 57 | 57/- | 14 | Slices | - | Microscopy | 10 | - | - | - | 68.7/69^E^ | 85.1/91^E^ | - |
| Burin et al. (2005) ^E^ | 54 | 54/- | 14 | Hemisection | - | Microscopy | 10 | - | - | - | 72-83^E^ | 76-81^E^ | 0.78-0.84^E^ |
| Erten et al. (2005) ^E^ | 84 | 84/- | 14 | Hemisection | - | Microscopy | 10 | 26 | 87 | - | - | - | - |
| Mestriner et al. (2005) | 38 | 38/- | 22 | Slices | - | Microscopy/Photo | 1 | - | - | - | 25/17 | 96/95 | - |
| Deery et al. (2006) | 37 | 25/12 | 3 | Slices | - | Microscopy | 2 | 77/78 | 73/87 | - | 56/68 | 92/94 | - |
| Erten et al. (2006) | 68 | 68/- | 23 | Hemisection | - | Microscopy | 4 | - | - | - | - | - | - |
| Reis et al. (2006) ^E^ | 57 | 57/- | 14 | Slices | - | Microscopy | 10 | - | - | - | 69^E^ | 88^E^ | - |
| Reis et al. (2006) | 57 | 57/- | 14 | Slices | - | Microscopy | 10 | 75 | 55 | - | - | - | - |
| Souza-Zaroni et al. (2006) | 47 | 47/- | 18 | Slices/Ground | - | Microscopy | 16 | 42-61 | 69-93 | - | - | - | - |
| Ekstrand et al. (2007) | 141 | - | 24 | Slices | - | Microscopy | 10 | - | - | - | - | - | - |
| Manton et al. (2007) | 67 | 67/- | 14 | Slices | - | Microscopy | 14 | - | - | - | 68 | 71 | - |
| Jablonski-Momeni et al. (2008) | 100 | 85/15 | 24 | Slices | - | Microscopy | 2,10 | 59-73 | 74-91 | 0.73-0.86 | 48-83 | 82-94 | 0.87-0.88 |
| Rodrigues et al. (2008) | 119 | 119/- | 24 | - | Rhodamine B | Microscopy/Photo | 1 | - | - | - | 73 | 65 | 0.75 |
| Valera FB et al. (2008) ^E^ | 72 | 54/18 | 14 | Slices/Ground | - | Microscopy/Photo | 10 | 44 | 100 | 0.74 | - | - | - |
| Diniz and Rodrigues (2009) ^E^ | 163 | 163/- | 18 | Grounding | Rhodamine B | Microscopy | 10 | - | - | - | 100^E^ | 69^E^ | 0.54^E^ |
| Diniz and Rodrigues (2009) | 163 | 163/- | 24 | Grounding | Rhodamine B | Microscopy | 1 | - | - | - | 75 | 62 | 0.73 |
| Kuehnisch et al. (2009) | 54 | 54/- | 2 | Slices | - | Microscopy/MR | 2 | - | - | - | 68 | 93.1 | 0.69/0.87 |
| Pereira et al. (2009) | 96 | 96/- | 14 | Slices | - | Microscopy | 1 | 77 | 73 | 0.78 | - | - | - |
| Pourhashemi et al. (2009) | 80 | -/80 | 4 | Hemisection | - | Microscopy | 11 | - | - | - | 43.8 | 78.3 | - |
| Rodrigues et al. (2009) | 179 | - | 2 | Hemisection | - | Microscopy | 6 | 97 | 40 | 0.75 | 30 | 98 | 0.75 |
| Sridhar et al. (2009) ^E^ | 50 | 17/33 | 14 | Hemisection | MMA | Microscopy | 10 | - | - | - | - | - | - |
| De Paula et al. (2011) | 26 | 26/- | 6 | Hemisection | - | Microcopy | 4 | 63 | 100 | 0.81 | - | - | - |
| Diniz et al. (2011) ^E^ | 104 | - | 24 | Hemisection | - | Microscopy | 10 | - | - | - | 83^E^ | 79^E^ | 0.85^E^ |
| Jablonski-Momeni et al. (2011) | 100 | 85/15 | 24 | Slices | - | Microscopy | 2 | 91 | 54 | 0.82 | 70 | 91 | 0.87 |
| Boye et al. (2012) | 50 | 32/18 | nr | Slices | - | Microscopy/Photo | 12 | - | - | - | 65.5 | 82.4 | - |
| Mitropoulos et al. (2012) ^E^ | 38 | - | 24 | Slices | - | Microscopy/Photo | 10 | 84/81 | 66/68 | 0.75/0.80 | - | - | - |
| Seremidi et al. (2012) | 41 | -/41 | 14 | Grounding | - | Microscopy/Photo | 2 | 73 | 63.6 | 0.68 | 75 | 91.9 | 0.83 |
| Achilleos et al. (2013) ^E^ | 38 | 12/26 | 24 | Slices | - | Photography | 10 | 80/86 | 50/50 | 0.43/0.40 | - | - | - |
| nr-not reported; 1See Table Visual diagnostics criteria; ^2^See Table Semiquantitative histological criteria; ^S^Staining (methyl metha-acrilate-MMA, Rhodamine B, Basic Fuchsin, Acetyl Light Green-ALG: 4 Dyes); Red 52- ; To Dye For- glycol based Caries indicator dye ^P^Use Probe for visual diagnostic; ^E^Ekstrand criteria | | | | | | | | | | | | | |

| **Visual Examination** | **Study material** | | **Diagnostics** | **Validation methodology** | | | | **Validity** | | | | | |
| --- | --- | --- | --- | --- | --- | --- | --- | --- | --- | --- | --- | --- | --- |
| **In vitro validation studies on occlusal surfaces** | **Teeth**  **(N)** | **Molars/PM**  **(N)** | **Visual criteria^1^** | **Hard tissue processing** | **Caries Staining** | **Visualisation /magnification** | **Scoring criteria^2^** | **Caries detection level** | | | **Dentin caries detection level** | | |
|  |  |  |  |  |  |  |  | **SE**  **(%)** | **SP**  **(%)** | **Az**  **(ROC)** | **SE**  **(%)** | **SP**  **(%)** | **Az**  **(ROC)** |
|  |  |  |  |  |  |  |  |  |  |  |  |  |  |
| Gomez et al. (2013) | 112 | - | 24 | Hemisection | - | Photography | 14 | 90 | 87 | 0.98 | 87 | 93 | 0.95 |
| Van Hilsen et al. (2013) | 45 | - | 24 | Slices | To Dye For^S^ | Photography | 4 | 80.5 | 52.5 | - | 48 | 57-8 | - |
| Arslan et al. (2014) | 60 | - | 24 | Slices | - | Microscopy /M-CT | 2 | - | - | - | - | - | - |
| Patel et al. (2014) | 264 | - | 24 | Slices | - | Microscopy/Photo | 2 | 92 | 43 | 0.84 | - | - | - |
| Sisodia et al. (2014) ^E^ | 60 | - | 24 | Hemisection | - | Microscopy/Photo | 10 | 67-71^E^ | 72-73^E^ | 0.68-0.72^E^ | - | - | - |
| Alomari et al. (2015) | 160 | -/- | nr | Slices | - | Microscopy | 2 | - | - | - | 82 | 49 | 0.65 |
| Buecher et al. (2015) | 196 | 196/- | 25 | - | - | M-CT | 1 | 86 | 91 | 0.82 | 87 | 100 | 0.89 |
| Jallad et al. (2015) ^E^ | 60 | 30/30 | 24 | Slices | Rhodamine B | Microscopy/Photo | 10 | 82^E^ | 86^E^ | 0.87^E^ | - | - | - |
| Neuhaus et al. (2015) | 100 | 91/9 | 24 | Slices | Rhodamine B | Microscopy/Photo | 1 | 74-85 | 10-58 | 0.48-0.66 | 47-66 | 76-84 | 0.66-0.71 |
| Özkan et al. (2015) | 60 | -/- | 24 | Slices | - | Microscopy/M-CT | 2 | 94/94 | 55/36 | 0.84/0.78 | 74/74 | 97/100 | 0.89/0.86 |
| Ozturk et al. (2015) | 44 | 44/- | 24 | Slices | - | Microscopy/Photo | 17 | - | - | - | 33-76 | 90-100 | 0.67-0.84 |
| Quidemat et al. (2015) | 142 | 142/- | 24 | Slices | - | Microscopy | 2 | - | - | - | 73 | 95 | 0.84 |
| Braun et al. (2016) | 84 | 69/15 | 24 | Slices | 4 Dyes | Microscopy | 1 | 99 | 53 | - | 85 | 88 | - |
| Iranzo-Cortez et al. (2017) | 64 | 37/28 | 24 | Slices |  | Microscopy | 18 | 82 | 100 | 0.87/0.91 | - | - | - |
| nr-not reported; ^1^See Table Visual diagnostics criteria; ^2^See Table Semiquantitative histological criteria; ^S^Staining (methyl metha-acrilate-MMA, Rhodamine B, Basic Fuchsin, Acetyl Light Green-ALG: 4 Dyes); Red 52- ; To Dye For- glycol based Caries indicator dye ^P^Use Probe for visual diagnostic; ^E^Ekstrand criteria | | | | | | | | | | | | | |

## Table S1b Results of systematic search of the literature on occlusal surfaces for in vivo validation studies on visual examination

| **Visual Examination** | **Study material** | | | | **Diagnostics** | **Validation methodology** | | | **Validity** | | | | | |
| --- | --- | --- | --- | --- | --- | --- | --- | --- | --- | --- | --- | --- | --- | --- |
| **In vivo validation studies on occlusal surfaces** | **Patients**  **(N)** | **Age**  **(years)** | **Teeth (N)** | **Molars/PM (N)** | **Visual Criteria** | **Histology technique** | **Validation** | **Reference method** | **Caries detection level** | | | **Dentin caries detection level** | | |
|  |  |  |  |  |  |  |  |  | **SE**  **(%)** | **SP**  **(%)** | **Az**  **(ROC)** | **SE**  **(%)** | **SP**  **(%)** | **Az**  **(ROC)** |
| Verdonshoot et al. (1992) | 13 | 7-13 | 23 | 19/4 | 7 | - | Visually | 7 | - | - | - | 13 | 94 | - |
| Ie et al. (1995) | 50 | 5-15 | 60 | 60/0 | 17 | Cav.prep. | Visually | 7 | - | - | - | 4 | 97 | 0.66 |
| Ricketts et al. (1995b) | 20 | nr | 40 | 39/1 | nr | Cav.prep. | Car det.dye | nr | 27 | 89 | - | 3 | 97 | - |
| Nyvad et al. (1999) | 50 | 9-14 | nr | nr | 16 | - | Visually | 16 | - | - | - | - | - | - |
| Lussi et al. (2001) | 240 | 8-20 | 332 | 246/86 | 2 | Cav.prep. | Visually | 2 | 62 | - | - | 31 | - | - |
| Sheehy et al. (2001) | 170 | 4-8 | 170 | 170/- | 14 | - | Visually | 14 | - | - | - | - | - | - |
| Heinrich-Weltzien et al. (2002) | 97 | 17-21 | 248 | 248/- | 14 | Cav.prep. | Visually | 2 | - | - | - | 25 | 100 | - |
| Anttonen et al. (2003) | 109 | 7-14 | 613 | 613/- | 19 | Drilling | Visually | 19 | - | - | - | 92 | 69 | - |
| Astvaldsdotttir et al. (2004) | nr | 18-30 | 34 | nr | 14 | Cav Prep. | Visual/Photo | 2 | - | - | - | - | - | - |
| Traneus et al. (2004) | 30 | 18-42 | 52 | 52/- | 14 | Cav.prep. | Visual/Photo | 6 | - | - | - | - | - | - |
| Wolwacz et al. (2004) | 23 | 12-16 | 147^sites^ | 12 | 14 | - | Visually | nr | - | - | - | - | - | - |
| Angnes et al. (2005) ^E^ | 38 | 19-35 | 57 | 57/- | 17 | Hist.prep. | In vitro | 10 | - | - | - | 75/69^E^ | 84/81^E^ | 0.82/0.86^E^ |
| Akarsu et al. (2006) | 161 | 18-25 | 165 | 165/- | 14 | Cav.prep. | Visually | 6 | 89/71 | 91/89 | - | 96/98 | 62/60 | - |
| Olmez et al. (2006) | 28 | 7-17 | 92 | 77/15 | 14 | Cav.prep. | Visually | 2 | - | - | - | 69 | 100 | - |
| Reis et al. (2006) ^E^ | 38 | 19-30 | 57 | 57/- | 14 | Hist.prep. | In vitro | 10 | 75 | 58 | - | 72^E^ | 84^E^ | - |
| Costa et al. (2008) | 26 | 10-13 | 151 | nr | 10 | Cav.Prep | Visually* | 6 | - | - | - | 50 | 95 | - |
| Huth et al. (2008) | 120 | 6-51 | 120 | 120/- | 25 | Cav.prep. | Visually | 2 | - | - | - | - | - | - |
| Diniz et al. (2009) ^E^ | 35 | 7-12 | 130 | 130/- | 12 | Hist.prep. | In vitro | 10 | - | - | - | - | - | - |
| Diniz et al. (2009) | 35 | 7-12 | 130 | 130/- | 12 | Hist.prep. | In vitro | 1 | - | - | - | - | - | - |
| Rando-Meirelles et al. (2011) | 327 | 12-15 | 789 | 789/- | nr | - | Radiography | 8 | - | - | - | - | - | - |
| Diniz et al. (2012) | 88 | 18-35 | 105 | 65/40 | 24 | Hist.Prep. | In vitro | 1 | 93 | 60 | 0.86 | 52 | 77 | 0.75 |
| Rechmann et al. (2012) | 100 | 13-58 | 433 | 343/90 | 24 | - | Visually | nr | - | - | - | - | - | - |
| Alammari et al. (2013) | 46 | 18-75 | 46 | nr | 24 | Cav.Prep | Visual/Photo | nr | - | - | - | - | - | - |
| Bozdemir et al. (2013) | 37 | nr | 156 | 142/14 | 14 | Cav.prep. | Visually | 2 | 97-98 | 90-93 | 0.92-0.95 | 97-98 | 49-49 | 0.56-0.57 |
| Sinanoglu et al. (2014) | 37 | 13-nr | 217 | nr | 24 | Cav.prep. | Visually | 2 | - | - | 0.54-0.77 | - | - | - |
| Bahrololoomi et al. (2015) | 31 | 7-13 | 109 | 109/- | 14 | Cav.prep. | Visually | 6 | 80/81 | 86/100 | - | 63/55 | 97/97 | - |
| Cotta et al. (2015) ^E^ | 42 | 16-39 | 49 | 49/- | 24 | Hist.prep. | In vitro | 10 | 71 | 44 | 0.57 | 71^E^ | 86^E^ | 0.79 |
| Castilho et al. (2016) ^E^ | 26 | 16-39 | 43 | 43/- | 24 | Hist.prep. | In vitro | 10 | 50 | 71 | 0.60 | 82^E^ | 100^E^ | 0.91 |
| Melo et al. (2017) | 152 | 14-39 | 302 | 148/154 | 24 | Cav.prep. | Visually | 24 | - | - | - | 79 | 72.3 | 0.75 |
| nr-not reported; *Enameloplasty-fissure eradiation;  ^E^Ekstrand criteria | | | | | | | | | | | | | | |

## Table S2a Results of systematic search of the literature on occlusal surfaces for in vitro validation studies on conventional radiography

| **Conventional bitewing radiography examination** | **Study material** | | **Diagnostics** | **Validation methodology** | | | | **Validity** | | | | | |
| --- | --- | --- | --- | --- | --- | --- | --- | --- | --- | --- | --- | --- | --- |
| **In vitro validation studies on occlusal surfaces** | **Teeth**  **(N)** | **Molars/PM**  **(N)** | **Radiographic Criteria^1^** | **Hard tissue processing** | **Caries Staining** | **Visualisation /magnification** | **Scoring criteria^2^** | **Caries detection level** | | | **Dentin caries detection level** | | |
|  |  |  |  |  |  |  |  | **SE**  **(%)** | **SP**  **(%)** | **Az**  **(ROC)** | **SE**  **(%)** | **SP**  **(%)** | **Az**  **(ROC)** |
| Wenzel et al. (1990) | 45 | -/- | 1 | Hemisection | - | Microscopy/Photo | 1 | - | - | - | 85-100 | 44-92 | - |
| Wenzel et al. (1991) | 81 | 81/- | 4 | Slices | - | Microscopy | 3 | - | - | - | - | - | - |
| Wenzel et al. (1992) | 81 | 81/- | 5 | Slices | - | Microscopy | 7 | - | - | 0.72 | - | - | 0.89 |
| Wenzel and Fejerskov (1992) | 78 | 78/- | 6 | Slices | - | Microscopy | 6 | - | - | - | 48 | 81 | - |
| Nytun et al. (1992) | 30 | 30/- | 7 | Slices | - | Microscopy | 5 | - | - | - | 66 | 50 | - |
| Ketley and Holt (1993) | 100 | 100/- | 2 | Slices | - | Visually only | 2 | - | - | - | 67 | 92 | - |
| Lussi et al. (1993) | 63 | 52/11 | 7 | Slices/Ground | 4 Dyes^s^ | Microscopy/Photo | 3 | - | - | - | 45 | 83 | - |
| Russel and Pitts (1993) ^D-speed^ | 120 | -/- | 1 | Hemisection | - | Microscopy | 1 | 12 | 95 | - | 181 | 98 | - |
| Russel and Pitts (1993) ^E-speed^ | 120 | -/- | 1 | Hemisection | - | Microscopy | 1 | 12 | 96 | - | 21 | 99 | - |
| Verdonshoot et al. (1993) | 81 | 81/- | 5 | Slices | - | Microscopy | 3 | - | - | - | 61 | 79 | 0.72 |
| Espelid et al. (1994) | 82 | 45/37 | 8 | Drilling | - | Visually only | 6 | - | - | - | 90 | 76 | 0.90 |
| Hintze et al. (1994) | 65 | -/- | 5 | Slices | - | Microscopy | 3 | - | - | - | - | - | 0.77/0.77 |
| Wenzel et al. (1994) | 60 | 60/- | nr | Slices | - | Micro/Radiography | 6 | - | - | - | - | - | - |
| Lazarchik et al. (1995) | 100 | -/- | 5 | Slices/Ground | 4 Dyes | Microscopy/Photo | 4 | - | - | - | 58-69 | 57-79 | - |
| Lussi et al. (1995) | 26 | 26/- | 6 | Slices/Ground | 4 Dyes | Microscopy/Photo | 3 | - | - | - | 62 | 77 | - |
| Ricketts et al. (1995) | 48 | 48/- | nr | Hemisection | Caries Det Dye | Visually only | 9 | - | - | - | 62.2 | 76.1 | - |
| Hintze et al. (1996) | 103 | -/- | 5 | Slices | - | Microscopy | 1 | - | - | - | - | - | 0.76-0.80 |
| Gray and Paterson (1997) | 35 | 35/- | 11 | Slices | - | Microscopy | 6 | - | - | - | 79 | 70 | - |
| Ricketts et al. (1997) | 96 | 48/48 | 1 | Slices | - | Radiography | 1 | 18-28 | 80-97 | - | 18-30 | 83-91 | - |
| Schneiderman et al. (1997) | 25 | 14/12 | nr | Slices | SVG | Microscopy | nr | 18/20 | 98/100 | - | - | - | - |
| Ekstrand et al. (1997) | 100 | 80/20 | 11 | Hemisec/Slic | - | Microscopy/Photo | 10 | - | - | - | 51-54 | 100 | - |
| Ashley et al. (1998) | 103 | 35/68 | 2 | Slices | - | Microscopy | 2 | 19 | 80 | - | 24 | 89 | - |
| Huysmans et al. (1998) | 107 | - | 12 | Slices | - | Microscopy | 1 | 58 | 87 | 0.84 |  |  |  |
| nr-not reported;  ^1^See Table Radiographic diagnostics criteria; ^2^See Table semiquantitative histological criteria;  ^S^Staining (methyl metha-acrilate-MMA, Rhodamine B, Basic Fuchsin, Acetyl Light Green-ALG: 4 Dyes)  ^E^Ekstrand criteria | | | | | | | | | | | | | |

| **Conventional bitewing radiography examination** | **Study material** | | **Diagnostics** | **Validation methodology** | | | | **Validity** | | | | | |
| --- | --- | --- | --- | --- | --- | --- | --- | --- | --- | --- | --- | --- | --- |
| **In vitro validation studies on occlusal surfaces** | **Teeth**  **(N)** | **Molars/PM**  **(N)** | **Radiographic Criteria^1^** | **Hard tissue processing** | **Caries Staining** | **Visualisation /magnification** | **Scoring criteria^2^** | **Caries detection level** | | | **Dentin caries detection level** | | |
|  |  |  |  |  |  |  |  | **SE**  **(%)** | **SP**  **(%)** | **Az**  **(ROC)** | **SE**  **(%)** | **SP**  **(%)** | **Az**  **(ROC)** |
| Abreu Jr et al. (1999) | 40 | 20/20 | 5 | Slices | - | Microscopy | 12 | - | - | - | - | - | 0.77 |
| Cortes et al. (2000) | 59 | 59/- | 8 | Slices | - | Microscopy/Photo | 12 | - | - | - | 32 | 100 | 0.87 |
| Shi et al. (2000) | 76 | 48/28 | 5 | Slices | - | Micro Radiography | 1 | - | - | 0.51 | - | - | 0.80 |
| Costa et al. (2002) | 50 | 25/25 | 7 | Slices | - | Microscopy | 4 | 0 | 100 | - | 42.8 | 73.7 | - |
| Grossman et al. (2002) | 214 | - | 11 | Slices | - | Microscopy | 13 | - | - | - | 38 | 93 | - |
| Hintze et al. (2002) | 177 | 146/31 | 5 | Slices | - | Microscopy | 7 | - | - | - | - | - | 0.82/0.81 |
| Lussi et al. (2003) | 95 | -/- | 4 | Grounding | 4 Dyes | Photography | 1 | - | - | - | 88 | 71 | - |
| Reis et al. (2004) ^E^ | 45 | - | 11 | Slices | - | Microscopy | 10 | - | - | - | 33-60^E^ | 74-93^E^ | - |
| Burin et al. (2005) ^E^ | 54 | 54/- | 8 | Hemisection | - | Microscopy | 10 | - | - | - | 27.8^E^ | 93-95^E^ | 0.75-0.76 |
| Mestriner et al. (2005) | 38 | 38/- | 10 | Slices | - | Microscopy/Photo | 1 | - | - | - | 45 | 73 | - |
| Rocha et al. (2005) | 48 | -/- | 12 | Slices | - | Microscopy | nr | 21/57 | 100/100 | - | - | - | - |
| Souza-Zaroni et al. (2006) | 47 | 47/- | 13 | Slices/Ground | - | Microscopy | 16 | 26-56 | 74-99 | - | - | - | - |
| Manton et al. (2007) | 67 | 67/- | 1 | Slices | - | Microscopy | 14 | - | - | - | 15 | 95 | - |
| Rodrigues et al. (2008) | 119 | 119/- | 10 | Grounding | Rhodamine B | Microscopy/Photo | 1 | - | - | - | 34 | 97 | 0.71 |
| Valera FB et al. (2008) ^E^ | 72 | 54/18 | 8 | Slices/Ground | - | Microscopy/Photo | 10 | 12^E^ | 98^E^ | 0.55^E^ | - | - | - |
| Kuehnisch et al. (2009) | 54 | 54/- | 14 | Slices | - | Microscopy/MR | 2 | - | - | - | 76/64 | 79/68 | 0.63/0.50 |
| Pereiera et al. (2009) | 96 | 96/- | 1 | Slices | - | Microscopy | 11 | - | - | - | - | - | - |
| Pourhashemi et al. (2009) | 80 | -/80 | 16 | Hemisection | - | Microscopy | 11 | - | - | - | 27.3 | 43.8 | - |
| Sridhar et al. (2009) ^E^ | 50 | 17/33 | 8 | Hemisection | MMA | Microscopy | 10 | - | - | - | - | - | - |
| Diniz et al. (2011) ^E^ | 104 | - | 10 | Hemisection | - | Microscopy/Photo | 10 | - | - | - | 44^E^ | 94^E^ | 0.69^E^ |
| Arslan et al. (2014) | 60 | - | 11 | Slices | - | Microscopy/M-CT | 2 | - | - | - | - | - | - |
| Bussaneli et al. (2015) | 94 | - | 10 | Slices | - | Microscopy/Photo | 19 | - | - | - | 13.8 | 86.2 | 0.37 |
| Krzyzostaniak et al. (2015) | 135 | 68/67 | 15 | Slices | - | Microscopy | 1 | - | - | - | - | - | 0.61 |
| Özkan et al. (2015) | 60 | -/- | 10 | Slices | - | Microscopy/M-CT | 2 | 69/71 | 100/91 | 0.85/0.83 | 78/67 | 100/100 | 0.98/0.96 |
| nr-not reported;  ^1^See Table Radiographic diagnostics criteria; ^2^See Table semiquantitative histological criteria;  ^S^Staining (methyl metha-acrilate-MMA, Rhodamine B, Basic Fuchsin, Acetyl Light Green-ALG: 4 Dyes)  ^E^Ekstrand criteria | | | | | | | | | | | | | |

## Table S2b Results of systematic search of the literature on occlusal surfaces for in vivo validation studies on conventional radiography

| **Conventional bitewing radiography examination** | **Study material** | | | | **Diagnostics** | **Validation methodology** | | | **Validity** | | | | | |
| --- | --- | --- | --- | --- | --- | --- | --- | --- | --- | --- | --- | --- | --- | --- |
| **In vivo validation studies on occlusal surfaces** | **Patients**  **(N)** | **Age**  **(years)** | **Teeth (N)** | **Molars**  **/PM**  **(N)** | **Criteria** | **Histology technique** | **Validation** | **Reference method** | **Caries detection level** | | | **Dentin caries detection level** | | |
|  |  |  |  |  |  |  |  |  | **SE**  **(%)** | **SP**  **(%)** | **Az**  **(ROC)** | **SE**  **(%)** | **SP**  **(%)** | **Az**  **(ROC)** |
| Verdonshoot et al. (1992) | 13 | 7-13 | 23 | 19/4 | 7 | - | Visually | 3 | - | - | - | 58 | 66 | - |
| Lussi et al. (1995) | 22 | 10-28 | 26 | 26/0 | 10 | Hist. prep. | In vitro | 2 | - | - | - | 62 | 77 | - |
| Ricketts et al. (1995b) | 20 | nr | 40 | 39/1 | 9 | Cav. Prep | Car det.dye | nr | 6 | 100 | - | 13 | 100 | - |
| Hintze et al. (1996) | nr | nr | 130 | 130/- | 5 | Hist. prep. | In vitro | 1 | - | - | - | - | - | 0.77 |
| Townsend et al. (2000) | 53 | 6-18 | 530 | -/- | nr | - | Radiography | nr | - | - | - | - | - | - |
| Lussi et al. (2001) | 240 | 8-20 | 332 | 246/86 | nr | Cav.prep. | Visually | 2 | - | - | - | 63 | 99 | - |
| Thomas et al. (2001) | 49 | 16-18 | 299 | 299/- | 5 | - | ECM | nr | 25 | 93 | 0.65-0.71 | - | - | - |
| Heinrich-Weltzien et al. (2002) | 97 | 17-21 | 248 | 248/- | 7 | Cav.Prep | Visually | 2 | - | - | - | 70 | 96 | - |
| Astvaldsdotttir et al. (2004) | nr | 18-30 | 34 | nr | 10 | Cav Prep. | Visual/Photo | 2 | - | - | - | - | - | - |
| Traneus et al. (2004) | 30 | 18-42 | 52 | 52/- | 10 | Cav.prep. | Visual/Photo | 6 | - | - | - | - | - | - |
| Wolwacz et al. (2004) | 23 | 12-16 | 147^sites^ | nr | 11 | - | Visually | nr | - | - | - | - | - | - |
| Angnes et al. (2005) ^E^ | 38 | 19-35 | 57 | 57/- | 11 | Hist.prep. | In vitro | 10 | - | - | - | 0/6.2 | 99/97 | 0.51/0.61 |
| Akarsu et al. (2006) | 161 | 18-25 | 165 | 165/- | 8 | Cav.prep. | Visually | 6 | 65/65 | 55/61 | - | 91/91 | 72/76 | - |
| Olmez et al. (2006) | 28 | 7-17 | 92 | 77/15 | 8 | Cav.prep. | Visually | 2 | - | - | - | 36 | 100 | - |
| Costa et al. (2008) | 26 | 10-13 | 151 | nr | 7 | Cav.Prep | Visually* | 6 | - | - | - | 26 | 94 | - |
| Rando-Meirelles et al. (2011) | 327 | 12-15 | 789 | 789/- | 8 | - | Radiography | 8 | - | - | - | - | - | - |
| Diniz et al. (2012) | 88 | 18-35 | 105 | 65/40 | 10 | Hist.prep. | In vitro | 1 | 29 | 100 | 0.64 | 44 | 97 | 0.74 |
| Bahrololoomi et al. (2015) | 31 | 7-13 | 109 | 109/- | 7 | Cav.prep. | Visually | 6 | 79/81 | 100/100 | - | 84/84 | 100/100 | - |
|  |  |  |  |  |  |  |  |  |  |  |  |  |  |  |
| nr-not reported; *Enameloplasty-fissure eradiation; ^E^Ekstrand criteria | | | | | | | | | | | | | | |

##

## Table S3a Results of systematic search of the literature on occlusal surfaces for in vitro validation studies on digital radiography

| **Digital bitewing radiography examination** | **Study material** | | **Diagnostics** | **Validation methodology** | | | | | **Validity** | | | | | | | |
| --- | --- | --- | --- | --- | --- | --- | --- | --- | --- | --- | --- | --- | --- | --- | --- | --- |
| **In vitro validation studies on occlusal surfaces** | **Teeth**  **(N)** | **Molars/PM**  **(N)** | **Radiographic Criteria^1^** | **Hard tissue processing** | **Caries Staining** | **Visualisation /magnification** | **Scoring criteria^2^** | | **Caries detection level** | | | | **Dentin caries detection level** | | | |
|  |  |  |  |  |  |  |  |  | **SE**  **(%)** | **SP**  **(%)** | **Az**  **(ROC)** | | **SE**  **(%)** | | **SP**  **(%)** | **Az**  **(ROC)** |
| Wenzel et al. (1990) | 45 | -/- | 1 | Hemisection | - | Microscopy/Photo | 1 | | - | - | - | | - | | - | - |
| Wenzel et al. (1991) | 81 | 81/- | 4 | Slices | - | Microscopy | 3 | | - | - | - | | 54-89 | | 10-24 | - |
| Wenzel et al. (1992) | 81 | 81/- | 5 | Slices | - | Microscopy | 7 | | - | - | 0.69-0.70 | | - | | - | 0.82-0.88 |
| Wenzel and Fejerskov (1992) | 78 | 78/- | 6 | Slices | - | Microscopy | 6 | | - | - | - | | 54/71 | | 77/85 | - |
| Wenzel et al. (1995) | 106 | -/- | 5 | Slices | - | Microscopy | 4 | | - | - | - | | - | | - | 0.76-0.83 |
| Ashley et al. (1998) | 103 | 35/68 | 2 | Slices | - | Microscopy | 2 | | 24 | 80 | - | | 19 | | 89 | - |
| Abreu Jr et al. (1999) | 40 | 20/20 | 5 | Slices | - | Microscopy | 12 | | - | - | - | | - | | - | 0.72 |
| Costa et al. (2002) | 50 | 25/25 | 7 | Slices | - | Microscopy | 4 | | 14.3 | 64.3 | - | | 28.5 | | 64.3 | - |
| Hintze et al. (2002) | 177 | 146/31 | 5 | Slices | - | Microscopy | | 7 | - | - | - | - | | - | | 0.75-0.81 |
| Mestriner et al. (2005) | 38 | 38/- | 10 | Slices | - | Microscopy/Photo | 1 | | - | - | - | | 33 | | 80 | - |
| Rocha et al. (2005) | 48 | -/- | 12 | Slices | - | Microscopy | nr | | 23-64 | 66-100 | - | | - | | - | - |
| Kuehnisch et al. (2009) | 54 | 54/- | 11 | Slices | - | Microscopy/MR | 2 | | - | - | - | | 60 | | 85.7 | 0.62-0.67 |
| Buecher et al. (2015) | 196 | 196/- | 1 | - | - | M-CT | 1 | | - | - | - | | 59 | | 99 | 0.79 |
| Krzyzostaniak et al. (2015) | 135 | 68/67 | 15 | Slices | - | Microscopy | 1 | | - | - | - | | - | | - | 0.58 |
| Ozturk et al. (2015) | 44 | 44/- | 2 | Slices | - | Microscopy/Photo | 17 ? | | - | - | - | | 38-62 | | 100 | 0.69-0.81 |
| Braun et al. (2016) | 84 | 69/15 | 17 | Slices | 4 Dyes^s^ | Microscopy | 1 | | 42 | 95 | - | | 71 | | 98 | - |
|  |  |  |  |  |  |  |  | |  |  |  | |  | |  |  |
| nr-not reported;  ^1^See Table Radiographic diagnostics criteria; ^2^ See Table Semiquantitative histological criteria;  ^S^Staining (methyl metha-acrilate-MMA, Rhodamine B, Basic Fuchsin, Acetyl Light Green-ALG: 4 Dyes) | | | | | | | | | | | | | | | | |

## Table S3b Results of systematic search of the literature on occlusal surfaces for in vivo validation studies on digital bitewing radiography

| **Digital bitewing radiography examination** | **Study material** | | | | **Diagnostics** | **Validation methodology** | | | **Validity** | | | | | |
| --- | --- | --- | --- | --- | --- | --- | --- | --- | --- | --- | --- | --- | --- | --- |
| **In vivo validation studies on occlusal surfaces** | **Patients**  **(N)** | **Age**  **(years)** | **Teeth (N)** | **Molars**  **/PM**  **(N)** | **Criteria** | **Histology technique** | **Validation** | **Reference method** | **Caries detection level** | | | **Dentin caries detection level** | | |
|  |  |  |  |  |  |  |  |  | **SE**  **(%)** | **SP**  **(%)** | **Az**  **(ROC)** | **SE**  **(%)** | **SP**  **(%)** | **Az**  **(ROC)** |
| Huth et al. (2008) | 120 | 6-51 | 120 | 120/- | 10 | Cav.prep. | Visually | 2 | - | - | - | - | - | - |
| Rechmann et al. (2012) | 100 | 13-58 | 433 | 343/90 | nr | - | Visually | nr | - | - | - | - | - | - |
| Sinanoglu et al. (2014) | 37 | 13-37 | 217 | 8 | 10 | Cav.prep. | Visually | 2 | - | - | 0.64-0.72 | - | - | - |
| nr-not reported; ^E^Ekstrand criteria | | | | | | | | | | | | | | |

Table S4a Results of systematic search of the literature on occlusal surfaces for in vitro validation studies on laser fluorescence

| **Laser Fluorescence examination** | **Study material** | | **Diagnostics** | **Validation methodology** | | | | **Validity** | | | | | |
| --- | --- | --- | --- | --- | --- | --- | --- | --- | --- | --- | --- | --- | --- |
| **In vitro validation studies on occlusal surfaces** | **Teeth**  **(N)** | **Molars**  **/PM**  **(N)** | **LF**  **criteria^1^** | **Hard tissue processing** | **Caries Staining** | **Visualisation /magnification** | **Scoring criteria^2^** | **Caries detection level** | | | **Dentin caries detection level** | | |
|  |  |  |  |  |  |  |  | **SE**  **(%)** | **SP**  **(%)** | **Az**  **(ROC)** | **SE**  **(%)** | **SP**  **(%)** | **Az**  **(ROC)** |
| ***DIAGNOdent 2095*** |  |  |  |  |  |  |  |  |  |  |  |  |  |
| Ferreira Zandona et al. (1998) | 150 | -/150 | 1 | Slices | Rhodamine B | Microsopy/Photo | 11 | 51 | 77 | - | - | - | - |
| Lussi et al. (1999) | 105 | -/- | 5 | Slices/Ground | 4 Dyes^S^ | Photography | 1 | 87/83 | 78/72 | - | 76/84 | 87/79 | - |
| Ando et al. (2000) | 100 | 100/- | 7 | Slices | Rhodamine B | Microscopy/Photo | 10 | - | - | - | 74 | 84.4 | - |
| Shi et al. (2000) | 76 | 48/28 | 8 | Slices | - | Micro Radiography | 1 | 42-46 | 95 | 0.92 | 78-82 | 100 | 0.99 |
| Pereira et al. (2001) | 101 | 101/- | 9 | Slices/Polish | Red 52 | MicroscopyPhoto | 10 | 97 | 13 | - | 20/17 | 98/98 | 0.69/0.65 |
| El-Housseiny et al. (2001) | 46 | - | 4 | Hemisection | - | Microscopy | 4 | 95 | 50 | - | - | - | - |
| Ouellet et al. (2002) | 100 | 100/- | 13 | Slices | Caries Det Dye | Photography | 10 | - | - | - | 73-94 | - | - |
| Alwas-Danowska et al. (2002) | 49 | 49/- | 10 | Hemisection | - | Microscopy/Photo | nr | - | - | - | 93-100 | 47-59 | 0.80-0.82 |
| Costa et al. (2002) | 50 | 25/25 | 11 | Slices | - | Microscopy | 4 | 78.5 | 94.1 | - | 78.5 | 88.9 | - |
| Tonioli et al. (2002) | 29 | 29/- | 5 | Slices | - | Microscopy | 4 | - | - | 0.79 | - | - | - |
| Bamzahim et al. (2002) | 87 | -/87 | 5 | Slices | - | Microscopy | 1 | - | - | - | 80 | 100 | - |
| Francescut et al. (2003) | 95 | 95/- | 27 | Slices/Ground | 4 Dyes^S^ | Microscopy | 1 | - | - | - | 73 | 65 | - |
| Kordic et al. (2003) | 61 | 36/25 | 5 | Hemise/Polish | - | Microscopy | 1 | 95.2 | 52.5 | 0.81 | 90.9 | 76 | 0.73 |
| Cortes et al. (2003) | 111 | 111/- | 14 | Slices | - | Microscopy | 14 | 73 | 85 | 0.84 | 84 | 67 | 0.81 |
| Lussi et al. (2003) | 95 | -/- | 11 | Grounding | 4 Dyes^S^ | Photography | 10 | - | - | - | 85 | 82 | - |
| Baseren et al. (2003) | 35 | 35/- | 27 | Hemisection | - | Microscopy | 5 | - | - | 74 | 66 | - | - |
| Fung et al. (2004) | 25 | 25/- | 27 | Slices | - | Microscopy | 3 | - | - | - | 44-67 | 81-94 | - |
| Lizarelli et al. (2004) | 22 | 11/11 | 6 | Hemisection | Fuchsin | Microscopy | nr | - | - | - | - | - | - |
| Reis et al. (2004) | 45 | -/- | 17 | Hemisection | - | Microscopy | 10 | - | - | - | 35-46 | 90-94 | - |
| Angnes et al. (2005) | 57 | 57/- | 16 | Hemisection | - | Microscopy | 10 | - | - | - | 81.2/75 | 60.6/66 | 0.67/0.76 |
| Burin et al. (2005) | 54 | 54/- | 15 | Hemisection | - | Microscopy | 10 | - | - | - | 72-77 | 67-71 | 0.67-0.71 |
| Deery et al. (2006) | 37 | 25/12 | 1 | Slices | - | Microscopy | 2 | 93 | 7 | - | 63-90 | 33-88 | - |
| Deery et al. (2006) | 37 | 25/12 | 5 | Slices | - | Microscopy | 2 | 91 | 47 | - | 63-90 | 33-88 | - |
| Lussi and Hellwig (2006) | 119 | 119/- | 19 | Grounding | Rhodamine B | Microscopy/Photo | 1 | 96 | 69 | - | 81 | 79 | - |
| Reis et al. (2006) | 57 | 57/- | 17 | Slices/Polish | - | Microscopy | 10 | 71 | 57 | - | 78 | 63 | - |
| nr-not reported;  ^1^See Table LF Diagnostic criteria; ^2^See Table semiquantitative histological criteria;  ^S^Staining (methyl methacrilate-MMA, Rhodamine B, Basic Fuchsin, Acetyl Light Green-ALG: 4 Dyes) | | | | | | | | | | | | | |

| **Laser Fluorescence examination** | **Study material** | | **Diagnostics** | **Validation methodology** | | | | **Validity** | | | | | |
| --- | --- | --- | --- | --- | --- | --- | --- | --- | --- | --- | --- | --- | --- |
| **In vitro validation studies on occlusal surfaces** | **Teeth**  **(N)** | **Molars/PM**  **(N)** | **LF**  **criteria^1^** | **Hard tissue processing** | **Caries Staining** | **Visualisation /magnification** | **Scoring criteria^2^** | **Caries detection level** | | | **Dentin caries detection level** | | |
|  |  |  |  |  |  |  |  | **SE**  **(%)** | **SP**  **(%)** | **Az**  **(ROC)** | **SE**  **(%)** | **SP**  **(%)** | **Az**  **(ROC)** |
| Souza-Zaroni et al. (2006) | 47 | 47/- | 18 | Slices/Ground | - | Microscopy | 1 | 35-45 | 82-99 | - | - | - | - |
| Manton et al. (2007) | 67 | 67/- | 27 | Slices | - | Microscopy | 14 | - | - | - | 49 | 83 | - |
| Rodrigues et al. (2008) | 119 | 119/- | 19 | Grounding | Rhodamine B | Microscopy/Photo | 1 | - | - | - | 51 | 89 | 0.81 |
| Valera FB et al. (2008) | 72 | 54/18 | 2 | Slices/Ground | - | Microscopy/Photo | 5 | 33 | 100 | 0.67 | - | - | - |
| Pereiera et al. (2009) ^E^ | 96 | 96/- | 1 | Slices | - | Microscopy | 10 | - | - | - | - | - | - |
| Sridhar et al. (2009) ^E^ | 50 | 17/33 | 20 | Hemisection | MMA | Microscopy | 10 | - | - | - | - | - | - |
| Rodrigues et al. (2009) | 148 | - | 19 | Hemisection | - | Microscopy | 1 | 53 | 92 | 0.83 | 16 | 89 | 0.68 |
| Rodrigues and Hug (2009) | 119 | 119/- | 19 | Grounding | Rhodamine B | Microscopy/Photo | 1 | - | - | - | 57 | 80 | 0.78 |
| De Paula et al. (2011) | 26 | 26/- | 1 | Hemisection | - | Microcopy | 6 | 72 | 100 | 0.86 | - | - | - |
| Jablonski-Momeni et al. (2011) | 100 | 85/15 | 19 | Slices | - | Microscopy | 2 | 82 | 48 | 0.75 | 54 | 89 | 0.79 |
| Rodrigues et al. (2011) | 97 | 97/- | 19 | Grounding | Rhodamine B | Microscopy | 5 | 70 | 76.5 | 0.72 | 63 | 88.6 | 0.85 |
| Jablonski Momeni et al. (2012) | 36 | 36/- | 19 | Slices | - | Photography | 2 | 83/85 | 65/70 | 0.82/0.81 | 82/77 | 59/52 | 0.71/0.72 |
| Oancea et al. (2013) | 240 | 122/118 | 19 | Hemisection | - | Microscopy | 2 | - | - | 0.71 | - | - | - |
|  |  |  |  |  |  |  |  |  |  |  |  |  |  |
| ***DIAGNOdent Pen 2190*** |  |  |  |  |  |  |  |  |  |  |  |  |  |
| Lussi and Hellwig (2006) | 119 | 119/- | 19 | Grounding | Rhodamine B | Microscopy/Photo | 1 | 88/91 | 77/77 | - | 79/86 | 84/71 | - |
| Rodrigues et al. (2008) | 119 | 119/- | 19 | Grounding | Rhodamine B | Microscopy/Photo | 1 | - | - | - | 56 | 78 | 0.79 |
| Rodrigues and Hug (2009) | 119 | 119/- | 19 | Grounding | Rhodamine B | Microscopy/Photo | 1 | - | - | - | 87 | 56 | 0.76 |
| Rodrigues et al. (2011) | 97 | 97/- | 19 | Grounding | Rhodamine B | Microscopy | 5 | 62.5 | 76.5 | 0.72 | 63 | 87.1 | 0.85 |
| Aktan et al. (2012) | 82 | 82/- | 19 | Hemisection | - | Microscopy | 4 | 65/65 | 33/43 | 0.33/0.76 | 33/43 | 49/60 | 0.69/0.75 |
| Seremidi et al. (2012) | 41 | -/41 | 21 | Grounding | - | Microscopy/Photo | 2 | 54 | 65.9 | 0.60 | 55 | 86.2 | 0.71 |
| Achilleos et al. (2013) ^E^ | 38 | 12/26 | 22 | Slices | - | Photography | 10 | 75/66 | 50/50 | 0.58/0.38 | - | - | - |
| Arslan et al. (2014) | 60 | - | 19 | Slices | - | Microscopy/M-CT | 2 | - | - | - | - | - | - |
| Mortensen et al. (2014) | 100 | -/- | 1 | Hemisection | - | Microscopy | 2 | 8-47 | 54-96 | 0.52-0.56 | 7-49 | 54-93 | 0.50-0.54 |
| Bussaneli et al. (2015) | 94 | - | 29 | Slices | - | Microscopy | 19 | - | - | - | 66.2 | 83.2 | 0.76 |
| Ozturk et al. (2015) | 44 | 44/- | 23 | Slices | - | Microscopy | 17 | - | - | - | 86 | 68-71 | 0.77-0.78 |
| Iranzo-Cortez et al. (2017) | 64 | 37/28 | ? | Slices |  | Microscopy | 18 | 85 | 53 | 0.73 |  |  |  |
|  |  |  |  |  |  |  |  |  |  |  |  |  |  |
| nr-not reported;  ^1^See Table LF Diagnostic criteria; ^2^See Table semiquantitative histological criteria;  ^S^Staining (methyl methacrilate-MMA, Rhodamine B, Basic Fuchsin, Acetyl Light Green-ALG: 4 Dyes) | | | | | | | | | | | | | |

###

## Table S4b Results of systematic search of the literature on occlusal surfaces for in vivo validation studies on laser fluorescence

| **Laser Fluorescence examination** | **Study material** | | | | **Diagnostics** | **Validation methodology** | | | **Validity** | | | | | | | | | |
| --- | --- | --- | --- | --- | --- | --- | --- | --- | --- | --- | --- | --- | --- | --- | --- | --- | --- | --- |
| **In vivo validation studies on occlusal surfaces** | **Patients**  **(N)** | **Age**  **(years)** | **Teeth (N)** | **Molars/PM (N)** | **Criteria** | **Histology technique** | **Validation** | **Reference method** | **Caries detection level** | | | | | **Dentin caries detection level** | | | | |
|  |  |  |  |  |  |  |  |  | **SE**  **(%)** | **SP**  **(%)** | **Az**  **(ROC)** | | | **SE**  **(%)** | | | **SP**  **(%)** | **Az**  **(ROC)** |
|  |  |  |  |  |  |  |  |  |  |  |  | | |  | | |  |  |
| ***DIAGNOdent 2095*** |  |  |  |  |  |  |  |  |  |  |  | | |  | | |  |  |
| Lussi et al. (2001) | 240 | 8-20 | 332 | 246/86 | 27 | Cav.prep. | Visually | 2 | 96 | - | - | | | 92 | | | 86 | - |
| Sheehy et al. (2001) ^E^ | 170 | 4-8 | 170 | 170/- | 5 | - | Visually | 14 | - | - | - | | | - | | | - | - |
| Heinrich-Weltzien et al. (2002) | 97 | 17-21 | 248 | 248/- | 24 | Cav.Prep | Visually | 2 | - | - | - | | | 93 | | | 63 | - |
| Alwas Danowska et al. (2002) | 13 | 18-25 | 45 | 45/- | 1 | - | Visually | nr | - | - | - | | | - | | | - | - |
| Anttonen et al. (2003) ^E^ | 109 | 7-14 | 613 | 613/- | 3 | Drilling | Visually | 10 | - | - | - | | | 92 | | | 82 | - |
| Astvaldsdotttir et al. (2004) | nr | 18-30 | 34 | nr | 1 | Cav Prep. | Visual/Photo | 2 | - | - | - | | | - | | | - | - |
| Traneus et al. (2004) | 30 | 18-42 | 52 | 52/- | 3 | Cav.prep. | Visual/Photo | 6 | - | - | - | | | - | | | - | - |
| Angnes et al. (2005) ^E^ | 38 | 19-35 | 57 | 57/- | 16 | Hist.prep. | In vitro | 10 | - | - | - | | 69/81 | | 56.4/54 | | | 0.64/0.69 |
| Akarsu et al. (2006) | 161 | 18-25 | 165 | 165/- | 3 | Cav.prep. | Visually | 6 | 88/88 | 71/71 | - | | 89/89 | | 87/87 | | | - |
| Olmez et al. (2006) ^E^ | 28 | 7-17 | 92 | 77/15 | 24 | Cav.prep. | Visually | 2 | - | - | - | | 86 | | 80 | | | - |
| Reis et al. (2006) ^E^ | 38 | 19-30 | 57 | 57/- | 17 | Hist.prep. | In vitro | 10 | 71 | 57 | - | | 78 | | 63 | | | - |
| Krause et al. (2007) | 82 | 28-44 | 94 | 39/55 | 3 | Cav.prep. | Visually^P^ | 1 | - | - | - | | 92.6 | | 53.7 | | | 0.69 |
| Barberia et al. (2008) ^E^ | nr | 6-14 | 77 | 77/- | 3 | - | Visually | 14 | 40 | 82 | - | | - | | - | | | - |
| Costa et al. (2008) | 26 | 10-13 | 151 | nr | 30 | Cav.Prep | Visually* | 6 | - | - | - | | 93 | | 75 | | | - |
| Abalos et al. (2008) | 46 | 19-29 | 102 | 102/- | nr | Cav.Prep | Visually^P^ | 7 | - | - | - | | 89 | | 75 | | | 0.85 |
| Diniz and Rodrigues et al. (2009) | 35 | 7-12 | 130 | 130/- | 29 | Drilling | Visually | 2 | - | - | - | | 70-81 | | 57-87 | | | 0.81 |
| Rando-Meirelles et al. (2011) ^E^ | 327 | 12-15 | 789 | 789/- | 30 | - | Radiography | 8 | - | - | - | | 64 | | 74 | | | - |
| Abalos et al. (2012) ^E^ | 44 | 19-29 | 96 | 96/- | 26 | Cav.prep. | Visually^P^ | 14 | 97 | 63 | 0.83 | | - | | - | | | - |
| Diniz et al. (2012) | 88 | 18-35 | 105 | 65/40 | 3 | Hist.prep. | In vitro | 1 | 85 | 100 | 0.94 | | 81 | | 77 | | | 0.84 |
| Rechmann et al. (2012) | 100 | 13-58 | 433 | 343/90 | 31 | - | Visually | nr | - | - | - | | - | | - | | | - |
| Bozdemir et al. (2013) | 37 | nr | 156 | 142/14 | 3 | Cav.prep. | Visual/Photo | 2 | 95-97 | 58-60 | | 0.90-0.92 | | 74-81 | | 76-81 | | 0.82-0.85 |
| Castilho et al. (2016) ^E^ | 26 | 16-39 | 43 | 43/- | 3 | Hist.prep. | In vitro | 10 | 25 | 85 | | 0.55 | | 85 | | 100 | | 0.92 |
| Melo et al. (2017) | 152 | 14-39 | 302 | 148/154 | 3 | Cav.prep. | Visually | 24 | - | - | | - | | 92.4 | | 92.7 | | 0.95 |

| **Laser Fluorescence examination** | **Study material** | | | | **Diagnostics** | **Validation methodology** | | | **Validity** | | | | | | | | | |
| --- | --- | --- | --- | --- | --- | --- | --- | --- | --- | --- | --- | --- | --- | --- | --- | --- | --- | --- |
| **In vivo validation studies on occlusal surfaces** | **Patients**  **(N)** | **Age**  **(years)** | **Teeth (N)** | **Molars/PM (N)** | **Criteria** | **Histology technique** | **Validation** | **Reference method** | **Caries detection level** | | | | | **Dentin caries detection level** | | | | |
|  |  |  |  |  |  |  |  |  | **SE**  **(%)** | **SP**  **(%)** | **Az**  **(ROC)** | | | **SE**  **(%)** | | | **SP**  **(%)** | **Az**  **(ROC)** |
|  |  |  |  |  |  |  |  |  |  |  |  | |  | |  | | |  |
| ***DIAGNOdent Pen 2190*** |  |  |  |  |  |  |  |  |  |  |  | |  | |  | | |  |
| Krause et al. (2007) | 82 | 28-44 | 94 | 39/55 | 3 | Cav.prep. | Visually^P^ | 1 | - | - | - | | 88.9 | | 53.7 | | | 0.67 |
| Huth et al. (2008) | 120 | 6-51 | 120 | 120/- | nr | Cav.prep. | Visual/Photo | 2 | 88 | 85 | 0.92 | | 67 | | 79 | | | 0.78 |
| Diniz et al. (2012) | 88 | 18-35 | 105 | 65/40 | 3 | Hist.prep. | In vitro | 1 | 89 | 80 | 0.95 | | 85 | | 71 | | | 0.79 |
| Sinanoglu et al. (2014) | 37 | 13-nr | 217 | nr | 3 | Cav.prep. | Visually | 2 | 98/98 | 7-15 | 0.55-0.64 | | 57-68 | | 38-67 | | | - |
| Bahrololoomi et al. (2015) | 31 | 7-13 | 109 | 109/- | nr | Cav.prep. | Visually | 6 | 71/86 | 71/71 | | - | | 90/87 | | 89/85 | | - |
|  |  |  |  |  |  |  |  |  |  |  | |  | |  | |  | |  |
| nr-not reported; *Enameloplasty-fissure eradiation; ^E^Ekstrand criteria; P-using probe | | | | | | | | | | | | | | | | | | |

## Table S5a Results of systematic search of the literature on occlusal surfaces for in vitro validation studies on FOTI

| **FOTI examination** | **Study material** | | **Diagnostics** | **Validation methodology** | | | | **Validity** | | | | | |
| --- | --- | --- | --- | --- | --- | --- | --- | --- | --- | --- | --- | --- | --- |
| **In vitro validation studies on occlusal surfaces** | **Teeth**  **(N)** | **Molars/PM**  **(N)** | **FOTI**  **Criteria^1^** | **Hard tissue processing** | **Caries Staining** | **Visualisation /magnification** | **Scoring criteria^2^** | **Caries detection level** | | | **Dentin caries detection level** | | |
|  |  |  |  |  |  |  |  | **SE**  **(%)** | **SP**  **(%)** | **Az**  **(ROC)** | **SE**  **(%)** | **SP**  **(%)** | **Az**  **(ROC)** |
| Wenzel et al. (1992) | 81 | 81/- | 2 | Slices | - | Microscopy | 7 | - | - | 0.80 | - | - | 0.87 |
| Schneiderman et al. (1997) | 25 | 14/12 | nr | Slices | SVG | Microscopy | nr | 67/80 | 87/87 | - | - | - | - |
| Ashley et al. (1998) | 103 | 35/68 | 3 | Slices | - | Microscopy | 2 | 21 | 88 | - | 14 | 95 | - |
| Cortes et al. (2000) | 59 | 59/- | 4 | Slices | - | Microscopy/Photo | 14 | - | - | - | 31 | 100 | 0.85 |
| Grossman et al. (2002) ^E^ | 214 | - | 6 | Slices | - | Microscopy | 10 | - | - | - | 54^E^ | 90^E^ | - |
| Cortes et al. (2003) | 111 | 111/- | 5 | Slices | - | Microscopy | 14 | 98 | 50 | 0.88 | 66 | 96 | 0.89 |
| Manton et al. (2007) | 67 | 67/- | 4 | Slices | - | Microscopy | 14 | - | - | - | 36 | 93 | - |
| Gomez et al. (2013) ^E^ | 112 | - | 4 | Hemisection | - | Photography | 10 | 80 | 84 | 0.97 | 78 | 92 | 0.96 |
|  |  |  |  |  |  |  |  |  |  |  |  |  |  |
| nr-not reported;  ^1^ See Table FOTI Diagnostic criteria; ^2^See Table semiquantitative histological criteria;  ^S^Staining- SVG-Stevenels blue and Van Gieson picro-fuchsin | | | | | | | | | | | | | |

## Table S5b Results of systematic search of the literature on occlusal surfaces for in vivo validation studies on FOTI

| **FOTI examination** | **Study material** | | | | **Diagnostics** | **Validation methodology** | | | | **Validity** | | | | | |
| --- | --- | --- | --- | --- | --- | --- | --- | --- | --- | --- | --- | --- | --- | --- | --- |
| **In vivo validation studies on occlusal surfaces** | **Patients**  **(N)** | **Age**  **(years)** | **Teeth (N)** | **Molars/PM (N)** | **Criteria** | **Histology technique** | **Validation** | **Reference method** | **Caries detection level** | | | | **Dentin caries detection level** | | |
|  |  |  |  |  |  |  |  |  | **SE**  **(%)** | | **SP**  **(%)** | **Az**  **(ROC)** | **SE**  **(%)** | **SP**  **(%)** | **Az**  **(ROC)** |
| Verdonshoot et al. (1992) | 13 | 7-13 | 23 | 19/4 | 1 | Cav.prep. | Visually | 7 | - | | - | - | 13 | 99 | - |

## Table S6a Results of systematic search of the literature on occlusal surfaces for in vitro validation studies on QLF

| **QLF examination** | **Study material** | | **Diagnostics** | **Validation methodology** | | | | **Validity** | | | | | |
| --- | --- | --- | --- | --- | --- | --- | --- | --- | --- | --- | --- | --- | --- |
| **In vitro validation studies on occlusal surfaces** | **Teeth**  **(N)** | **Molars/PM**  **(N)** | **QLF**  **Criteria^1^** | **Hard tissue processing** | **Caries Staining** | **Visualisation /magnification** | **Scoring criteria^2^** | **Caries detection level** | | | **Dentin caries detection level** | | |
|  |  |  |  |  |  |  |  | **SE**  **(%)** | **SP**  **(%)** | **Az**  **(ROC)** | **SE**  **(%)** | **SP**  **(%)** | **Az**  **(ROC)** |
| Ando et al. (2000) ^E^ | 100 | 100/- | 1 | Slices | Rhodamine B | Microscopy/Photo | 10 | - | - | - | 92 | 56 | - |
| Kuehnisch et al. (2006) | 54 | 54/- | ? | Slices/Ground | - | Microscopy | 2 | - | - | - | 80/84 | 86.2/89.7 | 0.88-0.89 |
| Kuehnisch et al. (2006) | 54 | 54/- | ? | Slices/Ground | - | Microradiography | 2 | - | - | - | 90.9/86.4 | 84.4/90.6 | 0.91-0.93 |
| Pereiera et al. (2009) ^E^ | 96 | 96/- | 1 | Slices | - | Microscopy | 10 | - | - | - | - | - | - |
| Gomez et al. (2013) ^E^ | 112 | - | 4 | Hemisection | - | Photography | 10 | 62/72 | 94/91 | 0.94/0.97 | 93/86 | 80/81 | 0.89/0.92 |
| Bussaneli et al. (2015) ^E^ | 94 | - | 3 | Slices | - | Microscopy | 10 | - | - | - | 69.6 | 83.2 | 0.76 |
| Jallad et al. (2015) ^E^ | 60 | 30/30 | nr | Slices/Ground | Rhodamine B | Microscopy/Photo | 10 | - | - | - | 87/89 | 60/82 | 0.90 |
| nr-not reported;  ^1^ See Table QLF Diagnostic criteria; ^2^ See Table semiquantitative histological criteria; | | | | | | | | | | | | | |

## Table S6b Results of systematic search of the literature on occlusal surfaces for in vivo validation studies on QLF

| **QLF examination** | **Study material** | | | | **Diagnostics** | **Validation methodology** | | | | **Validity** | | | | | |
| --- | --- | --- | --- | --- | --- | --- | --- | --- | --- | --- | --- | --- | --- | --- | --- |
| **In vivo validation studies on occlusal surfaces** | **Patients**  **(N)** | **Age**  **(years)** | **Teeth (N)** | **Molars/PM (N)** | **Criteria** | **Histology technique** | **Validation** | **Reference method** | **Caries detection level** | | | | **Dentin caries detection level** | | |
|  |  |  |  |  |  |  |  |  | **SE**  **(%)** | | **SP**  **(%)** | **Az**  **(ROC)** | **SE**  **(%)** | **SP**  **(%)** | **Az**  **(ROC)** |
| Alammari et al. (2013) | 46 | 18-75 | 46 | nr | 2 | Cav.Prep | Visual/Photo | nr | - | | - | - | - | - | - |
| nr-not reported | | | | | | | | | | | | | | | |

# Results from the quality assessment of the selected diagnostic studies

## Table S7 The set of criteria used for risk of bias (RoB) rating applied during the review

| **Signalling questions** | **RoB** | **Description of the criteria (Domain 1)** | | | | |
| --- | --- | --- | --- | --- | --- | --- |
| ***Patient selection bias***  1. Is an eligible sample selected from the study participants/population?  *In vivo studies only* | Indicators for low RoB | The eligible sample from the study participants/population is representative of the patients for whom the results of the study was applicable. The eligible sample is homogenous, enrolled consecutively or randomly, and obtained from the research question, e.g., PIRD. | | | | |
|  | Indicators for high RoB | The eligible sample is not representative, the clinical indication for the application of the diagnostic test(s) is not completely described, and the sample is not enrolled consecutively or randomly; there is no research question. | | | | |
|  | Response options | Yes  (low RoB) | Most likely yes  (low RoB) | Most likely no  (high RoB) | No  (high RoB) | No information  (unclear) |
| ***Tooth selection bias***  2. Is an eligible sample of teeth selected? | Indicators for low RoB | Eligible selection of the target teeth and surfaces:   - In vitro/in vivo studies on *occlusal* caries detection = permanent molars/primary molars - In vitro/in vivo studies on *proximal* caries detection = permanent molars & premolars/primary molars. | | | | |
|  | Indicators for high RoB | The selected teeth and surfaces are not homogeneous; there is over/underrepresentation of at least one group of teeth, a mixture of posterior and anterior teeth, a mixture of permanent and primary teeth or a mixture of occlusal surfaces from premolars and molars. | | | | |
|  | Response options | Yes  (low RoB) | Most likely yes  (low RoB) | Most likely no  (high RoB) | No  (high RoB) | No information  (unclear) |

| **Signalling questions** | **RoB** | **Description of the criteria (Domain 1)** | | | | |
| --- | --- | --- | --- | --- | --- | --- |
| ***Spectrum bias***  3. Is an appropriate spectrum of caries lesions selected? | Indicators for low RoB | All stages of caries (e.g., sound/enamel/dentin caries/caries at least in the inner half of the dentin or non-cavitated/cavitated caries) are included. The sampled caries spectrum should be pre-assessed. | | | | |
|  | Indicators for high RoB | At least one stage of caries is excluded or under/over-represented in the study. | | | | |
|  | Response options | Yes  (low RoB) | Probably yes  (low RoB) | Probably no  (high RoB) | No  (high RoB) | No information  (unclear) |
| ***Sample size***  4. Is the sample size appropriate for validity and reproducibility testing? | Indicators for low RoB | The sample size is statistically determined. | | | | |
|  | Indicators for high RoB | There is no sample size calculation, etc. | | | | |
|  | Response options | Yes  (low RoB) | Probably yes  (low RoB) | Probably no  (high RoB) | No  (high RoB) | No information  (unclear) |

| **Signalling questions** | **RoB** | **Description of the criteria (Domain 2)** | | | | |
| --- | --- | --- | --- | --- | --- | --- |
| ***Index test criteria***  5. Do/does the index test(s) correctly classify the target condition? | Indicators for low RoB | Exact pre-definition/prescription of the criteria used, thresholds for the index test. Correct usage of the index test(s) according to latest recommendations (justified on the basis of the references). | | | | |
|  | Indicators for high RoB | Modifications of the index test(s), mis-usage, misinterpretation. | | | | |
|  | Response options | Yes  (low RoB) | Probably yes  (low RoB) | Probably no  (high RoB) | No  (high RoB) | No information  (unclear) |
| ***Blinding bias***  ***(index test)***  6. Are the index test(s) data interpreted without knowledge of the results of the reference standard? | Indicators for low RoB | Appropriate blinding of the examiners who are making the decisions/diagnoses from index test(s), e.g., at least a one-week interval between examinations, randomized/shuffled allocation of the order of specimen/images and/or inclusion of multiple examiners who are performing only one test each. | | | | |
|  | Indicators for high RoB | Insufficient blinding. Same examiner performed multiple tests. | | | | |
|  | Response options | Yes  (low RoB) | Probably yes  (low RoB) | Probably no  (high RoB) | No  (high RoB) | No information  (unclear) |
| ***Calibration bias***  ***(index test)***  7. Were the examiners trained/calibrated for the performing the index test(s)? | Indicators for low RoB | Details and outcomes of the calibration training, including the Kappa values for intra- and inter-examiner reliability, are given. Calibration training must include an independent sample of individuals or teeth. Calibration data should not be interpreted/misunderstood as intra-examiner reliability. | | | | |
|  | Indicators for high RoB | Insufficient training/calibration. | | | | |
|  | Response options | Yes  (low RoB) | Probably yes  (low RoB) | Probably no  (high RoB) | No  (high RoB) | No information  (unclear) |

| **Signalling questions** | **RoB** | **Description of the criteria (Domain 3)** | | | | |
| --- | --- | --- | --- | --- | --- | --- |
| ***Reference test criteria***  8. Does the reference test correctly classify the target condition? | Indicators for low RoB | Usage of an optimal (“perfect”) reference standard, e.g., histology, microradiography or µCT. Exact pre-definition/prescription of the used criteria, thresholds for the reference test. Correct usage of the reference test according to the latest recommendations (justified on the basis of references). The reference test is conditionally independent of the index tests. | | | | |
|  | Indicators for high RoB | Usage of a sub-optimal (“imperfect”) reference standard, e.g., radiography. Modifications of the reference test; mis-usage; misinterpretation. The reference test is conditionally not independent of the index test. Differential misclassification – the error rate is associated with the index test results. Non-differential misclassification – the error rate is independent of the index test results, but this can underestimate SE and SP. | | | | |
|  | Response options | Yes  (low RoB) | Probably yes  (low RoB) | Probably no  (high RoB) | No  (high RoB) | No information  (unclear) |
| ***Blinding bias***  ***(reference test)***  9. Is the reference test data interpreted without knowledge of the results of the index test(s)? | Indicators for low RoB | Appropriate blinding of the examiners who are making the decisions/diagnoses from  reference test(s). For example, there is at least a one-week interval between examinations, randomized/shuffled allocation of the order of specimen/images and/or inclusion of multiple examiners, who are performing only one test each, with unawareness of the outcome of index test(s). | | | | |
|  | Indicators for high RoB | Insufficient blinding. For example, the same examiner performs multiple tests within a few days. | | | | |
|  | Response options | Yes  (low RoB) | Probably yes  (low RoB) | Probably no  (high RoB) | No  (high RoB) | No information  (unclear) |
| ***Calibration bias***  ***(reference test)***  10. Are the examiners trained/calibrated for performing the reference test(s)? | Indicators for low RoB | Details and outcomes of the calibration training, including Kappa values for intra- and inter-examiner reliability, are given. Calibration training must include an independent sample of individuals or teeth. Calibration data should not be interpreted/misunderstood as intra-examiner reliability. | | | | |
|  | Indicators for high RoB | Insufficient training/calibration. | | | | |
|  | Response options | Yes  (low RoB) | Probably yes  (low RoB) | Probably no  (high RoB) | No  (high RoB) | No information  (unclear) |
| **Signalling questions** | **RoB** | **Description of the criteria (Domain 4)** | | | | |
| ***Incorporation bias***  11. Are the reference test(s) performed separately from the index test(s)? | Indicators for low RoB | The reference and index test are performed separately. | | | | |
|  | Indicators for high RoB | The index test is incorporated in a (composite) reference test; the result of the index test is explicitly used as a criterion for the reference test. | | | | |
|  | Response options | Yes  (low RoB) | Probably yes  (low RoB) | Probably no  (high RoB) | No  (high RoB) | No information  (unclear) |
| ***Partial verification bias***  12. Do all patients/teeth/surfaces undergo both the reference tests and the index tests? | Indicators for low RoB | Ensure that all patients/teeth/surfaces undergo both the reference tests and the index tests. | | | | |
|  | Indicators for high RoB | Identified when a non-random set of patients/teeth/surfaces does not undergo the reference test and the verification rate depends on the index test results. | | | | |
|  | Categories  of RoB | Yes  (low RoB) | Probably yes  (low RoB) | Probably no  (high RoB) | No  (high RoB) | No information  (unclear) |
| ***Differential verification bias***  13. Do all patients/teeth/surfaces receive the same reference standard? | Indicators for low RoB | Ensure that all patients/teeth/surfaces receive the same reference standard. | | | | |
|  | Indicators for high RoB | Identified when a non-random set of patients/teeth/surfaces is verified with a second or third reference test, especially when this selection depends on the index test result. | | | | |
|  | Response options | Yes  (low RoB) | Probably yes  (low RoB) | Probably no  (high RoB) | No  (high RoB) | No information  (unclear) |

| **Signalling questions** | **RoB** | **Description of the criteria (Domain 4)** | | | | |
| --- | --- | --- | --- | --- | --- | --- |
| ***Bias in the analysis***  14. Are all patient/teeth/surfaces, uninterpretable or intermediate test results and withdrawals included in the analysis? | Indicators for low RoB | All patients (teeth) who entered the study are accounted for, and all uninterpretable or intermediate test results and withdrawals (including lost specimens of the teeth) are explained. | | | | |
|  | Indicators for high RoB | Not all patients (teeth) who entered the study are accounted for, and not all uninterpretable or intermediate test results and withdrawals (including lost specimens of the teeth) are explained. | | | | |
|  | Response options | Yes  (low RoB) | Probably yes  (low RoB) | Probably no  (high RoB) | No  (high RoB) | No information  (unclear) |
| ***Validity bias***  15. Are the validation of results for the test method(s) included in the analysis? | Indicators for low RoB | Full presentation of results: Cross-tabulation (or distribution) of the index and reference test results by the reference standard results. Estimates of diagnostic accuracy and their precision are included (SE, SP, Az value). | | | | |
|  | Indicators for high RoB | Insufficient/incomplete information’s, e.g., missing 2x2 contingency tables and/or SE, SP, Az values. Incorrect statistics. Biased interpretation. | | | | |
|  | Response options* | Yes  (low RoB) | Probably yes  (low RoB) | Probably no  (high RoB) | No  (high RoB) | No information  (unclear) |
| ***Reproducibility bias***  16. Are the reliability data of results for the test method(s) included in analysis? | Indicators for low RoB | Full presentation of results: Intra- and inter-examiner reliability for all examiners and for all teeth. Correct statistical procedures, e.g., Kappa values, Bland-Altman-Plots, etc. Data from the calibration training have not been mis/interpreted as reliability. | | | | |
|  | Indicators for high RoB | Insufficient/incomplete information on intra- and inter-examiner reliability or incorrect statistics. Data for calibration purposes only. Biased interpretation. | | | | |
|  | Response options | Yes  (low RoB) | Probably yes  (low RoB) | Probably no  (high RoB) | No  (high RoB) | No information  (unclear) |

## Table S8a Risk of bias assessment for visual examination of in vitro validation studies on occlusal surfaces

| **Visual examination of in vitro validation studies on occlusal surfaces** | **Signaling questions** | | | | | | | | | | | | | | | |
| --- | --- | --- | --- | --- | --- | --- | --- | --- | --- | --- | --- | --- | --- | --- | --- | --- |
|  | **Selection bias** | | | | **Index test bias** | | | **Reference test bias** | | | | **Verification bias** | | | **Outcome bias** | |
|  | Patient  selection | Teeth selection | Caries Spectrum | Sample size | Test Criteria | Blinding bias | Calibration bias | Test Criteria | Blinding bias | Calibration bias | Incorporation bias | Partial ver. bias | Differential ver. bias | Bias in the Analysis | Validity bias | Reproducibility bias |
| Kay et al. (1988) | **x** | - | - | - | - | - | - | ? | + | - | + | + | + | + | ? | - |
| Wenzel et al. (1990) | **x** | - | + | - | + | + | - | + | ? | - | + | + | + | + | - | - |
| Lussi et al. (1991) | **x** | - | - | - | + | + | - | + | ? | - | + | + | + | + | - | - |
| Nytun et al. (1992) | **x** | + | - | - | + | + | - | + | ? | - | + | + | + | + | - | - |
| Wenzel and Fejerskov (1992) | **x** | + | ? | - | + | + | ? | + | - | - | - | + | + | + | + | - |
| Wenzel et al. (1992) | **x** | + | - | - | ? | ? | - | + | ? | - | + | + | + | + | - | - |
| Ketley and Holt (1993) | **x** | + | - | - | + | + | + | + | + | - | + | + | + | + | - | + |
| Lussi et al. (1993) | **x** | + | + | - | + | + | - | + | + | - | + | + | + | + | - | + |
| Verdonshoot et al. (1993) | **x** | + | - | - | ? | + | - | + | ? | - | + | + | + | + | - | - |
| Tveit et al. (1994) | **x** | - | - | - | + | + | - | + | ? | - | + | + | + | + | + | - |
| Wenzel et al. (1994) | **x** | + | - | - | ? | ? | - | + | + | - | + | + | + | + | ? | + |
| Deery et al. (1995) | **x** | + | - | - | + | + | - | + | + | - | + | + | + | + | ? | + |
| Ricketts et al. (1995) ^a^ | **x** | + | - | - | + | ? | - | + | ? | - | + | + | + | + | - | - |
| Ricketts et al. (1995) ^b^ | **x** | - | - | - | + | ? | - | + | ? | - | + | + | + | + | - | - |
| Ekstrand et al. (1997) | **x** | - | - | - | + | + | + | + | + | - | + | + | + | + | + | + |
| Gray and Paterson (1997) | **x** | + | + | - | + | + | - | + | + | - | + | + | + | + | - | - |
| Ashley et al. (1998) | **x** | - | - | - | + | ? | - | + | ? | - | + | + | + | + | - | ? |
| Ferreira Zandona et al. 1998 | **x** | - | - | - | + | + | + | + | + | - | + | + | + | + | - | ? |
| Huysmans et al. (1998) | **x** | - | - | - | + | - | - | + | + | - | + | + | + | + | - | - |
| Ando et al. (2000) | **x** | **-** | **-** | **-** | + | ? | + | **?** | **?** | **-** | + | + | + | + | ? | ? |
| Cortes et al. (2000) | **x** | + | - | **-** | + | - | + | + | ? | **-** | + | + | + | + | + | ? |
| El-Housseiny et al. (2001) | **x** | **-** | **-** | **-** | + | **+** | **-** | + | **+** | **-** | + | + | + | + | **-** | **-** |
| Pereira et al. (2001) | **x** | + | **-** | **-** | + | ? | + | + | + | **-** | + | + | + | + | **?** | - |
| Alwas-Danowska et al. (2002) | **x** | + | - | **-** | + | + | **-** | **-** | + | **-** | + | + | + | + | **-** | - |
| Costa et al. (2002) | **x** | **-** | ? | **-** | + | + | **+** | + | ? | **-** | + | + | + | + | **-** | + |
| Grosman et al. (2002) | **x** | **-** | **-** | **-** | + | ? | + | + | + | - | + | + | + | + | **-** | **-** |
| Tonioli et al. (2002) | **x** | + | **-** | **-** | + | + | ? | + | + | **-** | + | + | + | + | **-** | **-** |
| Cortes et al. (2003) | **x** | + | **-** | **-** | + | ? | - | + | ? | **-** | + | + | + | + | + | - |
| Forgie et al. (2003) | **x** | **-** | **-** | **-** | + | + | **-** | + | + | **?** | + | + | + | + | **-** | **-** |
| Kordic et al. (2003) | **x** | **-** | **-** | **-** | + | + | **-** | + | + | **-** | + | + | + | + | **-** | **?** |
| Lussi et al. (2003) | **x** | **?** | **-** | **-** | + | + | **-** | + | + | **-** | + | + | + | + | **-** | **?** |
| Fung et al. (2004) | **x** | + | - | **-** | + | ? | **-** | - | ? | **-** | + | + | + | + | ? | **+** |
| Lizarelli et al. (2004) | **x** | - | - | **-** | + | ? | ? | - | **-** | **-** | + | + | + | + | **-** | **-** |
| Reis et al. (2004) | **x** | **-** | **-** | **-** | + | + | + | + | + | **-** | + | + | + | + | **-** | + |
| Angnes et al. (2005) ^Braz Oral Res^ | **x** | + | **-** | **-** | + | + | + | + | + | **-** | + | + | + | + | **?** | + |
| Burin et al. (2005) | **x** | **-** | **-** | **-** | + | + | + | + | + | **-** | + | + | + | + | **?** | + |
| Erten et al. (2005) | **x** | + | **-** | **-** | + | + | **-** | + | + | **-** | + | + | + | + | **-** | **?** |
| Maestriner et al. (2005) | **x** | + | **?** | **-** | + | + | **-** | + | **?** | **-** | + | + | + | + | **-** | **?** |
| Deery et al (2006) | **x** | **-** | + | **-** | + | + | + | + | + | **-** | + | + | + | + | **-** | + |
| Erten et al. (2006) | **x** | **-** | **?** | **-** | + | + | + | + | + | **?** | + | + | + | + | **-** | + |
| Reis et al. (2006) | **x** | + | **-** | **-** | + | + | + | + | + | **-** | + | + | + | + | **?** | + |
| Souza-Zaroni et al. (2006) | **x** | + | **?** | **-** | + | + | + | + | + | **-** | + | + | + | + | **-** | + |
| Ekstrand et al. (2007) | **x** | **?** | **-** | **-** | + | + | **-** | + | + | **-** | + | + | + | + | **-** | + |
| Manton et al. (2007) | **x** | + | **?** | **-** | + | **-** | **-** | + | **-** | **-** | + | + | + | + | **-** | **-** |
| Jablonski-Momeni et al. (2008) | **x** | **-** | **-** | **-** | + | + | + | + | + | **-** | + | + | + | + | + | + |
| Rodrigues et al. (2008) | **x** | + | **?** | **-** | + | + | - | + | + | **-** | + | + | + | + | + | + |
| Valera FB et al. (2008) | **x** | **-** | **-** | **-** | + | + | **-** | + | + | + | + | + | + | + | **?** | + |
| Diniz and Rodrigues (2009) | **x** | + | **-** | **-** | + | + | + | + | + | **-** | + | + | + | + | + | + |
| Kuehnisch et al. (2009) | **x** | **-** | **-** | **-** | + | + | - | + | + | - | + | + | + | + | + | + |
| Pereira et al. (2009) | **x** | + | + | - | + | + | + | + | + | **-** | + | + | + | + | **?** | **?** |
| Pourhashemi et al. (2009) | **x** | **-** | **-** | **-** | + | + | + | + | + | + | + | + | + | + | **?** | + |
| Rodrigues et al. (2009) | **x** | **-** | **?** | **-** | + | + | **-** | + | + | **-** | + | + | + | + | **?** | + |
| Sridhar et al. (2009) | **x** | **-** | **?** | **-** | + | + | **-** | + | + | **-** | + | + | + | + | **-** | **-** |
| De Paula et al. (2011) | **x** | + | **-** | **-** | + | + | **-** | + | + | **-** | + | + | + | + | **?** | + |
| Diniz et al. (2011) | **x** | **-** | + | **-** | + | + | + | + | + | **-** | + | + | + | + | **?** | + |
| Jablonski-Momeni et al. (2011) | **x** | **-** | **-** | **-** | + | + | **-** | + | + | **-** | + | + | + | + | **?** | **?** |
| Boye et al. (2012) | **x** | **-** | + | **-** | + | + | + | + | + | + | + | + | + | + | **-** | + |
| Jablonski Momeni et al. (2012) | **x** | **?** | **-** | **-** | + | + | + | + | + | **-** | + | + | + | + | + | **-** |
| Mitropoulos et al. (2012) | **x** | **-** | **-** | **-** | + | + | + | + | + | **-** | + | + | + | + | **?** | + |
| Seremidi et al. (2012) | **x** | **-** | + | **-** | + | + | + | + | + | + | + | + | + | + | **?** | **?** |
| Achilleos et al. (2013) | **x** | **-** | **-** | **-** | + | + | + | + | + | **-** | + | + | + | + | + | + |
| Gomez et al. (2013) | **x** | **-** | - | **-** | + | + | **-** | + | + | **-** | + | + | + | + | + | **?** |
| Van Hilsen et al. (2013) | **x** | **?** | **-** | **-** | + | + | **-** | + | + | **-** | + | + | + | + | **?** | + |
| Arslan et al. (2014) | **x** | **-** | **-** | **-** | + | + | **-** | + | + | **-** | + | + | + | + | **-** | **?** |
| Patel et al. (2014) | **x** | **-** | **-** | **-** | + | + | + | + | + | **-** | + | + | + | + | **?** | **-** |
| Sisodia et al. (2014) | **x** | **-** | **-** | **-** | + | + | + | + | + | **-** | + | + | + | + | **?** | + |
| Alomari et al. (2015) | **x** | **-** | **+** | **-** | + | + | **-** | + | + | **+** | + | + | + | + | **?** | **?** |
| Buecher et al. (2015) | **x** | **+** | **+** | **-** | **+** | **+** | **-** | **+** | **+** | **-** | **+** | **+** | **+** | **+** | ? | **-** |
| Jallad et al. (2015) | **x** | **-** | **?** | **-** | **+** | **+** | **+** | **+** | **+** | **-** | **+** | **+** | **+** | **+** | **?** | **+** |
| Neuhaus et al. (2015) | **x** | **-** | **-** | **-** | **+** | **+** | **+** | **+** | **+** | **-** | **+** | **+** | **+** | **+** | **+** | **+** |
| Özkan et al. (2015) | **x** | **-** | **-** | **-** | **+** | **+** | **+** | **+** | **+** | **-** | **+** | **+** | **+** | **+** | **?** | **+** |
| Ozturk et al. (2015) | **x** | **+** | **+** | **-** | **+** | **+** | **+** | **+** | **+** | **+** | **+** | **+** | **+** | **+** | **?** | **+** |
| Qudeimat et al. (2015) | **x** | **+** | **+** | **-** | **+** | **+** | **+** | **+** | **+** | **-** | **+** | **+** | **+** | **+** | **?** | **+** |
| Braun et al. 2016 | **x** | **-** | **-** | **-** | **+** | **-** | **+** | **+** | **-** | **-** | **+** | **+** | **+** | **+** | **+** | **-** |
| Iranzo-Cortez et al. (2017) | **x** | - | **?** | - | + | + | + | + | + | - | + | + | + | + | ? | ? |
| **Legend**: +=Low risk of bias (Yes); -=High risk of bias (Probably No, No); ?=Unclear (No information, Incomplete reporting, Probably Yes) **x**=Question for in vivo study only! a-,b- | | | | | | | | | | | | | | | | |

## Table S8b Risk of bias assessment for visual examination of in vivo validation studies on occlusal surfaces

| Visual examination of in vivo validation studies on occlusal surfaces | Signaling questions | | | | | | | | | | | | | | | |
| --- | --- | --- | --- | --- | --- | --- | --- | --- | --- | --- | --- | --- | --- | --- | --- | --- |
|  | **Selection bias** | | | | **Index test bias** | | | **Reference test bias** | | | | **Verification bias** | | | **Outcome bias** | |
|  | Patient selection | Teeth selection | Caries Spectrum | Sample size | Test Criteria | Blinding bias | Calibration bias | Test Criteria | Blinding bias | Calibration bias | Incorporation bias | Partial ver. bias | Differential ver. bias | Bias in the Analysis | Validity bias | Reproducibility bias |
| Verdonshoot et al. (1992) | **-** | **-** | + | **-** | + | + | **-** | **?** | **-** | **-** | + | + | + | + | **-** | **-** |
| Y.L.Ie et al. 1995 | **-** | + | - | - | + | + | - | + | - | - | + | + | + | + | ? | - |
| Nyvad et al. (1999) | **-** | **?** | **-** | **-** | + | + | + | **-** | - | **-** | - | + | + | + | **-** | + |
| Lussi et al. (2001) | **-** | **-** | **-** | **-** | + | + | + | + | **?** | **-** | + | + | + | + | **-** | **?** |
| Sheehy et al. (2001) | **-** | + | **-** | **-** | + | **-** | **-** | + | **-** | **-** | **-** | + | + | + | **-** | **?** |
| Heinrich-Weltzien et al. (2002) | **-** | + | + | - | + | + | + | + | - | - | + | + | + | + | - | + |
| Anttonen et al. (2003) | **-** | **-** | **-** | **-** | + | + | + | **-** | + | + | + | + | + | + | **-** | ? |
| Astvaldsdotttir et al. (2004) | **-** | **?** | **-** | **-** | + | + | + | + | + | **-** | + | + | + | + | **-** | + |
| Traneus et al. (2004) | **-** | + | **-** | **-** | + | + | + | + | + | **-** | + | + | + | + | **-** | + |
| Wolwacz et al. (2004) | **-** | **-** | **?** | **-** | + | + | + | **-** | **-** | **-** | **?** | + | + | + | **-** | **-** |
| Angnes et al. (2005)^Car Res^ | **-** | **-** | + | **-** | + | **-** | + | + | ? | **-** | + | + | + | + | **-** | **-** |
| Akarsu et al. (2006) | **-** | + | **-** | **-** | + | + | **-** | + | + | **-** | + | + | + | + | **?** | **-** |
| Olmez et al. (2006) | **-** | - | + | **-** | + | **-** | **-** | + | + | **-** | + | + | + | + | **-** | + |
| Reis et al. (2006) | **-** | + | **-** | **-** | + | + | + | + | + | **-** | + | + | + | + | **?** | + |
| Costa et al. (2008) | **-** | **-** | + | **-** | + | + | - | + | + | **-** | + | + | + | + | **-** | + |
| Huth et al. (2008) | **-** | + | **-** | + | + | + | + | + | + | **-** | + | + | + | + | + | + |
| Diniz et al. (2009) | **-** | + | **?** | **-** | + | + | + | **-** | **-** | **-** | + | + | + | + | **-** | **-** |
| Rando-Meirelles et al. (2011) | **-** | + | **-** | + | **-** | + | + | **-** | **-** | **-** | **-** | + | + | + | **-** | **-** |
| Diniz et al. (2012) | **-** | **-** | **-** | **-** | + | + | + | + | + | **-** | + | + | + | + | + | **?** |
| Rechmann et al. (2012) | **-** | **-** | **-** | **-** | + | + | **-** | - | **-** | **?** | **-** | + | + | + | **?** | **-** |
| Alammari et al. (2013) | **-** | **-** | **?** | + | **-** | **-** | + | **-** | + | **-** | + | + | + | + | **-** | **-** |
| Bozdemir et al. (2013) | **-** | + | **-** | **-** | + | **?** | + | + | **?** | **-** | + | + | + | + | **?** | **?** |
| Oancea et al. (2013) | **-** | **-** | **-** | **-** | + | + | + | + | + | **-** | + | + | + | + | **-** | **?** |
| Sinanoglu et al. (2014) | **-** | **-** | **-** | **-** | + | + | + | + | + | - | + | + | + | + | **?** | + |
| Bahrololoomi et al. (2015) | **-** | + | **?** | **-** | + | + | + | + | + | **-** | + | + | + | + | **?** | **?** |
| Cotta et al. (2015) | **-** | + | **-** | **-** | + | + | + | + | + | + | + | + | + | + | **?** | **?** |
| Castilho et al. (2016) | **-** | **+** | **+** | **-** | **+** | **+** | **+** | **+** | **+** | **-** | **+** | **+** | **+** | **+** | **-** | **+** |
| Melo et al. (2017) | **-** | **-** | **+** | **-** | **+** | **-** | **-** | **+** | **-** | **-** | **+** | **+** | **+** | **+** | **?** | **?** |
| Legend: +=Low risk of bias *(Yes*); -=High risk of bias (*No*)? =Unclear (*No information, Incomplete reporting, Probably Yes, Probably No*) | | | | | | | | | | | | | | | | |

## Table S9a Risk of bias assessment for conventional bitewing radiography of in vitro validation studies on occlusal surfaces

| **Conventional bitewing of in vitro validation studies on occlusal surfaces** | **Signaling questions** | | | | | | | | | | | | | | | | |
| --- | --- | --- | --- | --- | --- | --- | --- | --- | --- | --- | --- | --- | --- | --- | --- | --- | --- |
|  | **Selection bias** | | | | **Index test bias** | | | **Reference test bias** | | | | **Verification bias** | | | | **Outcome bias** | |
|  | Patient selection | Teeth selection | Caries Spectrum | Sample size | Test Criteria | Blinding bias | Calibration bias | Test Criteria | Blinding bias | Calibration bias | Incorporation bias | | Partial ver. bias | Differential ver. bias | Bias in the Analysis | Validity bias | Reproducibility bias |
| Wenzel et al. (1990) | **x** | - | + | - | + | + | - | + | - | - | - | | + | + | + | - | - |
| Wenzel et al. (1991) | **x** | + | + | - | + | + | - | + | - | - | + | | + | + | + | - | - |
| Nytun et al. (1992) | **x** | + | + | - | + | + | - | + | + | - | + | | + | + | + | - | - |
| Wenzel and Fejerskov (1992) | **x** | + | + | - | + | - | - | + | - | - | - | | + | + | + | - | - |
| Wenzel et al. (1992) | **x** | + | + | - | ? | + | - | + | ? | - | + | | + | + | + | - | - |
| Ketley and Holt (1993) | **x** | + | + | - | + | + | + | + | + | - | + | | + | + | + | - | - |
| Lussi et al. (1993) | **x** | - | + | - | + | + | - | + | + | - | + | | + | + | + | - | + |
| ^*^Russel and Pitts (1993) | **x** | - | - | - | + | + | - | + | + | - | + | | + | + | + | - | ? |
| Verdonshoot et al. (1993) | **x** | + | + | - | + | + | - | + | ? | - | + | | + | + | + | ? | - |
| Espelid et al. (1994) | **x** | - | - | - | - | - | - | + | + | - | + | | + | + | + | + | - |
| Hintze et al. (1994) | **x** | - | - | - | - | + | - | + | - | - | + | | + | + | + | - | - |
| Wenzel et al. (1994) | **x** | + | - | - | - | - | - | + | + | - | + | | + | + | + | ? | + |
| Lazarchik et al. (1995) | **x** | - | - | - | - | - | - | + | + | - | + | | + | + | + | - | ? |
| Lussi et al. (1995) | **x** | + | - | - | + | - | - | + | + | - | + | | + | + | + | - | + |
| Ricketts et al (1995) ^a^ | **x** | + | - | - | + | - | - | + | - | - | + | | + | + | + | - | - |
| Ricketts et al. (1995) ^b^ | **x** | - | - | - | + | - | - | + | - | - | + | | + | + | + | - | - |
| Hintze et al. (1996) ^A^ | **x** | - | - | - | - | + | - | + | - | - | + | | + | + | + | - | - |
| Hintze et al. (1996) ^B^ | **x** | **-** | **-** | **-** | - | + | ? | + | ? | **-** | + | | + | + | + | **-** | **-** |
| Ekstrand et al. (1997) | **x** | - | - | - | + | - | + | + | + | - | + | | + | + | + | - | + |
| Gray and Paterson (1997) | **x** | - | - | - | + | - | - | + | - | - | + | | + | + | + | - | - |
| Ricketts et al. (1997) | **x** | - | ? | - | + | + | - | + | + | - | + | | + | + | + | - | - |
| Schneiderman et al. (1997) | **x** | **-** | **-** | **-** | **-** | - | ? | **-** | **-** | **-** | **?** | | **-** | + | + | **-** | **-** |
| Ashley et al. (1998) | **x** | - | - | - | + | ? | - | + | ? | - | + | | + | + | + | - | ? |
| Huysmans et al. (1998) | **x** | - | - | - | + | - | - | + | + | - | + | | + | + | + | ? | ? |
| Abreu Jr et al. (1999) | **x** | **-** | **-** | **-** | **-** | **-** | **-** | + | + | **-** | + | | + | + | + | **-** | + |
| Cortes et al. (2000) | **x** | + | - | **-** | + | + | + | + | + | **-** | + | | + | + | + | + | + |
| Shi et al. (2000) | **x** | **-** | **?** | **-** | + | + | - | + | **-** | **-** | + | | + | + | + | **?** | **?** |
| Costa et al. (2002) | **x** | **-** | **-** | **-** | + | + | **-** | + | **-** | **-** | + | | + | + | + | **-** | + |
| Grossman et al. (2002) | **x** | **-** | **?** | **-** | + | + | + | + | + | + | + | | + | + | + | **-** | **-** |
| Hintze et al. (2002) | **x** | **-** | **-** | **-** | **-** | **?** | **-** | + | **?** | **-** | + | | + | + | + | **-** | **-** |
| Lussi et al. (2003) | **x** | **?** | **-** | **-** | + | + | **-** | + | + | **-** | + | | + | + | + | **-** | **?** |
| Lizarelli et al. (2004) | **x** | - | - | **-** | + | **-** | **-** | - | **-** | **-** | + | | + | + | + | **-** | **-** |
| Reis et al. (2004) | **x** | **-** | **-** | **-** | + | + | + | + | + | **-** | + | | + | + | + | **-** | + |
| Burin et al. (2005) | **x** | **-** | **-** | **-** | + | + | + | + | + | **-** | + | | + | + | + | **?** | + |
| Mestriner et al. (2005) | **x** | + | **?** | **-** | + | + | **-** | + | **?** | **-** | + | | + | + | + | **-** | **?** |
| Rocha et al. (2005) | **x** | **?** | **-** | **-** | + | + | + | **-** | **?** | **-** | + | | + | + | + | **-** | **?** |
| Souza-Zaroni et al. (2006) | **x** | + | **?** | **-** | + | + | + | + | + | **-** | + | | + | + | + | **-** | + |
| Manton et al. (2007) | **x** | + | **?** | **-** | + | **-** | **-** | + | **-** | **-** | + | | + | + | + | **-** | **-** |
| Rodrigues et al. (2008) | **x** | + | **?** | **-** | + | + | - | + | + | **-** | + | | + | + | + | + | + |
| Valera FB et al. (2008) | **x** | **-** | **-** | **-** | + | + | **-** | + | + | + | + | | + | + | + | **?** | + |
| Kuehnisch et al. (2009) | **x** | **-** | **-** | **-** | + | + | + | + | + | - | + | | + | + | + | + | + |
| Kuehnisch et al. (2009) | **x** | **-** | **-** | **-** | + | + | + | + | + | - | + | | + | + | + | + | + |
| Pereiera et al. (2009) | **x** | + | + | **-** | + | + | + | + | + | **-** | + | | + | + | + | **?** | **?** |
| Pourhashemi et al. (2009) | **x** | **-** | **-** | **-** | + | + | + | + | + | + | + | | + | + | + | **?** | + |
| Sridhar et al. (2009) | **x** | **-** | **?** | **-** | + | + | **-** | + | + | **-** | + | | + | + | + | **-** | **-** |
| Diniz et al. (2011) | **x** | **-** | + | **-** | + | + | - | + | + | **-** | + | | + | + | + | **?** | + |
| Arslan et al. (2014) | **x** | **-** | **-** | **-** | + | + | **-** | + | + | **-** | + | | + | + | + | **-** | **?** |
| Bussaneli et al. (2015) | **x** | **-** | **-** | **-** | + | + | **-** | + | - | **-** | + | | + | + | + | **?** | + |
| Krzyzostaniak et al (2015) | **x** | **-** | **-** | **-** | + | + | **-** | + | + | **-** | + | | + | + | + | **-** | **-** |
| Özkan et al. (2015) | **x** | **-** | **-** | **-** | **+** | **+** | **+** | **+** | **+** | **-** | **+** | | **+** | **+** | **+** | **?** | **+** |
| **Legend**: +=Low risk of bias *(Yes*); -=High risk of bias (*Probably No*, *No*);?=Unclear (*No information, Incomplete reporting, Probably Yes*) **x**=*Question for in vivo study only! *-studies with occlusal and proximal surfaces* | | | | | | | | | | | | | | | | | |

## Table S9b Risk of bias assessment for conventional bitewing radiography of in vivo validation studies on occlusal surfaces

| **Conventional bitewing of in vivo validation studies on occlusal surfaces** | **Signaling questions** | | | | | | | | | | | | | | | |
| --- | --- | --- | --- | --- | --- | --- | --- | --- | --- | --- | --- | --- | --- | --- | --- | --- |
|  | **Selection bias** | | | | **Index test bias** | | | **Reference test bias** | | | | **Verification bias** | | | **Outcome bias** | |
|  | Patient selection | Teeth selection | Caries Spectrum | Sample size | Test Criteria | Blinding bias | Calibration bias | Test Criteria | Blinding bias | Calibration bias | Incorporation bias | Partial ver. bias | Differential ver. bias | Bias in the Analysis | Validity bias | Reproducibility bias |
| Verdonshoot et al. (1992) | **-** | **-** | + | **-** | + | + | **-** | **?** | **-** | **-** | + | + | + | + | **-** | **-** |
| Lussi et al. (1995) | **-** | + | **-** | **-** | + | + | **-** | + | + | **-** | + | + | + | + | **-** | **-** |
| Hintze et al. (1996) | **-** | **-** | **-** | **-** | - | + | + | + | + | **-** | + | + | + | + | **-** | **-** |
| Townsend et al. (2000) | **-** | **?** | **-** | **-** | - | + | - | **-** | + | **-** | + | + | + | + | **-** | + |
| Lussi et al. (2001) | **-** | **-** | **-** | **-** | + | + | + | + | **?** | **-** | + | + | + | + | **-** | **?** |
| Thomas et al. (2001) | **-** | + | **?** | **-** | + | + | **-** | + | + | **-** | + | + | + | + | **?** | **?** |
| Heinrich-Weltzien et al. (2002) | **-** | + | + | - | + | + | + | + | - | - | + | + | + | + | - | + |
| Astvaldsdotttir et al. (2004) | **-** | **?** | **-** | **-** | + | + | + | + | + | **-** | + | + | + | + | **-** | + |
| Traneus et al. (2004) | **-** | + | **-** | **-** | + | + | + | + | + | **-** | + | + | + | + | **-** | + |
| Wolwacz et al. (2004) | **-** | **-** | **?** | **-** | + | + | + | **-** | **-** | **-** | **?** | + | + | + | **-** | **-** |
| Angnes et al. (2005)^Car Res^ | **-** | **-** | + | **-** | + | **-** | + | + | ? | **-** | + | + | + | + | **-** | **-** |
| Akarsu et al. (2006) | **-** | + | **-** | **-** | + | + | **-** | + | + | **-** | + | + | + | + | **?** | **-** |
| Olmez et al. (2006) | **-** | - | + | **-** | + | + | **-** | + | + | **-** | + | + | + | + | **?** | + |
| Costa et al. (2008) | **-** | **-** | + | **-** | + | + | - | + | + | **-** | + | + | + | + | **-** | + |
| Rando-Meirelles et al. (2011) | **-** | + | **-** | + | **-** | - | + | **-** | **-** | **-** | **-** | + | + | + | **-** | **-** |
| Diniz et al. (2012) | **-** | **-** | **-** | **-** | + | - | + | + | + | **-** | + | + | + | + | + | **?** |
| Bahrololoomi et al. (2015) | **-** | + | **?** | **-** | + | + | + | + | + | **-** | + | + | + | + | **?** | **?** |
| **Legend**: +=Low risk of bias (Yes); -=High risk of bias (Probably No, No); ?=Unclear (No information, Incomplete reporting, Probably Yes) | | | | | | | | | | | | | | | | |

## Table S10a Risk of bias assessment for digital bitewing radiography of in vitro validation studies on occlusal surfaces

| **Digital bitewing of in vitro validation studies on occlusal surfaces** | **Signaling questions** | | | | | | | | | | | | | | | | |  |
| --- | --- | --- | --- | --- | --- | --- | --- | --- | --- | --- | --- | --- | --- | --- | --- | --- | --- | --- |
|  | **Selection bias** | | | | **Index test bias** | | | **Reference test bias** | | | | **Verification bias** | | | | **Outcome bias** | |  |
|  | Patient selection | Teeth selection | Caries Spectrum | Sample size | Test Criteria | Blinding bias | Calibration bias | Test Criteria | Blinding bias | Calibration bias | Incorporation bias | | Partial ver. bias | Differential ver. bias | Bias in the Analysis | Validity bias | Reproducibility bias |  |
| Wenzel et al. (1990) | **x** | - | + | - | + | + | - | + | - | - | - | | + | + | + | - | - |  |
| Wenzel et al. (1991) | **x** | + | + | - | + | + | - | + | - | - | + | | + | + | + | - | - |  |
| Wenzel and Fejerskov (1992) | **x** | + | + | - | + | + | - | + | - | - | - | | + | + | + | - | - |  |
| Wenzel et al. (1992) | **x** | + | + | - | ? | + | - | + | ? | - | + | | + | + | + | - | - |  |
| Hintze et al. (1994) | **x** | - | - | - | - | + | - | + | - | - | + | | + | + | + | - | - |  |
| Wenzel et al (1995) | **x** | **-** | ? | **-** | **-** | + | **-** | + | + | **-** | + | | + | + | + | - | **-** |  |
| Ashley et al. (1998) | **x** | - | - | - | + | ? | - | + | ? | - | + | | + | + | + | - | ? |  |
| Abreu Jr et al. (1999) | **x** | **-** | **-** | **-** | **-** | **-** | - | + | + | **-** | + | | + | + | + | **-** | - |  |
| Costa et al. (2002) | **x** | **-** | **-** | **-** | + | + | **-** | + | **-** | **-** | + | | + | + | + | **-** | + |  |
| Hintze et al. (2002) | **x** | **-** | **-** | **-** | **-** | **?** | **-** | + | **?** | + | + | | + | + | + | **-** | **-** |  |
| Mestriner et al. (2005) | **x** | + | **?** | **-** | + | + | **-** | + | **?** | **-** | + | | + | + | + | **-** | **?** |  |
| Rocha et al. (2005) | **x** | **?** | **-** | **-** | + | + | + | **-** | **?** | **-** | + | | + | + | + | **-** | **?** |  |
| Kuehnisch et al. (2009) | **x** | **-** | **-** | **-** | + | + | + | + | + | - | + | | + | + | + | + | + |  |
| Jablonski Momeni et al. (2012) | **x** | **?** | **-** | **-** | + | + | - | + | + | **-** | + | | + | + | + | + | **-** |  |
| Buecher et al. (2015) | **x** | **+** | **+** | **-** | **+** | **+** | **-** | **+** | **+** | **-** | **+** | | **+** | **+** | **+** | **?** | **-** |  |
| Krzyzostaniak et al. (2015) | **x** | **-** | **-** | **-** | + | + | **-** | + | + | **-** | + | | + | + | + | **-** | **-** |  |
| Ozturk et al. (2015) | **x** | **+** | **+** | **-** | **+** | **+** | **+** | **+** | **+** | **+** | **+** | | **+** | **+** | **+** | **?** | **+** |  |
| Braun et al. (2016) | **x** | **-** | **-** | **-** | **+** | **-** | **-** | **+** | + | **-** | **+** | | **+** | **+** | **+** | **+** | **-** |  |
| **Legend**: +=Low risk of bias (Yes); -=High risk of bias (Probably No, No); ?=Unclear (No information, Incomplete reporting, Probably Yes) **x**=Question for in vivo study only! *-studies with occlusal and proximal surfaces | | | | | | | | | | | | | | | | | | |

## Table S10b Risk of bias assessment for digital bitewing radiography of in vivo validation studies on occlusal surfaces

| **Digital bitewing of in vivo validation studies on occlusal surfaces** | **Signaling questions** | | | | | | | | | | | | | | | | |
| --- | --- | --- | --- | --- | --- | --- | --- | --- | --- | --- | --- | --- | --- | --- | --- | --- | --- |
|  | **Selection bias** | | | | **Index test bias** | | | **Reference test bias** | | | | **Verification bias** | | | | **Outcome bias** | |
|  | Patient selection | Teeth selection | Caries Spectrum | Sample size | Test Criteria | Blinding bias | Calibration bias | Test Criteria | Blinding bias | Calibration bias | Incorporation bias | | Partial ver. bias | Differential ver. bias | Bias in the Analysis | Validity bias | Reproducibility bias |
| Huth et al. (2008) | **-** | + | **-** | + | + | + | + | + | + | **-** | + | | + | + | + | + | + |
| Rechmann et al. (2012) | **-** | **-** | **-** | **-** | + | - | **-** | - | **-** | **?** | **-** | | + | + | + | **?** | **-** |
| Sinanoglu et al. (2014) | **-** | **-** | **-** | **-** | + | + | + | + | + | - | + | | + | + | + | **?** | + |
|  |  |  |  |  |  |  |  |  |  |  |  | |  |  |  |  |  |
| **Legend**: +=Low risk of bias *(Yes*); -=High risk of bias (*Probably No*, *No*); ?=Unclear (*No information, Incomplete reporting, Probably Yes*) | | | | | | | | | | | | | | | | | |

## Table S11a Risk of bias assessment for laser fluorescence measurements of in vitro validation studies on occlusal surfaces

| **Laser Fluorescence measurements of in vitro validation studies on occlusal surfaces** | **Signaling questions** | | | | | | | | | | | | | | | |
| --- | --- | --- | --- | --- | --- | --- | --- | --- | --- | --- | --- | --- | --- | --- | --- | --- |
|  | **Selection bias** | | | | **Index test bias** | | | **Reference test bias** | | | | **Verification bias** | | | **Outcome bias** | |
|  | Patient selection | Teeth selection | Caries Spectrum | Sample size | Test Criteria | Blinding bias | Calibration bias | Test Criteria | Blinding bias | Calibration bias | Incorporation bias | Partial ver. bias | Differential ver. bias | Bias in the Analysis | Validity bias | Reproducibility bias |
| **DIAGNOdent 2095** |  |  |  |  |  |  |  |  |  |  |  |  |  |  |  |  |
| Ferreira Zandona et al. (1998) | **x** | - | - | - | + | + | + | + | + | - | + | + | + | + | - | + |
| Lussi et al. (1999) | **x** | + | **-** | **-** | + | + | - | + | + | **-** | + | + | + | + | **-** | + |
| Ando et al. (2000) | **x** | **-** | **-** | **-** | + | + | + | **?** | **?** | **-** | + | + | + | + | **-** | + |
| Shi et al. (2000) | **x** | **-** | **?** | **-** | + | + | + | + | **-** | **-** | + | + | + | + | **?** | **?** |
| Pereira et al. (2001) | **x** | + | **-** | **-** | + | + | + | + | + | **-** | + | + | + | + | **?** | + |
| El-Housseiny et al. (2001) | **x** | **-** | **-** | **-** | + | **-** | **-** | + | **-** | **-** | + | + | + | + | **-** | **?** |
| Ouellet et al. (2002) | **x** | + | **-** | **-** | + | **-** | **-** | + | **-** | **-** | + | + | + | + | **?** | **-** |
| Alwas-Danowska et al. (2002) | **x** | + | + | **-** | + | + | **-** | **-** | + | **-** | + | + | + | + | **?** | + |
| Costa et al. (2002) | **x** | **-** | **-** | **-** | + | + | + | + | **-** | **-** | + | + | + | + | **-** | + |
| Tonioli et al. (2002) | **x** | + | **-** | **-** | + | + | + | + | + | **-** | + | + | + | + | **?** | **-** |
| Bamzahim et al. (2002) | **x** | **-** | **?** | **-** | + | **?** | + | + | **?** | **-** | + | + | + | + | **-** | **?** |
| Francescut et al. (2003) | **x** | + | **-** | **-** | + | + | + | + | + | **-** | + | + | + | + | **-** | **?** |
| Kordic et al. (2003) | **x** | **-** | **?** | **-** | + | + | **-** | + | + | **-** | + | + | + | + | **?** | **?** |
| Cortes et al. (2003) | **x** | + | **?** | **-** | + | + | - | + | + | **-** | + | + | + | + | + | + |
| Lussi et al. (2003) | **x** | **?** | **-** | **-** | + | + | + | + | + | **-** | + | + | + | + | **-** | **?** |
| Baseren et al. (2003) | **x** | + | **?** | **-** | + | ? | + | + | + | **-** | + | + | + | + | **-** | + |
| Fung et al. (2004) | **x** | + | + | **-** | + | **-** | **+** | + | **-** | **-** | + | + | + | + | **-** | **?** |
| Lizarelli et al. (2004) | **x** | - | - | **-** | + | **-** | **-** | - | **-** | **-** | + | + | + | + | **-** | **-** |
| Reis et al. (2004) | **x** | **-** | **-** | **-** | + | + | + | + | + | **-** | + | + | + | + | **-** | + |
| Angnes et al. (2005) ^Braz Oral Res^ | **x** | + | **-** | **-** | + | + | + | + | + | **-** | + | + | + | + | **?** | + |
| Burin et al. (2005) | **x** | **-** | **-** | **-** | + | + | + | + | + | **-** | + | + | + | + | **?** | + |
| Deery et al. (2006) | **x** | **-** | + | **-** | + | + | + | + | + | **-** | + | + | + | + | **-** | + |
| Deery et al. (2006) | **x** | **-** | + | **-** | + | + | + | + | + | **-** | + | + | + | + | **-** | + |
| Lussi and Hellwig (2006) | **x** | + | **-** | **-** | + | - | + | + | + | **-** | + | + | + | + | **-** | **-** |
| Reis et al. (2006) | **x** | + | **-** | **-** | + | + | + | + | + | **-** | + | + | + | + | **?** | + |
| Souza-Zaroni et al. (2006) | **x** | + | **?** | **-** | + | + | + | + | + | **-** | + | + | + | + | **-** | + |
| Manton et al. (2007) | **x** | + | **?** | **-** | + | **-** | **-** | + | **-** | **-** | + | + | + | + | **-** | **-** |
| Rodrigues et al. (2008) | **x** | + | **?** | **-** | + | + | - | + | + | **-** | + | + | + | + | + | + |
| Valera FB et al. (2008) | **x** | **-** | **-** | **-** | + | + | **-** | + | + | + | + | + | + | + | **?** | + |
| Pereiera et al. (2009) | **x** | + | **?** | **-** | + | + | + | + | + | **-** | + | + | + | + | **?** | **?** |
| Sridhar et al. (2009) | **x** | **-** | **?** | **-** | + | + | + | + | + | **-** | + | + | + | + | **-** | **-** |
| Rodrigues et al. (2009) | **x** | **-** | **?** | **-** | + | + | + | + | + | **-** | + | + | + | + | **?** | + |
| Rodrigues and Hug (2009) | **x** | + | **-** | **-** | + | + | **-** | + | + | **-** | + | + | + | + | **?** | **?** |
| De Paula et al. (2011) | **x** | + | **-** | **-** | + | + | + | + | + | **-** | + | + | + | + | **?** | + |
| Jablonski-Momeni et al. (2011) | **x** | **-** | **-** | **-** | + | + | + | + | + | **-** | + | + | + | + | **?** | **?** |
| Rodrigues and Hug (2011) | **x** | + | **-** | **-** | + | + | **-** | + | + | **-** | + | + | + | + | **?** | + |
| Jablonski Momeni et al. (2012) | **x** | **?** | **-** | **-** | + | + | + | + | + | **-** | + | + | + | + | + | **-** |
| Oancea et al. (2013) | **x** | **-** | **-** | **-** | + | + | + | + | + | **-** | + | + | + | + | **-** | **?** |
|  |  |  |  |  |  |  |  |  |  |  |  |  |  |  |  |  |
| **DIAGNOdent Pen 2190** |  |  |  |  |  |  |  |  |  |  |  |  |  |  |  |  |
| Lussi and Hellwig (2006) | **x** | + | **-** | **-** | + | - | + | + | + | **-** | + | + | + | + | **-** | **-** |
| Rodrigues et al. (2008) | **x** | + | **?** | **-** | + | + | - | + | + | **-** | + | + | + | + | + | + |
| Rodrigues and Hug (2009) | **x** | + | **-** | **-** | + | + | **?** | + | + | **-** | + | + | + | + | **?** | **?** |
| Rodrigues and Hug (2011) | **x** | + | **-** | **-** | + | + | **-** | + | + | **-** | + | + | + | + | **?** | + |
| Aktan et al. (2012) | **x** | + | + | **-** | + | + | + | + | + | + | + | + | + | + | **?** | **?** |
| Seremidi et al. (2012) | **x** | **-** | + | **-** | + | + | + | + | + | + | + | + | + | + | **?** | **?** |
| Achilleos et al. (2013) | **x** | **-** | **-** | **-** | + | + | + | + | + | **-** | + | + | + | + | + | + |
| Arslan et al. (2014) | **x** | **-** | **-** | **-** | + | + | **-** | + | + | **-** | + | + | + | + | **-** | **?** |
| Mortensen et al. (2014) | **x** | + | **-** | **?** | + | + | + | + | + | **-** | + | + | + | + | **?** | + |
| Bussaneli et al. (2015) | **x** | **-** | **-** | **-** | + | + | **-** | + | - | **-** | + | + | + | + | **?** | + |
| Ozturk et al. (2015) | **x** | **+** | **+** | **-** | **+** | **+** | **+** | **+** | **+** | **+** | **+** | **+** | **+** | **+** | **?** | **+** |
| Iranzo-Cortez et al. (2017) | **x** | - | **?** | - | + | + | + | + | + | - | + | + | + | + | ? | ? |
| **Legend**: +=Low risk of bias (Yes); -=High risk of bias (Probably No, No); ?=Unclear (No information, Incomplete reporting, Probably Yes) **x**=Question for in vivo study only! | | | | | | | | | | | | | | | | |

## Table S11b Risk of bias assessment for laser fluorescence measurements of in vivo validation studies on occlusal surfaces

| **Laser Fluorescence measurements of in vivo validation studies on occlusal surfaces** | **Signaling questions** | | | | | | | | | | | | | | | | |
| --- | --- | --- | --- | --- | --- | --- | --- | --- | --- | --- | --- | --- | --- | --- | --- | --- | --- |
|  | **Selection bias** | | | | **Index test bias** | | | **Reference test bias** | | | | **Verification bias** | | | | **Outcome bias** | |
|  | Patient selection | Teeth selection | Caries Spectrum | Sample size | Test Criteria | Blinding bias | Calibration bias | Test Criteria | Blinding bias | Calibration bias | Incorporation bias | | Partial ver. bias | Differential ver. bias | Bias in the Analysis | Validity bias | Reproducibility bias |
| **DIAGNOdent 2095** |  |  |  |  |  |  |  |  |  |  |  | |  |  |  |  |  |
| Lussi et al. (2001) | **-** | **-** | **-** | **-** | + | + | + | + | **?** | **-** | + | | + | + | + | **-** | **?** |
| Sheehy et al. (2001) | **-** | + | **-** | **-** | + | **-** | **-** | + | **-** | **-** | **-** | | + | + | + | **-** | **?** |
| Heinrich-Weltzien et al. (2002) | **-** | + | + | **-** | + | + | + | + | - | **-** | + | | + | + | + | **-** | + |
| Alwas Danowska et al. (2002) | **-** | + | **-** | - | **?** | + | **-** | **?** | **?** | **-** | + | | + | + | + | **?** | + |
| Anttonen et al. (2003) | **-** | **-** | **-** | **-** | + | + | + | **-** | + | + | + | | + | + | + | **-** | ? |
| Astvaldsdotttir et al. (2004) | **-** | **?** | **-** | **-** | + | + | + | + | + | **-** | + | | + | + | + | **-** | + |
| Traneus et al. (2004) | **-** | + | **-** | **-** | + | + | + | + | + | **-** | + | | + | + | + | **-** | + |
| Angnes et al. (2005)^Car Res^ | **-** | **-** | + | **-** | + | **-** | + | **-** | ? | **-** | + | | + | + | + | **-** | **-** |
| Akarsu et al. (2006) | **-** | + | **-** | **-** | + | + | **-** | + | + | **-** | + | | + | + | + | **?** | **-** |
| Olmez et al. (2006) | **-** | - | + | **-** | + | + | **-** | + | + | **-** | + | | + | + | + | **?** | + |
| Reis et al. (2006) | **-** | + | **-** | **-** | + | + | + | + | + | **-** | + | | + | + | + | **-?** | + |
| Krause et al. (2007) | **-** | **-** | + | **-** | + | + | + | + | + | **-** | + | | + | + | + | **?** | **-** |
| Barberia et al. (2008) | **-** | + | **-** | **-** | + | + | - | + | + | **-** | + | | + | + | + | **?** | **-** |
| Costa et al. (2008) | **-** | **-** | + | **-** | + | + | + | + | + | **-** | + | | + | + | + | **-** | + |
| Abalos et al. (2008) | **-** | + | + | **-** | + | + | + | + | **-** | **-** | + | | + | + | + | **-** | **-** |
| Diniz et al. (2009) | **-** | + | **?** | **-** | + | + | + | **-** | **-** | **-** | + | | + | + | + | **-** | **-** |
| Rando-Meirelles et al. (2011) | **-** | + | **-** | + | **-** | - | + | **-** | **-** | **-** | **-** | | + | + | + | **-** | **-** |
| Abalos et al. (2012) | **-** | + | **-** | **-** | + | + | + | + | + | **-** | + | | + | + | + | **-** | **-** |
| Diniz et al. (2012) | **-** | **-** | **-** | **-** | + | + | + | + | + | **-** | + | | + | + | + | + | **?** |
| Rechmann et al. (2012) | **-** | **-** | **-** | **-** | + | + | **-** | - | **-** | **?** | **-** | | + | + | + | **?** | **-** |
| Bozdemir et al. (2013) | **-** | + | **-** | **-** | + | **?** | + | + | **?** | **-** | + | | + | + | + | **?** | **?** |
| Oancea et al. (2013) | **-** | **-** | **-** | **-** | + | + | + | + | + | **-** | + | | + | + | + | **-** | **?** |
| Sinanoglu et al. (2014) | **-** | **-** | **-** | **-** | + | + | + | + | + | - | + | | + | + | + | **?** | + |
| Castilho et al. (2016) | **-** | **+** | **+** | **-** | **+** | **+** | **+** | **+** | **+** | **-** | **+** | | **+** | **+** | **+** | **-** | **+** |
| Melo et al. (2017) | **-** | **-** | **+** | **-** | **+** | **-** | **+** | **+** | **-** | **-** | **+** | | **+** | **+** | **-** | **-** | **-** |
| **DIAGNOdent Pen 2190** |  |  |  |  |  |  |  |  |  |  |  | |  |  |  |  |  |
| Krause et al. (2007) | **-** | **-** | + | **-** | + | + | + | + | + | **-** | + | | + | + | + | **?** | **-** |
| Huth et al. (2008) | **-** | + | **-** | + | + | + | + | + | + | **-** | + | | + | + | + | + | + |
| Diniz et al. (2012) | **-** | **-** | **-** | **-** | + | + | + | + | + | **-** | + | | + | + | + | + | **?** |
| Bahrololoomi et al. (2015) | **-** | + | **?** | **-** | + | + | + | + | + | **-** | + | | + | + | + | **?** | **?** |
| **Legend**: +=Low risk of bias (Yes); -=High risk bias (Probably No, No); ?=Unclear ( No information, Incomplete reporting, Probably Yes) | | | | | | | | | | | | | | | | | |

## Table S12a Risk of bias assessment for FOTI of in vitro validation studies on occlusal surfaces

| **FOTI of in vitro validation studies on occlusal surfaces** | **Signaling questions** | | | | | | | | | | | | | | | | |
| --- | --- | --- | --- | --- | --- | --- | --- | --- | --- | --- | --- | --- | --- | --- | --- | --- | --- |
|  | **Selection bias** | | | | **Index test bias** | | | **Reference test bias** | | | | **Verification bias** | | | | **Outcome bias** | |
|  | Patient selection | Teeth selection | Caries Spectrum | Sample size | Test Criteria | Blinding bias | Calibration bias | Test Criteria | Blinding bias | Calibration bias | Incorporation bias | | Partial ver. bias | Differential ver. bias | Bias in the Analysis | Validity bias | Reproducibility bias |
| Wenzel et al. (1992) | **x** | + | - | - | **?** | + | - | + | ? | - | + | | + | + | + | - | - |
| Schneiderman et al. (1997) | **x** | **-** | **-** | **-** | **-** | - | **?** | **-** | **-** | **-** | **?** | | **-** | + | + | **-** | **-** |
| Ashley et al. (1998) | **x** | - | - | - | + | + | - | + | + | - | + | | + | + | + | - | ? |
| Cortes et al. (2000) | **x** | + | - | **-** | + | + | + | + | + | **-** | + | | + | + | + | + | + |
| Grossman et al. (2002) | **x** | **-** | + | **-** | + | - | + | + | + | + | + | | + | + | + | **?** | **-** |
| Cortes et al. (2003) | **x** | + | + | **-** | + | + | - | + | + | **-** | + | | + | + | + | + | + |
| Manton et al. (2007) | **x** | + | **?** | **-** | + | **-** | **-** | + | **-** | **-** | + | | + | + | + | **?** | **-** |
| Gomez et al. (2013) | **x** | **-** | - | **-** | + | + | **-** | + | + | **-** | + | | + | + | + | + | **?** |
| **Legend**: +=Low risk of bias *(Yes*); -=High risk of bias (*Probably No*, *No*); ?=Unclear (*No information, Incomplete reporting, Probably Yes*) **x**=*Question for in vivo study only!* | | | | | | | | | | | | | | | | | |

## Table S12b Risk of bias assessment for FOTI of in vivo validation studies on occlusal surfaces

| **FOTI of in vivo validation studies on occlusal surfaces** | **Signaling questions** | | | | | | | | | | | | | | | | |
| --- | --- | --- | --- | --- | --- | --- | --- | --- | --- | --- | --- | --- | --- | --- | --- | --- | --- |
|  | **Selection bias** | | | | **Index test bias** | | | **Reference test bias** | | | | **Verification bias** | | | | **Outcome bias** | |
|  | Patient selection | Teeth selection | Caries Spectrum | Sample size | Test Criteria | Blinding bias | Calibration bias | Test Criteria | Blinding bias | Calibration bias | Incorporation bias | | Partial ver. bias | Differential ver. bias | Bias in the Analysis | Validity bias | Reproducibility bias |
| Verdonshoot et al. (1992) | **-** | **-** | + | **-** | + | + | **-** | **?** | **-** | **-** | + | | + | + | + | **-** | **-** |
|  |  |  |  |  |  |  |  |  |  |  |  | |  |  |  |  |  |
| **Legend**: +=Low risk of bias *(Yes*); -=High risk of bias (*Probably No*, *No*); ?=Unclear (*No information, Incomplete reporting, Probably Yes*) | | | | | | | | | | | | | | | | | |

## Table S13a Risk of bias assessment for QLF of in vitro validation studies on occlusal surfaces

| **QLF of *i*n vitro validation studies on occlusal surfaces** | **Signaling questions** | | | | | | | | | | | | | | | | |
| --- | --- | --- | --- | --- | --- | --- | --- | --- | --- | --- | --- | --- | --- | --- | --- | --- | --- |
|  | **Selection bias** | | | | **Index test bias** | | | **Reference test bias** | | | | **Verification bias** | | | | **Outcome bias** | |
|  | Patient selection | Teeth selection | Caries Spectrum | Sample size | Test Criteria | Blinding bias | Calibration bias | Test Criteria | Blinding bias | Calibration bias | Incorporation bias | | Partial ver. bias | Differential ver. bias | Bias in the Analysis | Validity bias | Reproducibility bias |
| Ando et al. (2000) | **x** | **-** | **-** | **-** | + | + | + | **?** | **?** | **-** | + | | + | + | + | **-** | + |
| Kuehnisch et al. (2006) | **x** | + | + | **-** | + | + | + | + | + | **-** | + | | + | + | + | + | **-** |
| Pereiera et al. (2009) | **x** | + | **?** | **-** | + | + | + | + | + | **-** | + | | + | + | + | **?** | **?** |
| Gomez et al. (2013) | **x** | **-** | **-** | **-** | + | + | **-** | + | + | **-** | + | | + | + | + | + | **?** |
| Bussaneli et al. (2015) | **x** | **-** | **-** | **-** | + | + | **-** | + | - | **-** | + | | + | + | + | **?** | + |
| Jallad et al. (2015) | **x** | **-** | **?** | **-** | **-** | + | + | + | + | **-** | + | | + | + | + | **?** | + |
| **Legend**: +=Low risk of bias *(Yes*); -=High risk of bias (*Probably No*, *No*); ?=Unclear (*No information, Incomplete reporting, Probably Yes*) **x**=*Question for in vivo study only!* | | | | | | | | | | | | | | | | | |

## Table S13b Risk of bias assessment for QLF of in vivo validation studies on occlusal surfaces

| **QLF of in vivo validation studies on occlusal surfaces** | **Signaling questions** | | | | | | | | | | | | | | | | |
| --- | --- | --- | --- | --- | --- | --- | --- | --- | --- | --- | --- | --- | --- | --- | --- | --- | --- |
|  | **Selection bias** | | | | **Index test bias** | | | **Reference test bias** | | | | **Verification bias** | | | | **Outcome bias** | |
|  | Patient selection | Teeth selection | Caries Spectrum | Sample size | Test Criteria | Blinding bias | Calibration bias | Test Criteria | Blinding bias | Calibration bias | Incorporation bias | | Partial ver. bias | Differential ver. bias | Bias in the Analysis | Validity bias | Reproducibility bias |
| Alammari et al. (2013) | **-** | **-** | **?** | + | **-** | **-** | + | **-** | + | **-** | + | | + | + | + | **-** | **-** |
| **Legend**: +=Low risk of bias *(Yes*); -=High risk of bias (*Probably No*, *No*); ?=Unclear (*No information, Incomplete reporting, Probably Yes*) | | | | | | | | | | | | | | | | | |

## Table S8c Finally included result- visual examination of in vitro validation studies on occlusal surfaces

| **Threshold** | **Studies** | **Visual criteria:** | **Histology criteria:** | **Cross-tabulation** | **SE** | **SP** | **PPV** | **NPV** | **Az** | **N** |
| --- | --- | --- | --- | --- | --- | --- | --- | --- | --- | --- |
| Caries detection level | Deery et al. (1995) | 3 | 1 | - | + | + | + | + | - | 3 |
|  | Pereira et al. (2001) | 3 | 1 | - | + | + | - | - | - |  |
|  | Tonioli et al. (2002) | 18 | 4 | - | - | - | - | - | + |  |
|  | Erten et al. (2005) ^E^ | 14 | 10 | - | + | + | + | + | - |  |
|  | Souza Zaroni et al. (2006) | 18 | 16 | - | + | + | + | + | - |  |
|  | De Paula et al. (2011) | 6 | 4 | - | + | + | - | - | + |  |
| Dentine caries detection level | Nytun et al. (1992) | 6 | 4 | - | + | + | - | - | - | 8 |
|  | Lussi et al. (1993) | 6 | 3 | - | + | + | - | - | - |  |
|  | Deery et al. (1995) | 3 | 1 | - | + | + | + | + | - |  |
|  | Ricketts et al. (1995a) | 12 | 9 | + | + | + | + | + | + |  |
|  | Gray et al. (1997) | 6 | 4 | - | + | + | - | - | - |  |
|  | Cortes et al. (2000) | 17 | 12 | + | + | + | - | - | + |  |
|  | Pereira et al. (2001) | 3 | 1 | - | + | + | - | - | + |  |
|  | Mestriner et al. (2005) | 22 | 1 | - | + | + | - | - | + |  |
|  | Reis et al. (2006) ^E^ | 14 | 10 | - | + | + | - | - | - |  |
|  | De Paula et al. (2011) | 6 | 4 | - | + | + | - | - | + |  |
| Ekstrand outer 1/3 dentin | Angnes et al. (2005) ^E^ | 14 | 10 | - | + | + | - | - | + | 2 |
|  | Reis et al. (2006) ^E^ | 14 | 10 | - | + | + | - | - | - |  |

## Table S8d Final result- visual examination of in vivo validation studies on occlusal surfaces

| **Threshold** | **Studies** | **Visual criteria:** | **Histology criteria:** | **Cross-tabulation** | **SE** | **SP** | **PPV** | **NPV** | **Az** | **N** |
| --- | --- | --- | --- | --- | --- | --- | --- | --- | --- | --- |
| Caries detection level | Cotta et al. (2015) ^E^ | 24 | 10 | + | + | + | + | + | + | 2 |
|  | Castilho et al. (2016) ^E^ | 24 | 10 | + | + | + | + | + | + |  |
| Dentine caries detection level | Ie et al. (1995) | 17 | 7 | - | + | + | - | - | - | 0 |
|  | Reis et al. (2006) ^E^ | 14 | 10 | - | + | + | - | - | - |  |
| Ekstrand outer 1/3 dentin | Reis et al. (2006) ^E^ | 14 | 10 | - | + | + | - | - | - | 3 |
|  | Cotta et al. (2015) ^E^ | 24 | 10 | + | + | + | + | + | + |  |
|  | Castilho et al. (2016) ^E^ | 24 | 10 | + | + | + | + | + | + |  |

###

## Table S9c Final result- conventional bitewing radiography of in vitro validation studies on occlusal surfaces

| **Threshold** | **Studies** | **Visual criteria:** | **Histology criteria:** | **Cross-tabulation** | **SE** | **SP** | **PPV** | **NPV** | **Az** | **N** |
| --- | --- | --- | --- | --- | --- | --- | --- | --- | --- | --- |
| Caries detection level | / |  |  |  |  |  |  |  |  | 0 |
| Dentine caries detection level | Wenzel et al. (1991) | 4 | 3 | - | + | + | + | + | - | 5 |
|  | Nytun et al. (1992) | 7 | 5 | - | + | + | - | - | - |  |
|  | Ketley et al. (1993) | 2 | 2 | + | + | + | + | + | + |  |
|  | Lussi et al. (1995) | 6 | 3 | - | + | + | - | - | - |  |
|  | Cortes et al. (2000) | 8 | 12 | - | + | + | - | - | + |  |
|  | Mestriner et al. (2005) | 10 | 1 | - | + | + | - | - | + |  |
|  | Manton et al. (2007) | 1 | 14 | + | + | + | + | + | + |  |
|  | Rodrigues et al.(2008) | 10 | 1 | + | + | + | + | + | + |  |
| Ekstrand outer 1/3 dentin | / |  |  |  |  |  |  |  |  | 0 |

## Table S9d Final result- conventional bitewing radiography of in vivo validation studies on occlusal surfaces

| **Threshold** | **Studies** | **Visual criteria:** | **Histology criteria:** | **Cross-tabulation** | **SE** | **SP** | **PPV** | **NPV** | **Az** | **N** |
| --- | --- | --- | --- | --- | --- | --- | --- | --- | --- | --- |
| Caries detection level | Akarsu et al. (2006) | 8 | 6 | + | + | + | + | + | + | 2 |
|  | Bahrololoomi et al. (2015) | 7 | 6 | + | + | + | + | + | + |  |
| Dentine caries detection level | Lussi et al. (1995) | 6 | 3 | - | + | + | - | - | - | 4 |
|  | H. Weltzien et al. (2002) | 7 | 2 | + | + | + | + | + | + |  |
|  | Akarsu et al. (2006) | 8 | 6 | + | + | + | + | + | + |  |
|  | Bahrololoomi et al. (2015) | 7 | 6 | + | + | + | + | + | + |  |
| Ekstrand outer 1/3 dentin | / |  |  |  |  |  |  |  |  | 0 |

## Table S10c Final result- digital bitewing radiography of in vitro validation studies on occlusal surfaces

| **Threshold** | **Studies** | **Visual criteria:** | **Histology criteria:** | **Cross-tabulation** | **SE** | **SP** | **PPV** | **NPV** | **Az** | **N** |
| --- | --- | --- | --- | --- | --- | --- | --- | --- | --- | --- |
| Caries detection level | / |  |  |  |  |  |  |  |  | 0 |
| Dentine caries detection level | Wenzel et al. (1991) | 4 | 3 | - | + | + | + | + | - | 2 |
|  | Mestriner et al. (2005) | 10 | 1 | - | + | + | - | - | + |  |
|  | Buecher et al. (2015) | 1 | 1 | - | + | + | + | + | + |  |
| Ekstrand outer 1/3 dentin | / |  |  |  |  |  |  |  |  | 0 |

## Table S10d Final result- digital bitewing radiography of in vivo validation studies on occlusal surfaces

| **Threshold** | **Studies** | **Visual criteria:** | **Histology criteria:** | **Cross-tabulation** | **SE** | **SP** | **PPV** | **NPV** | **Az** | **N** |
| --- | --- | --- | --- | --- | --- | --- | --- | --- | --- | --- |
| Caries detection level | / |  |  |  |  |  |  |  |  | 0 |
| Dentine caries detection level | / |  |  |  |  |  |  |  |  | 0 |
|  |  |  |  |  |  |  |  |  |  |  |
| Ekstrand outer 1/3 dentin | / |  |  |  |  |  |  |  |  | 0 |

## Table S11c Final result- laser fluorescence of in vitro validation studies on occlusal surfaces

| **Threshold** | **Studies** | **Visual criteria:** | **Histology criteria:** | **Cross-tabulation** | **SE** | **SP** | **PPV** | **NPV** | **Az** | **N** |  |
| --- | --- | --- | --- | --- | --- | --- | --- | --- | --- | --- | --- |
| Caries detection level | Francescut et al. (2003) | 27 | 1 | - | + | + | - | - | - | 6 |  |
|  | Cortes et al. (2003) | 14 | 14 | + | + | + | + | + | + |  |  |
|  | Baseren et al. (2003) | 27 | 5 | + | + | + | + | + | + |  |  |
|  | Lussi and H. et al. (2006) | 19 | 1 | - | + | + | - | - | - |  |  |
|  | Souza Zaroni et al. (2006) | 18 | 1 | - | + | + | + | + | - |  |  |
|  | De Paula et al. (2011) | 1 | 6 | - | + | + | - | - | + |  |  |
|  | Rodrigues et al. (2011) | 19 | 5 | - | + | + | - | - | + | 2 |  |
|  | ***Diagnodent Pen 2190*** |  |  |  |  |  |  |  |  |  |  |
|  | Lussi et al. (2006) | 19 | 1 | - | + | + | - | - | - |  |  |
|  | Rodrigues et al. (2011) | 19 | 5 | - | + | + | - | - | + |  |  |
|  | Aktan et al. (2012) | 19 | 4 | + | + | + | + | + | + |  |  |
|  | Mortensen et al. (2014) | 1 | 2 | - | + | + | - | - | + |  |  |
| Dentine caries detection level | Francescut et al. (2003) | 27 | 1 | - | + | + | - | - | - | 7 |  |
|  | Cortes et al. (2003) | 14 | 14 | + | + | + | + | + | + |  |  |
|  | Baseren et al. (2003) | 27 | 5 | + | + | + | + | + | + |  |  |
|  | Fung et al. (2004) | 27 | 3 | - | + | + | - | - | - |  |  |
|  | Lussi and H. et al. (2006) | 19 | 1 | - | + | + | - | - | - |  |  |
|  | Manton et al. (2007) | 27 | 14 | + | + | + | + | + | + |  |  |
|  | Rodrigues et al. (2009) | 19 | 1 | - | + | + | - | - | + |  |  |
|  | Rod and Hug et al. (2009) | 19 | 1 | - | + | + | - | - | + |  |  |
|  | De Paula et al. (2011) | 1 | 6 | - | + | + | - | - | + |  |  |
|  | Rodrigues et al. (2011) | 19 | 5 | - | + | + | - | - | + | 4 |  |
|  | ***Diagnodent Pen 2190*** |  |  |  |  |  |  |  |  |  |  |
|  | Lussi et al. (2006) | 19 | 1 | - | + | + | - | - | - |  |  |
|  | Rodrigues et al. (2008) | 19 | 1 | + | + | + | + | + | + |  |  |
|  | Rod and Hug et al. (2009) | 19 |  | - | + | + | - | - | + |  |  |
|  | Rodrigues et al. (2011) | 19 | 5 | - | + | + | - | - | + |  |  |
|  | Aktan et al. (2012) | 19 | 4 | + | + | + | + | + | + |  |  |
|  | Mortensen et al. (2014) | 1 | 2 | - | + | + | - | - | + |  |  |
| Ekstrand outer 1/3 dentin | / |  |  |  |  |  |  |  |  | 0 |  |

## Table S11d Final result- laser fluorescence of in vivo validation studies on occlusal surfaces

| **Threshold** | **Studies** | **Visual criteria:** | **Histology criteria:** | **Cross-tabulation** | **SE** | **SP** | **PPV** | **NPV** | **Az** | **N** |
| --- | --- | --- | --- | --- | --- | --- | --- | --- | --- | --- |
| Caries detection level | Akarsu et al. (2006) | 3 | 6 | + | + | + | + | + | + | 1 |
| Dentine caries detection level | H. Weltzien et al. (2002) | 24 | 2 | + | + | + | + | + | + | 2 |
|  | Akarsu et al. (2006) | 3 | 6 | + | + | + | + | + | + |  |
|  | Bozdemir et al. (2013) | 3 | 2 | + | + | + | + | + | + |  |
| Ekstrand outer 1/3 dentin | / |  |  |  |  |  |  |  |  | 0 |

## Table S12c Final result- FOTI of in vitro validation studies on occlusal surfaces

| **Threshold** | **Studies** | **Visual criteria:** | **Histology criteria:** | **Cross-tabulation** | **SE** | **SP** | **PPV** | **NPV** | **Az** | **N** |
| --- | --- | --- | --- | --- | --- | --- | --- | --- | --- | --- |
| Caries detection level | Cortes et al. (2003) | 5 | 14 | + | + | + | + | + | + | 1 |
| Dentine caries detection level | Cortes et al. (2000) | 4 | 14 | + | + | + | + | + | + | 2 |
|  | Cortes et al. (2003) | 5 | 14 | + | + | + | + | + | + |  |
|  | Manton et al. (2007) | 4 | 14 | + | + | + | + | + | + |  |
| Ekstrand outer 1/3 dentin | / |  |  |  |  |  |  |  |  | 0 |

## Table S12d Final result- FOTI of in vivo validation studies on occlusal surfaces

| **Threshold** | **Studies** | **Visual criteria:** | **Histology criteria:** | **Cross-tabulation** | **SE** | **SP** | **PPV** | **NPV** | **Az** | **N** |
| --- | --- | --- | --- | --- | --- | --- | --- | --- | --- | --- |
| Caries detection level | / |  |  |  |  |  |  |  |  | 0 |
| Dentine caries detection level | / |  |  |  |  |  |  |  |  | 0 |
|  |  |  |  |  |  |  |  |  |  |  |
| Ekstrand outer 1/3 dentin | / |  |  |  |  |  |  |  |  | 0 |

## Table S13c Final result- QLF of in vitro validation studies on occlusal surfaces

| **Threshold** | **Studies** | **Visual criteria:** | **Histology criteria:** | **Cross-tabulation** | **SE** | **SP** | **PPV** | **NPV** | **Az** | **N** |
| --- | --- | --- | --- | --- | --- | --- | --- | --- | --- | --- |
| Caries detection level | / |  |  |  |  |  |  |  |  | 0 |
| Dentine caries detection level | / |  |  |  |  |  |  |  |  | 0 |
| Ekstrand outer 1/3 dentin | / |  |  |  |  |  |  |  |  | 0 |

## Table S13d Final result- QLF of in vivo validation studies on occlusal surfaces

| **Threshold** | **Studies** | **Visual criteria:** | **Histology criteria:** | **Cross-tabulation** | **SE** | **SP** | **PPV** | **NPV** | **Az** | **N** |
| --- | --- | --- | --- | --- | --- | --- | --- | --- | --- | --- |
| Caries detection level | / |  |  |  |  |  |  |  |  | 0 |
| Dentine caries detection level | / |  |  |  |  |  |  |  |  | 0 |
|  |  |  |  |  |  |  |  |  |  |  |
| Ekstrand outer 1/3 dentin | / |  |  |  |  |  |  |  |  | 0 |

# Detailed results from the meta-analysis

## Table S1c Final result- visual examination of in vitro validation studies on occlusal surfaces

| **Studies selected in the 1^st^ round** | **Status in the 2^nd^ round of selection** | **Reason for exclusion in the 2^nd^ round** | **ITC** | **RTC** | **Sample size and caries distribution** | | | | **Cut-off** | **Cross-tabulation** | **SE**  **(%)** | **SP**  **(%)** | **Az** | **Status in the 3^th^ round of selection** |
| --- | --- | --- | --- | --- | --- | --- | --- | --- | --- | --- | --- | --- | --- | --- |
|  |  |  |  |  | **Total** | **S** | **E** | **D** |  |  |  |  |  |  |
| Nytun et al. (1992) | **+** | **/** | **6** | **4** | 30 | 0 | 7 | 23 | Dentin caries detection level | no | 72 | 41 | **-** | **+** |
| Ketley and Holt et al. (1993) | **-** | Unacceptable diagnostic/reference criteria | **10** | **2** |  | | | |  |  |  |  |  |  |
| Lussi et al. (1993) | **+** | **/** | **6** | **3** | 63 | 22 | 13 | 28 | Dentin caries detection level | no | 12 | 93 | **-** | **+** |
| Deery et al. (1995) | + | / | 3 | 1 | 112 | 3 | 48 | 61 | Caries detection level | no | 77 | 73 | **-** | + |
|  |  |  |  |  |  |  |  |  | Dentin caries detection level |  | 56 | 92 |  |  |
| Ricketts et al. (1995a) | + | / | 12 | 9 | 48 | 8 | 32 | 8 | Dentin caries detection level | yes | 48.7 | 89.6 |  | + |
| Gray and Paterson et al. (1997) | + | / | 6 | 4 | 35 | 6 | 19 | 10 | Dentin caries detection level | no | 55 | - | - | + |
| Cortes et al. (2000) | + | / | 17 | 12 | 59 | 6 | 34 | 19 | Dentin caries detection level | no | 42 | 98 | 0.83 | + |
| Pereira et al. (2001) | + | / | 3 | 1 | 101 | 24 | 41 | 35 | Caries detection level | no | 92 | 54 | - | -  Incomplete data in tables |
|  |  |  |  |  |  |  |  |  | Dentin caries detection level |  | 17/7 | 97/99 | 0.78/0.77 |  |
| Tonioli et al. (2002) | + | / | 18 | 4 | 29 | 23^s^ | 50^s^ | 35^s^ | Caries detection level | no | - | - | 0.68 | -  miss SE,SP |
| Cortes et al. (2003) | - | Unacceptable diagnostic/reference criteria | 14 | 14 |  | | | |  |  |  |  |  |  |
| Angnes et al. (2005) | + |  | 14 | 10 | 57/110 ^sites^ | 20^s^ | 74^s^ | 16^s^ | Caries detection level | no | 68.7/ | 85.1/9 |  | + |
|  |  |  |  |  |  |  |  |  | Ekstrand outer 1/3 dentin |  | 69 | 91 |  |  |
| Erten et al. (2005) E | + | / | 14 | 10 | 84 | 21 | 28 | 19 | Caries detection level | no | 26 | 87 | - | -  Discrepancied in numbers |
| Mestriner et al. (2005) | + | / | 22 | 1 | 38 | 5 | 21 | 12 | Dentin caries detection level | no | 75 | 55 | - | + |
| Reis et al. (2006) ^E^ | + | / | 14 | 10 | 57/110^sites^ | 20 | 24/50 | 14/2 | Ekstrand outer 1/3 dentin | no | 71 | 57 | - | + |
| Souza Zaroni et al. (2006) | + | / | 18 | 16 | 47/121^sites^ | / | / | / | Caries detection level | no | 42-61 | 69-93 | - | + |
| Manton et al. (2007) | - | Unacceptable diagnostic/reference criteria | 14 | 14 |  | | | |  |  |  |  |  |  |
| Rodrigues et al. (2008) | - | Unacceptable diagnostic/reference criteria | 24 | 1 |  | | | |  |  |  |  |  |  |
| Diniz and Rodrigues et al. (2009) | - | Unacceptable diagnostic/reference criteria | 18  24 | 10  1 |  | | | |  |  |  |  |  |  |
| Pereira et al. (2009) | - | Unacceptable diagnostic/reference criteria | 14 | 1 |  | | | |  |  |  |  |  |  |
| De Paula et al. (2011) | + | / | 6 | 4 | 26/64 ^sites^ | 8^sites^ | 38 | 18 | Caries detection level | no | 63 | 100 | 0,81 | + |
| Buecher et al. (2015) | - | Unacceptable diagnostic/reference criteria | 25 | 1 |  | | | |  |  |  |  |  |  |
| Ozturk et al. (2015) | - | Unacceptable diagnostic/reference criteria | 24 | 17 |  | | | |  |  |  |  |  |  |
| Qudeimat et al. (2015) | - | Unacceptable diagnostic/reference criteria | 24 | 2 |  | | | |  |  |  |  |  |  |

ITC-Index test criteria, RTC- Reference test criteria, N-Number of teeth(sites), S-Sound, E-Caries in enamel, D- Caries in dentin

## Table S1d Final result- visual examination of in vivo validation studies on occlusal surfaces

| **Studies selected in the 1^st^ round** | **Status in the 2^nd^ round of selection** | **Reason for exclusion in the 2^nd^ round** | **ITC** | **RTC** | **Sample size and caries distribution** | | | | **Cut-off** | **Cross-tabulation** | **SE**  **(%)** | **SP (%)** | **Az** | **Status in the 3^th^ round of selection** |
| --- | --- | --- | --- | --- | --- | --- | --- | --- | --- | --- | --- | --- | --- | --- |
|  |  |  |  |  | **N** | **S** | **E** | **D** |  |  |  |  |  |  |
| Ie et al. (1995) | + | / | 17 | 7 | 60 | / | / | / | Dentin caries detection level | no | 0.4 | 97 | 0,66 | -  No values for histology |
| Heinrich-Weltzien et al. (2002) | - | Unacceptable diagnostic/reference criteria | 14 | 2 |  | | | |  |  |  |  |  |  |
| Traneus et al. (2004) | - | Unacceptable diagnostic/reference criteria | 14 | 6 |  | | | |  |  |  |  |  |  |
| Akarsu et al. (2006) | - | Unacceptable diagnostic/reference criteria | 14 | 6 |  | | | |  |  |  |  |  |  |
| Reis et al. (2006) | + | / | 14 | 10 | 57/110^sites^ | 20^sites^ | 74 | 16 | Ekstrand outer 1/3 dentin | no | 72 | 84 | - | **+** |
| Huth et al. (2008) | - | Unacceptable diagnostic/reference criteria | 25 | 2 |  | | | |  |  |  |  |  |  |
| Bozdemir et al. (2013) | - | Unacceptable diagnostic/reference criteria | 14 | 2 |  | | | |  |  |  |  |  |  |
| Bahrololoomi et al. (2015) | - | Unacceptable diagnostic/reference criteria | 14 | 6 |  | | | |  |  |  |  |  |  |
| Cotta et al. (2015) | + | / | 24 | 10 | 49^sites^ | 9 | 33 | 7 | Caries detection level | yes | 71 | 44 | 0,57 | **+** |
|  |  |  |  |  |  |  |  |  | Ekstrand outer 1/3 dentin |  | 71 | 86 | 0,79 |  |
| Castilho et al. (2016) | + | / | 24 | 10 | 43^sites^ | 8 | 32 | 3 | Caries detection level | yes | 50 | 71 | 0,60 | **+** |
|  |  |  |  |  |  |  |  |  | Ekstrand outer 1/3 dentin |  | 82 | 100 | 0,91 |  |

ITC-Index test criteria, RTC- Reference test criteria, N-Number of teeth(sites), S-Sound, E-Caries in enamel, D- Caries in dentin

## Table S2c Final result- conventional radiographic examination of in vitro validation studies on occlusal surfaces

| **Studies selected in the 1^st^ round** | **Status in the 2^nd^ round of selection** | **Reason for exclusion in the 2^nd^ round** | **ITC** | **RTC** | **Sample size and caries distribution** | | | | **Film Type** | **Cut-off** | **Cross-tabulation** | **SE (%)** | **SP (%)** | **Az** | **Status in the 3^th^ round of selection** |
| --- | --- | --- | --- | --- | --- | --- | --- | --- | --- | --- | --- | --- | --- | --- | --- |
|  |  |  |  |  | **N** | **S** | **E** | **D** |  |  |  |  |  |  |  |
| Wenzel et al. (1991) | + | / | 4 | 3 | 81 |  |  |  |  | Dentin caries detection level | no | - | - | - | -  Miss SE,SP |
| Nytun et al. (1992) | + | / | 7 | 5 | 30 | 0 | 7 | 23 | nr | Dentin caries detection level | no | 66 | 50 | - | -  Odd numbers |
| Ketley and Holt et al. (1993) | + | / | 2 | 2 | 100 | 0 | 56 | 44 | F Speed | Dentin caries detection level | yes | 67 | 92 |  | **+** |
| Verdonshoot et al. (1993) | - | Unacceptable diagnostic/reference criteria | 5 | 3 |  | | | |  |  |  |  |  |  |  |
| Lussi et al. (1995) | + | / | 6 | 3 | 26 | 10 | 5 | 11 | D-Speed | Dentin caries detection level | no | 77 | 62 | - | **+** |
| Ricketts et al. (1995) | - | Unacceptable diagnostic/reference criteria | nr | 9 |  | | | |  |  |  |  |  |  |  |
| Cortes et al. (2000) | + | / | 8 | 12 | 59 | 6 | 34 | 19 | E-Speed | Dentin caries detection level | no | 32 | 100 | 0.87 | **+** |
| Mestriner et al. (2005) | + | / | 10 | 1 | 38 | 5 | 21 | 12 | Direkt digitsl,no film | Dentin caries detection level | no | 45 | 73 | - | **+** |
| Souza Zaroni et al. (2006) | - | Unacceptable diagnostic/reference criteria | 13 | 16 |  | | | |  |  |  |  |  |  |  |
| Manton et al. (2007) | + | / | 1 | 14 | 67 | / | / | / | D Speed | Dentin caries detection level | yes | 15 | 95 |  | **-**  Incomplete table |
| Rodrigues et al. (2008) | + | / | 10 | 1 | 119 | 8 | 56 | 55 | F Speed | Dentin caries detection level | yes | 34 | 97 | 0.71 | **+** |
| Pereira et al. (2009) | - | Unacceptable diagnostic/reference criteria | 1 | 11 |  | | | |  |  |  |  |  |  |  |

ITC-Index test criteria, RTC- Reference test criteria, N-Number of teeth(sites), S-Sound, E-Caries in enamel, D- Caries in dentin

## Table S2d Final result- conventional radiographic examination of in vivo validation studies on occlusal surfaces

| **Studies selected in the 1^st^ round** | **Status in the 2^nd^ round of selection** | **Reason for exclusion in the 2^nd^ round** | **ITC** | **RTC** | **Sample size and caries distribution** | | | | **Film Type** | **Cut-off** | **Cross-tabulation** | **SE (%)** | **SP (%)** | **Az** | **Status in the 3^th^ round of selection** |
| --- | --- | --- | --- | --- | --- | --- | --- | --- | --- | --- | --- | --- | --- | --- | --- |
|  |  |  |  |  | **N** | **S** | **E** | **D** |  |  |  |  |  |  |  |
| Lussi et al. (1995) | + | none | 10 | 2 | 26/41^sites^ | 10/19^s^ | 5/7^s^ | 11/15^s^ |  |  | no | 77 | 62 |  | **+** |
| Thomas et al. (2001) | - | Unacceptable diagnostic/reference criteria | 5 | nr |  | | | |  |  |  |  |  |  |  |
| Heinrich-Weltzien et al. (2002) | + | none | 7 | 2 | 248 | 0 | 24 | 224 | E  Speed | Dentin caries detection level | yes | 70 | 96 |  | **+** |
| Traneus et al. (2004) | - | Unacceptable diagnostic/reference criteria | 10 | 6 |  | | | |  |  |  |  |  |  |  |
| Akarsu et al. (2006) | + | none | 8 | 6 | 165 | 38 | 42 | 85 | D Speed | Caries detection level | yes | 89/71 | 91/89 |  | **+** |
|  |  |  |  |  |  |  |  |  |  | Dentin caries detection level |  | 96/98 | 62/60 |  |  |
| Huth et al. (2008) | + | none | 10 | 2 |  |  |  |  |  |  |  |  |  |  | -  Incomplete data |
| Bahrololoomi et al. (2015) | + | none | 7 | 6 | 109 | 7 | 64 | 38 | E  Speed | Caries detection level | yes | 80/81 | 86/100 |  | **+** |
|  |  |  |  |  |  |  |  |  |  | Dentin caries detection level |  | 63/55 | 97/97 |  |  |

ITC-Index test criteria, RTC- Reference test criteria, N-Number of teeth(sites), S-Sound, E-Caries in enamel, D- Caries in dentin

## Table S3c Final result- digital radiographic examination of in vitro validation studies on occlusal surfaces

| **Studies selected in the 1^st^ round** | **Status in the 2^nd^ round of selection** | **Reason for exclusion in the 2^nd^ round** | **ITC** | **RTC** | **Sample size and caries distribution** | | | | **Film Type** | **Cut-off** | **Cross-tabulation** | **SE (%)** | **SP**  **(%)** | **Az** | **Status in the 3^th^ round of selection** |
| --- | --- | --- | --- | --- | --- | --- | --- | --- | --- | --- | --- | --- | --- | --- | --- |
|  |  |  |  |  | **N** | **S** | **E** | **D** |  |  |  |  |  |  |  |
| Wenzel et al. (1991) | - | Unacceptable diagnostic/reference criteria |  |  |  |  |  |  |  |  |  |  |  |  |  |
| Wenzel et al. (1992) | - | Unacceptable diagnostic/reference criteria | 5 | 7 |  | | | |  |  |  |  |  |  |  |
| Mestriner et al. (2005) | + | none | 10 | 1 | 38 | 5 | 21 | 12 | Phosphor plates | **Dentin caries detection level** | no | 35 | 84 |  | **+** |
| Buecher et al. (2015) | + | none | 1 | 1 | 196 | 35 | 146 | 15 | Phosphor  plates | **Dentin caries detection level** | no | 59 | 99 | 0.79 | **+** |
| Ozturk et al. (2015) | - | Unacceptable diagnostic/reference criteria | 2 | 17 |  | | | |  |  |  |  |  |  |  |

ITC-Index test criteria, RTC- Reference test criteria, N-Number of teeth(sites), S-Sound, E-Caries in enamel, D- Caries in dentin

## Table S3d Final result- digital radiographic examination of in vivo validation studies on occlusal surfaces

| **Studies selected in the 1^st^ round** | **Status in the 2^nd^ round of selection** | **Reason for exclusion in the 2^nd^ round** | **ITC** | **RTC** | **Sample size**  **and caries distribution** | **Cut-off** | **Cross-tabulation** | **SE (%)** | **SP (%)** | **Az** | **Status in the 3^th^ round of selection** |
| --- | --- | --- | --- | --- | --- | --- | --- | --- | --- | --- | --- |
| No studies were identified | | | | | | | | | | | |

ITC-Index test criteria, RTC- Reference test criteria, N-Number of teeth(sites), S-Sound, E-Caries in enamel, D- Caries in dentin

## Table S4c Final result- laser fluorescence measurement of in vitro validation studies on occlusal surfaces

| **Studies selected in the 1^st^ round** | **Status in the 2^nd^ round of selection** | **Reason for exclusion in the 2^nd^ round** | **Visual criteria:** | **Histology criteria:** | **Sample size and caries distribution** | | | | | | | | | | **Cut-off** | **Cross-tabulation** | **SE (%)** | **SP (%)** | **Az** | **Status in the 3^th^ round of selection** |
| --- | --- | --- | --- | --- | --- | --- | --- | --- | --- | --- | --- | --- | --- | --- | --- | --- | --- | --- | --- | --- |
|  |  |  |  |  | **N** | | | | **S** | | | **E** | | **D** |  |  |  |  |  |  |
| Lussi et al. (1999) | - | Unacceptable diagnostic/reference criteria | 5 | 1 |  | | | | | | | | | |  |  |  |  |  |  |
| Pereira et al. (2001) | - | Unacceptable diagnostic/reference criteria | 9 | 10 |  | | | | | | | | | |  |  |  |  |  |  |
| Quellet et al. (2002) | - | Unacceptable diagnostic/reference criteria | 13 | 10 |  | | | | | | | | | |  |  |  |  |  |  |
| Tonioli et al. (2002) | - | Unacceptable diagnostic/reference criteria | 5 | 4 |  | | | | | | | | | |  |  |  |  |  |  |
| Francescut et al. (2003) | + | none | 27 | 1 | 95 | | | | / | | | / | | / | **Caries detection level** | no | 77 | 49 |  | + |
|  |  |  |  |  |  |  |  |  |  |  |  |  |  |  | **Dentin caries detection level** |  | 73 | 65 |  |  |
| Cortes et al. (2003) | + | none | 14 | 14 | 152^sites^ | | | | 34 | | | 80 | | 38 | **Caries detection level** | no | 73 | 85 | 0.84 | + |
|  |  |  |  |  |  |  |  |  |  |  |  |  |  |  | **Dentin caries detection level** |  | 84 | 67 | 0.81 |  |
| Baseren et al. (2003) | + | none | 27 | 5 | 35/ 4 lost | | | | 29 | | | 6 | | 6 | **Dentin caries detection level** | yes | 66/100 |  | 74 | + |
| Fung et al. (2004) | + | none | 27 | 3 | 25 | | | | / | | | / | | / | **Dentin caries detection level** | no | 44-67 | 81-94 |  | -  Incomplete data |
| Angnes et al. (2005) | - | Unacceptable diagnostic/reference criteria | 16 | 10 |  | | | | | | | | | |  |  |  |  |  |  |
| Lussi and Hellwig et al. (2006) | + | none | 19 | 1 | 119 | | | | 26 | | | 51 | | 42 | **Caries detection level** | no | 96 | 69 |  | + |
|  |  |  |  |  |  |  |  |  |  |  |  |  |  |  | **Dentin caries detection level** |  | 81 | 79 |  |  |
| Reis et al. (2006) | - | Unacceptable diagnostic/reference criteria | 17 | 10 |  | | | | | | | | | |  |  |  |  |  |  |
| Souza-Zaroni et al. (2006) | + | none | 18 | 1 | 47 | / | | | | / | | | / | | **Caries detection level** | no | 35-45 | 82-99 |  | + |
| Manton et al. (2007) | + | none | 27 | 14 | 67 | / | | | | / | | | / | | **Dentin caries detection level** | yes | 49 | 83 |  | -  Incomplete data |
| Rodrigues et al. (2008) | + | none | 19 | 1 | 119 | 8 | | | | 56 | | | 55 | | **Dentin caries detection level** | yes | 51 | 89 | 0.81 | + |
| Pereira et al. (2009) | - | Unacceptable diagnostic/reference criteria | 1 | 10 |  | | | | | | | | | |  |  |  |  |  |  |
| Rodrigues and Hug et al. (2009) | + | none | 19 | 1 |  | | | | | | | | | |  |  |  |  |  | -  No values for histology |
| De Paula et al. (2011) | + | none | 1 | 6 | 26 | | |  | | |  | |  | | **Caries detection level** | no | 72 | 100 | 0.86 | + |
|  |  |  |  |  |  |  |  |  |  |  |  |  |  |  | **Dentin caries detection level** |  |  |  |  |  |
| Rodrigues et al. (2011) | + | none | 19 | 5 | 97 | | | 17 | | | 53 | | 27 | | **Caries detection level** | no | 70 | 76.5 | 0.72 | + |
|  |  |  |  |  |  |  |  |  |  |  |  |  |  |  | **Dentin caries detection level** |  | 63 | 88.6 | 0.85 |  |
| ***Diagnodent Pen 2190*** | | | | | | | | | | | | | | | | | | | | |
| Lussi and Hellwig et al. (2006) | + | none | 19 | 1 | 119 | | | 26 | | | 51 | | 42 | | **Caries detection level** | no | 88/91 | 77/77 |  | + |
|  |  |  |  |  |  |  |  |  |  |  |  |  |  |  | **Dentin caries detection level** |  | 79/86 | 84/71 |  |  |
| Rodrigues et al. (2008) | + | none | 19 | 1 | 119 | | | 8 | | | 56 | | 55 | | **Dentin caries detection level** | yes | 78 | 56 | 0.79 | + |
| Rodrigues and Hug et al. (2009) | + | none | 19 | 1 | 119 | | | 8 | | | 56 | | 55 | | **Dentin caries detection level** | no | 87 | 56 | 0.76 | -  No values for histology |
| Rodrigues and Hug et al. (2011) | + | none | 19 | 5 | 97 | | 17 | | | | 53 | | 27 | | **Caries detection level** | no | 62.5 | 76.5 | 0.72 | + |
|  |  |  |  |  |  |  |  |  |  |  |  |  |  |  | **Dentin caries detection level** |  | 63 | 87.1 | 0.85 |  |
| Aktan et al. (2012) | + | none | 19 | 4 | 129^sites^ | | 54 | | | | 48 | | 27 | | **Caries detection level** | yes | 65/65 | 33/43 | 0.33/0.76 | -  Incomplete data |
|  |  |  |  |  |  |  |  |  |  |  |  |  |  |  | **Dentin caries detection level** |  | 33/43 | 49/60 | 0.69/0.75 |  |
| Mortensen et al. (2014) | + | none | 1 | 2 | 100 | | 1 | | | | 58 | | 41 | | **Caries detection level** | no | 0.8-47 | 54-96 | 0.52-0.56 | -  Incomplete data |
|  |  |  |  |  |  |  |  |  |  |  |  |  |  |  | **Dentin caries detection level** |  | 0.7-49 | 54-93 | 0.50-0.54 |  |
| Ozturk et al. (2015) | - | Unacceptable diagnostic/reference criteria | 23 | 17 |  | | | | | | | | | |  |  |  |  |  |  |

ITC-Index test criteria, RTC- Reference test criteria, N-Number of teeth(sites), S-Sound, E-Caries in enamel, D- Caries in dentin

## Table S4d Final result- laser fluorescence measurement of in vivo validation studies on occlusal surfaces

| **Studies selected in the 1^st^ round** | **Status in the 2^nd^ round of selection** | **Reason for exclusion in the 2^nd^ step** | **ITC** | **RTC** | **Sample size and caries distribution** | | | | **Cut-off** | **Cross-tabulation** | **SE (%)** | **SP (%)** | **Az** | **Status in the 3^th^ step of selection** |
| --- | --- | --- | --- | --- | --- | --- | --- | --- | --- | --- | --- | --- | --- | --- |
|  |  |  |  |  | **N** | **S** | **E** | **D** |  |  |  |  |  |  |
| Heinrich-Weltzien et al. (2002) | + | none | 24 | 2 | 248 | 0 | 24 | 224 | **Dentine caries detection level** | yes | 93 | 63 |  | + |
| Traneus et al. (2004) | - | Unacceptable diagnostic/reference criteria | 3 | 6 |  | | | |  |  |  |  |  |  |
| Akarsu et al. (2006) | + | none | 3 | 6 | 165 | 38 | 42 | 85 | **Caries detection level** | yes | 88 | 71 |  | + |
|  |  |  |  |  |  |  |  |  | **Dentine caries detection level** |  | 89 | 0.87 |  |  |
| Reis et al. (2006) | - | Unacceptable diagnostic/reference criteria | 17 | 10 |  | | | |  |  |  |  |  |  |
| Barberia et al. (2008) | - | Unacceptable diagnostic/reference criteria | 3 | 14 |  | | | |  |  |  |  |  |  |
| Abalos et al. (2008) | - | Unacceptable diagnostic/reference criteria | nr | 7 |  | | | |  |  |  |  |  |  |
| Abalos et al (2012) | - | Unacceptable diagnostic/reference criteria | 26 | 14 |  | | | |  |  |  |  |  |  |
| Bozdemir et al. (2013) | + | none | 3 | 2 | 156 | / | / | / | **Caries detection level** | yes | 95-97 | 58-60 | 0.90-0.92 | -  No numbers for histology |
|  |  |  |  |  |  |  |  |  | **Dentine caries detection level** |  | 74-81 | 76-81 | 0.82-0.85 |  |
| Castilho et al. (2016) | - | Unacceptable diagnostic/reference criteria | 3 | 10 |  | | | |  |  |  |  |  |  |
| ***Diagnodent Pen 2190*** | | | | | | | | | | | | | | |
| Huth et al. (2008) | - | Unacceptable diagnostic/reference criteria | nr | 2 |  | | | |  |  |  |  |  |  |
| Bahrololoomi et al. (2015) | - | Unacceptable diagnostic/reference criteria | nr | 6 |  | | | |  |  |  |  |  |  |

ITC-Index test criteria, RTC- Reference test criteria, N-Number of teeth(sites), S-Sound, E-Caries in enamel, D- Caries in dentin

## Table S5c Final result- FOTI of in vitro validation studies on occlusal surfaces

| **Studies selected in the 1^st^ round** | **Status in the 2^nd^ round of selection** | **Reason for exclusion in the 2^nd^ round** | **ITC** | **RTC** | **Sample size**  **and caries distribution** | | | | **Cut-off** | **Cross-tabulation** | **SE (%)** | **SP (%)** | **Az** | **Status in the 3^th^ round of selection** |
| --- | --- | --- | --- | --- | --- | --- | --- | --- | --- | --- | --- | --- | --- | --- |
|  |  |  |  |  | **N** | **S** | **E** | **D** |  |  |  |  |  |  |
| Cortes et al. (2000) | + | none | 4 | 14 | 59 | 6 | 34 | 19 | **Dentin caries detection level** | yes | 32 | 100 | 0.85 | **+** |
| Cortes et al. (2003) | + | none | 5 | 14 | 152^sites^ | 34 | 80 | 38 | **Caries detection level** | yes | 98 | 50 | 0.88 | **+** |
|  |  |  |  |  |  |  |  |  | **Dentin caries detection level** |  | 66 | 96 | 0.89 |  |
| Manton et al. (2007) | + | none | 4 | 14 | 67 | / | / | / | **Dentin caries detection level** | yes | / | / | / | **-**  No numbers for histology |

ITC-Index test criteria, RTC- Reference test criteria, N-Number of teeth(sites), S-Sound, E-Caries in enamel, D- Caries in dentin

## Table 5d Final result- FOTI of in vivo validation studies on occlusal surfaces

| **Studies selected in the 1^st^ round** | **Status in the 2^nd^ round of selection** | **Reason for exclusion in the 2^nd^ round** | **ITC** | **RTC** | **Sample size**  **and caries distribution** | **Cut-off** | **Cross-tabulation** | **SE (%)** | **SP (%)** | **Az** | **Status in the 3^th^ round of selection** |
| --- | --- | --- | --- | --- | --- | --- | --- | --- | --- | --- | --- |
| No studies were identified | | | | | | | | | | | |

ITC-Index test criteria, RTC- Reference test criteria, N-Number of teeth(sites), S-Sound, E-Caries in enamel, D- Caries in dentin

## Table S6c Final result- QLF of in vitro validation studies on occlusal surfaces

| **Studies selected in the 1^st^ round** | **Status in the 2^nd^ round of selection** | **Reason for exclusion in the 2^nd^ round** | **ITC** | **RTC** | **Sample size**  **and caries distribution** | **Cut-off** | **Cross-tabulation** | **SE (%)** | **SP (%)** | **Az** | **Status in the 3^th^ step of selection** |
| --- | --- | --- | --- | --- | --- | --- | --- | --- | --- | --- | --- |
| Kuehnisch et al. (2006) | - | Unacceptable diagnostic/reference criteria |  |  |  |  |  |  |  |  |  |
| Pereiera et al. (2009) | - | Unacceptable diagnostic/reference criteria |  |  |  |  |  |  |  |  |  |

ITC-Index test criteria, RTC- Reference test criteria, N-Number of teeth(sites), S-Sound, E-Caries in enamel, D- Caries in dentin

## Table S6d Final result- QLF of in vivo validation studies on occlusal surfaces

| **Studies selected in the 1^st^ step** | **Status in the 2^nd^ step of selection** | **Reason for exclusion in the 2^nd^ step** | **ITC** | **RTC** | **Sample size**  **and caries distribution** | **Cut-off** | **Cross-tabulation** | **SE (%)** | **SP (%)** | **Az** | **Status in the 3^th^ step of selection** |
| --- | --- | --- | --- | --- | --- | --- | --- | --- | --- | --- | --- |
| Kuehnisch et al. (2006) | - | Unacceptable diagnostic/reference criteria |  |  |  |  |  |  |  |  |  |
| Pereiera et al. (2009) | - | Unacceptable diagnostic/reference criteria |  |  |  |  |  |  |  |  |  |

ITC-Index test criteria, RTC- Reference test criteria, N-Number of teeth(sites), S-Sound, E-Caries in enamel, D- Caries in dentin

# SROC Curve and Forest Plots

## **Table S14 SROC for different caries diagnostic methods- in vitro validation studies on occlusal surfaces**

| SROC | In vitro | | |
| --- | --- | --- | --- |
|  | 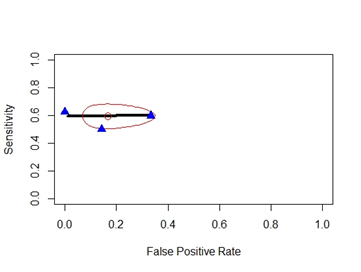Caries detection level | 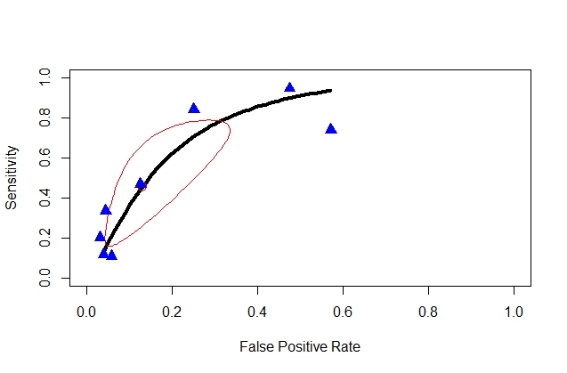Dentin detection level | 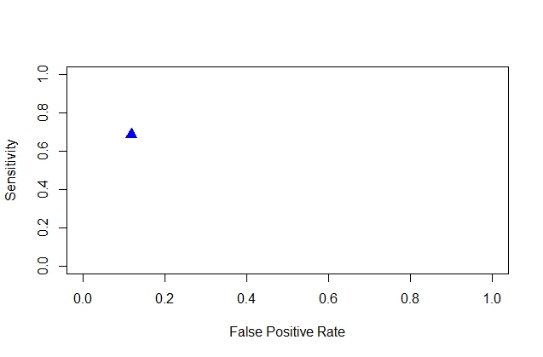1/3 dentine detection level |
| Visual examination |  | No. studies= 8  AUC= 0.79  Pooled Sen= 0.46 (0.20-0.73)  Pooled Spe= 0.87 (0.72-0.95)  Pooled DOR (REML)= 5.93 (3.11-11.31)  Heterogeneity chi-squared= 7.2152, df = 2, p-value = 0.3014  Inconsistency (I-squared)= 0%  Cochran's Q: 6.36 (7 df, p = 0.498)  Estimate of between-study variance (Tau-squared)= 0.00 | No. studies= 2  AUC= 0.89  Pooled Sen= 0.69 (0.51-0.82)  Pooled Spe= 0.88 (0.83-0.92)  Pooled DOR (REML)= 16.6 (4.85-56.79)  Heterogeneity chi-squared= 0.0044075, df = 2, p-value < 2.2e-16  Inconsistency (I-squared)= 0%  Cochran's Q: 0 (1 df, p = 1)  Estimate of between-study variance (Tau-squared)= 0.00 |
| Conventional bitewing radiography (D-Speed) | No Data Available  No. studies= 3  AUC= 0.59  Pooled Sen= 0.59 (0.52-0.67)  Pooled Spe= 0.83 (0.70-0.92)  Pooled DOR (REML)= 5.55 (1.88-16.38)  Heterogeneity chi-squared= 1.91, df = 2, p-value = 0.1667  Inconsistency (I-squared)= 0%  Cochran's Q: 1.802 (2 df, p = 0.406)  Estimate of between-study variance (Tau-squared)= 0.00 | 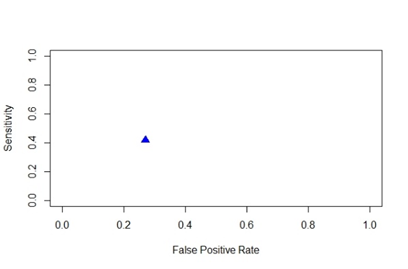  No. studies= 1  AUC= 0.60  Pooled Sen= 0.42 (0.18-0.69)  Pooled Spe= 0.73 (0.53-0.87)  Pooled DOR (REML)= 1.94 (0.46-8.17)  Heterogeneity chi-squared= NA  Inconsistency (I-squared)= NA  Cochran's Q: NA  Estimate of between-study variance (Tau-squared)= NA | No Data Available |

| SROC | 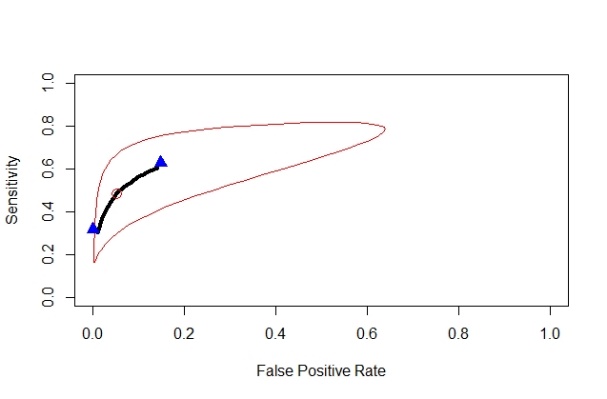In vitro | | |
| --- | --- | --- | --- |
|  | Caries detection level | Dentin detection level | 1/3 dentine detection level |
| Conventional bitewing radiography  (E-Speed) | No Data Available | No. studies= 2  AUC= 0.75  Pooled Sen= 0.48 (0.21-0.77)  Pooled Spe= 0.95 (0.53-0.997)  Pooled DOR (REML)= 10.69 (3.67-31.15)  Heterogeneity chi-squared= 0.10101, df = 2, p-value < 2.2e-16  Inconsistency (I-squared)= 0%  Cochran's Q: 0.856 (1 df, p = 0.355)  Estimate of between-study variance (Tau-squared)= 0.00 | No Data Available |
| Conventional  bitewing  radiography  (F-Speed) | No Data Available | 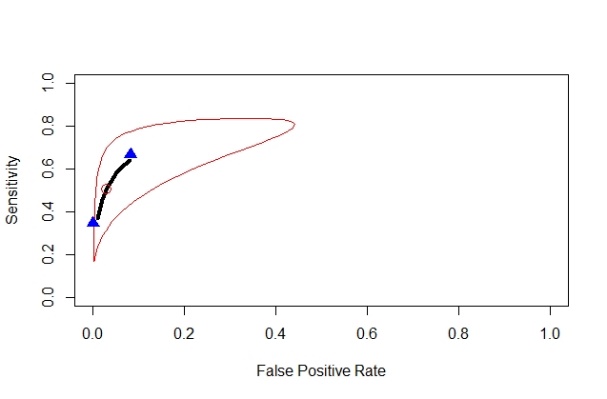  No. studies= 2  AUC= 0.82  Pooled Sen= 0.50 (0.22-0.79)  Pooled Spe= 0.97 (0.71-0.988)  Pooled DOR (REML)= 23.60 (8.28-67.24)  Heterogeneity chi-squared= 1.6631, df = 2, p-value < 2.2e-16  Inconsistency (I-squared)= 0%  Cochran's Q: 0.635 (1 df, p = 0.426)  Estimate of between-study variance (Tau-squared)= 0.00 | No Data Available |

| SROC | In vitro | | |
| --- | --- | --- | --- |
|  | Caries detection level | 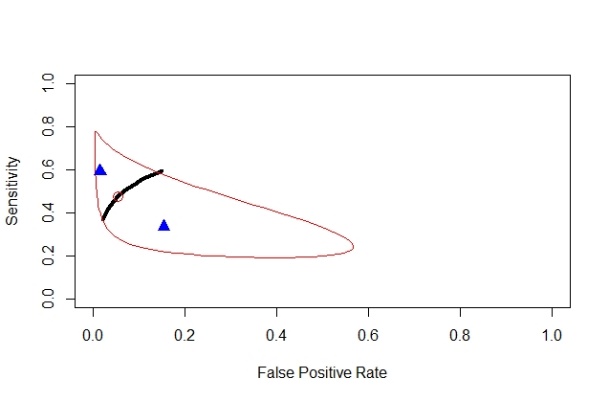Dentin detection level | 1/3 dentine detection level |
| Digital bitewing  radiography | No Data Available | No. studies= 2  AUC= 0.73  Pooled Sen= 0.48 (0.24-0.73)  Pooled Spe= 0.95 (0.59-0.995)  Pooled DOR (REML)= 15.57 (0.47-515.27)  Heterogeneity chi-squared= 4.0935, df = 2, p-value < 2.2e-16  Inconsistency (I-squared)= 0%  Cochran's Q: 1 (1 df, p = 0.317)  Estimate of between-study variance (Tau-squared)= 5.523 | No Data Available |
| Laser Fluorescence  2095 | 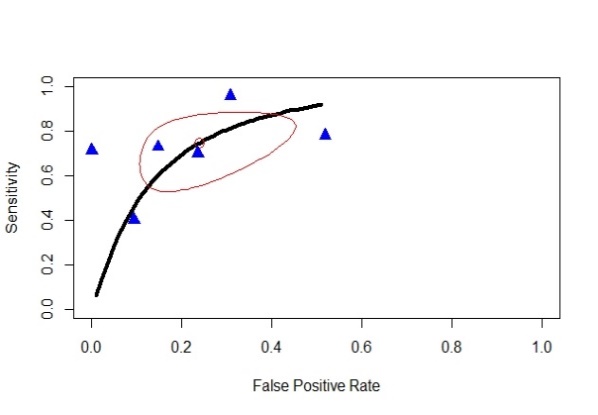  No. studies= 6  AUC= 0.81  Pooled Sen= 0.75 (0.58-0.86)  Pooled Spe= 0.76 (0.60-0.87)  Pooled DOR (REML)= 10.28 (4.35-24.28)  Heterogeneity chi-squared= 6.5763, df = 2, p-value = 0.16  Inconsistency (I-squared)= 0%  Cochran's Q: 4.648 (5 df, p = 0.46)  Estimate of between-study variance (Tau-squared)= 0.684 | 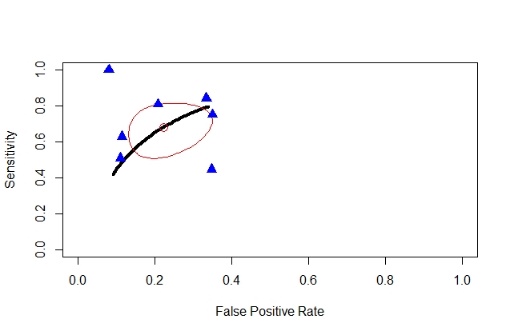  No. studies= 7  AUC= 0.79  Pooled Sen= 0.68 (0.54-0.79)  Pooled Spe= 0.78 (0.68-0.85)  Pooled DOR (REML)= 8.01 (4.04-15.88)  Heterogeneity chi-squared= 9.8203, df = 2, p-value = 0.08049  Inconsistency (I-squared)= 15.241%  Cochran's Q: 7.079 (6 df, p = 0.314)  Estimate of between-study variance (Tau-squared)= 0.484 | No Data Available |

| SROC | 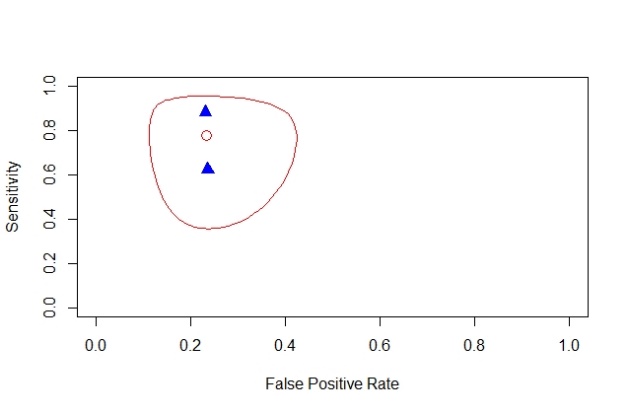In vitro | | |
| --- | --- | --- | --- |
|  | Caries detection level | Dentin detection level | 1/3 dentine detection level |
| Laser Fluorescence Pen 2190 | 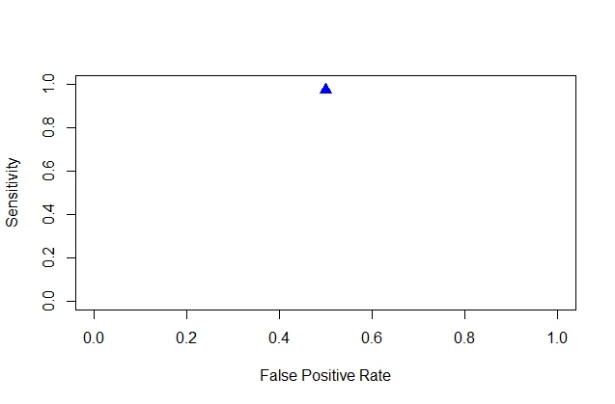  No. studies= 2  AUC= 0.77  Pooled Sen= 0.78 (0.44-0.94)  Pooled Spe= 0.77 (0.62-0.87)  Pooled DOR (REML)= 11.83 (2.66-52.63)  Heterogeneity chi-squared= 2.0404, df = 2, p-value < 2.2e-16  Inconsistency (I-squared)= 0%  Cochran's Q: 1 (1 df, p = 0.317)  Estimate of between-study variance (Tau-squared)= 0.81 | 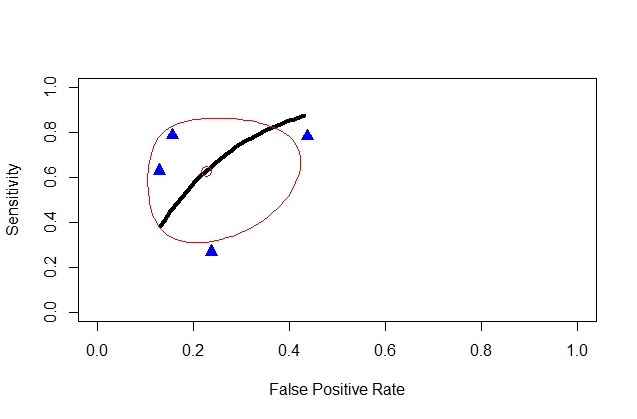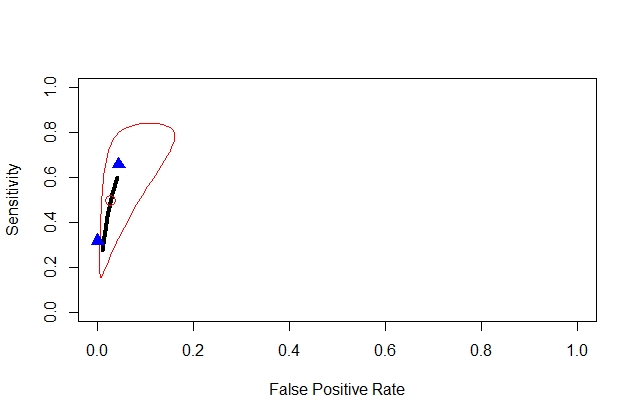  No. studies= 4  AUC= 0.78  Pooled Sen= 0.63 (0.37-0.83)  Pooled Spe= 0.77 (0.62-0.88)  Pooled DOR (REML)= 5.85 (1.77-19.30)  Heterogeneity chi-squared= 4.8237, df = 2, p-value = 0.08965  Inconsistency (I-squared)= 2.61%  Cochran's Q: 3.08 (3 df, p = 0.379)  Estimate of between-study variance (Tau-squared)= 1.255 | No Data Available |
| Fiber-optic  transillumination  FOTI | No. studies= 1  AUC= 0.92  Pooled Sen= 0.97 (0.92-0.99)  Pooled Spe= 0.50 (0.34-0.66)  Pooled DOR (REML)= 38.33 (10.15-144.77)  Heterogeneity chi-squared= NA  Inconsistency (I-squared)= NA  Cochran's Q: NA  Estimate of between-study variance (Tau-squared)= NA | No. studies= 2  AUC= 0.92  Pooled Sen= 0.49 (0.20-0.79)  Pooled Spe= 0.97 (0.89-0.994)  Pooled DOR (REML)= 37.77 (13.69-104.19)  Heterogeneity chi-squared= 1.3503, df = 2, p-value < 2.2e-16  Inconsistency (I-squared)= 0%  Cochran's Q: 0.001 (1 df, p = 0.982)  Estimate of between-study variance (Tau-squared)= 0.00 | No Data Available |

## **Table S15 SROC for different caries diagnostic methods- in vivo validation studies on occlusal surfaces**

| SROC | In vivo | | |
| --- | --- | --- | --- |
|  | Caries detection level | Dentin detection level | 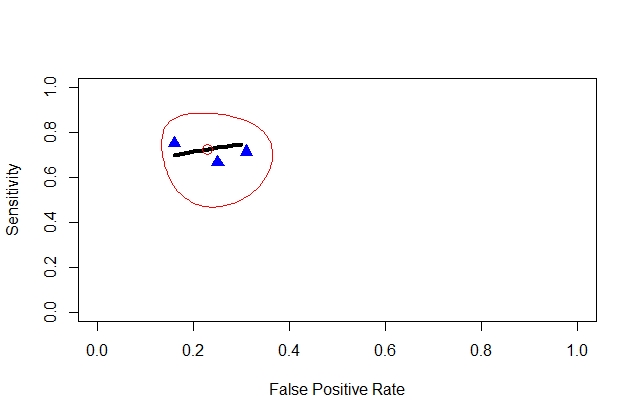1/3 dentine detection level |
| Visual examination | 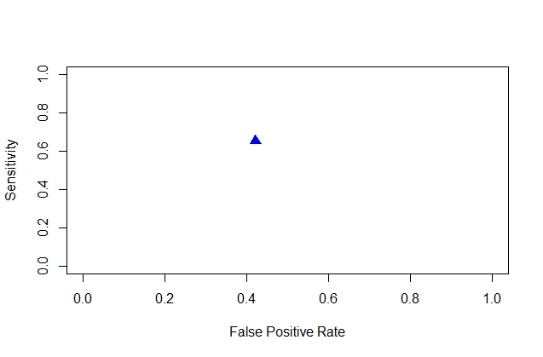  No. studies= 2  AUC= 0.70  Pooled Sen= 0.70 (0.59-0.80)  Pooled Spe= 0.47 (0.26-0.70)  Pooled DOR (REML)= 2.14 (0.73-6.28)  Heterogeneity chi-squared= 0.24182, df = 2, p-value < 2.2e-16  Inconsistency (I-squared)= 0%  Cochran's Q: 0.071 (1 df, p = 0.79)  Estimate of between-study variance (Tau-squared)= 0.00 | No Data Available | No. studies= 3  AUC= 0.77  Pooled Sen= 0.72 (0.52-0.86)  Pooled Spe= 0.77 (0.67-0.85)  Pooled DOR (REML)= 10.18 (3.94-26.29)  Heterogeneity chi-squared= 0.63131, df = 2, p-value = 0.4269  Inconsistency (I-squared)= 0%  Cochran's Q: 1.086 (2 df, p = 0.581)  Estimate of between-study variance (Tau-squared)= 0.00 |
| Conventional bitewing  (D-Speed) | 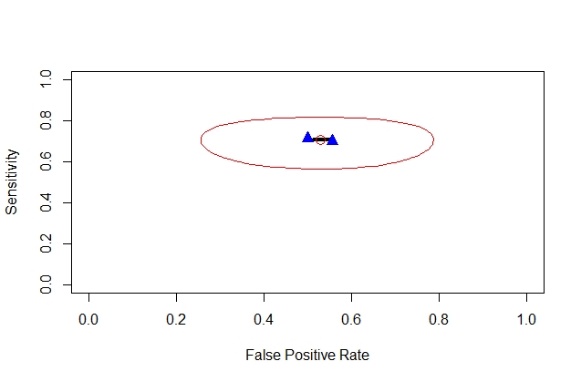  No. studies= 1  AUC= 0.65  Pooled Sen= 0.65 (0.57-0.73)  Pooled Spe= 0.58 (0.42-0.72)  Pooled DOR (REML)= 2.59 (1.24-5.44)  Heterogeneity chi-squared= NA  Inconsistency (I-squared)= NA  Cochran's Q: NA  Estimate of between-study variance (Tau-squared)= NA | 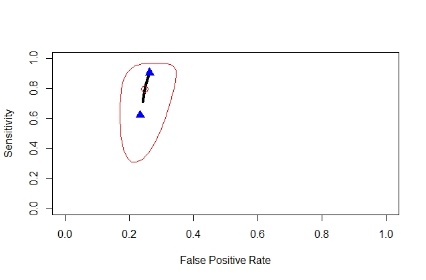  No. studies= 2  AUC= 0.77  Pooled Sen= 0.79 (0.41-0.96)  Pooled Spe= 0.75 (0.68-0.82)  Pooled DOR (REML)= 11.79 (2.43-57.24)  Heterogeneity chi-squared= 2.0263, df = 2, p-value < 2.2e-16  Inconsistency (I-squared)= 0%  Cochran's Q: 1 (1 df, p = 0.317)  Estimate of between-study variance (Tau-squared)= 1.137 | No Data Available |

| SROC | In vivo | | |
| --- | --- | --- | --- |
|  | Caries detection level | Dentin detection level | 1/3 dentine detection level |
| Conventional bitewing radiography  (E-Speed) | 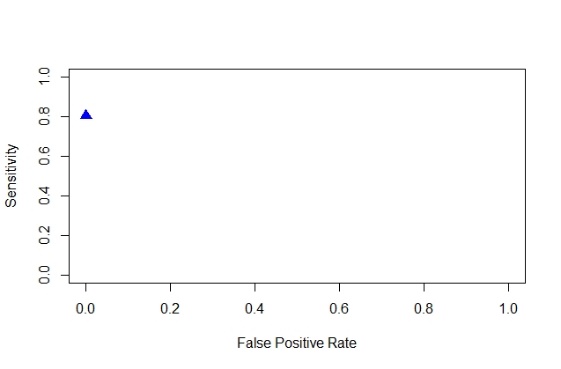  No. studies= 1  AUC= 0.94  Pooled Sen= 0.80 (0.71-0.87)  Pooled Spe= 0.94 (0.46-0.996)  Pooled DOR (REML)= 60.37 (3.31-1100.70)  Heterogeneity chi-squared= NA  Inconsistency (I-squared)= NA  Cochran's Q: NA  Estimate of between-study variance (Tau-squared)= NA | 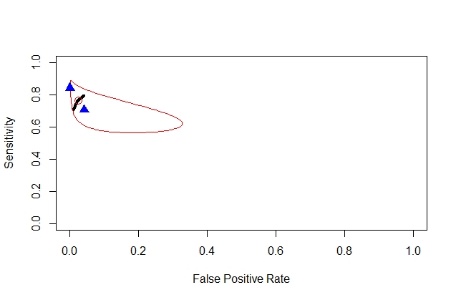  No. studies= 2  AUC= 0.90  Pooled Sen= 0.76 (0.61-0.87)  Pooled Spe= 0.98 (0.79-0.998)  Pooled DOR (REML)= 127.56 (7.38-2203.70)  Heterogeneity chi-squared= 2.0001, df = 2, p-value < 2.2e-16  Inconsistency (I-squared)= 0%  Cochran's Q: 1 (1 df, p = 0.317)  Estimate of between-study variance (Tau-squared)= 2.886 | No Data Available |
| Conventional bitewing radiography  (F-Speed) | No Data Available | No Data Available | No Data Available |

| SROC | In vivo | | |
| --- | --- | --- | --- |
|  | Caries detection level | Dentin detection level | 1/3 dentine detection level |
| Digital bitewing radiography | No Data Available | No Data Available | No Data Available |
| Laser Fluorescence 2095 | 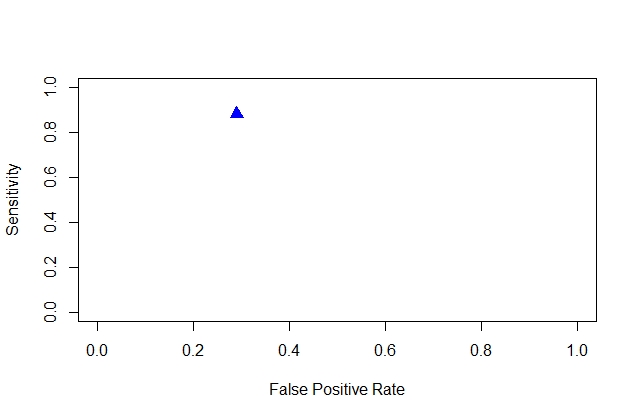  No. studies= 1  AUC= 0.88  Pooled Sen= 0.88 (0.81-0.93)  Pooled Spe= 0.71 (0.55-0.83)  Pooled DOR (REML)= 18.33 (7.57-44.37)  Heterogeneity chi-squared= NA  Inconsistency (I-squared)= NA  Cochran's Q: NA  Estimate of between-study variance (Tau-squared)= NA | 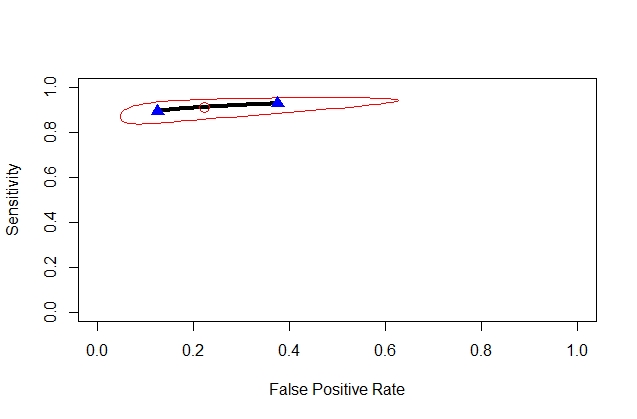  No. studies= 2  AUC= 0.92  Pooled Sen= 0.91 (0.86-0.95)  Pooled Spe= 0.78 (0.46-0.94)  Pooled DOR (REML)= 35.90 (13.43-96.00)  Heterogeneity chi-squared= 0.46001, df = 2, p-value < 2.2e-16  Inconsistency (I-squared)= 0%  Cochran's Q: 1 (1 df, p = 0.317)  Estimate of between-study variance (Tau-squared)= 0.262 | No Data Available |
| Laser Fluorescence 2190 | No Data Available | No Data Available | No Data Available |
| Fiber-optic transillumination  FOTI | No Data Available | No Data Available | No Data Available |

## **Table S16 Forest plots (DOR) for different caries diagnostic methods- in vitro validation studies on occlusal surfaces**

| Forest Plots | 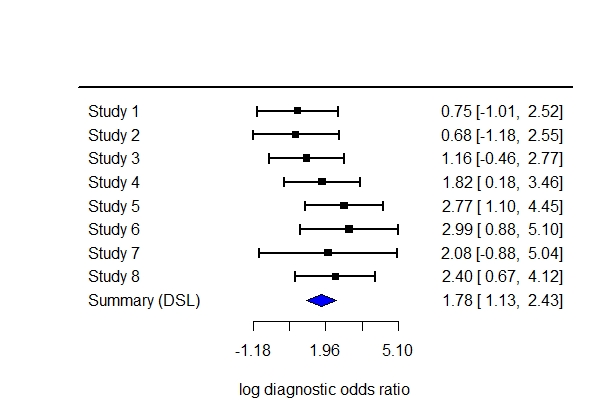In vitro | | |
| --- | --- | --- | --- |
|  | 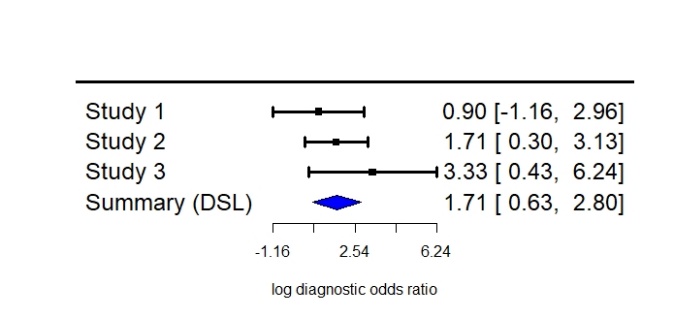Caries detection level | Dentin detection level | 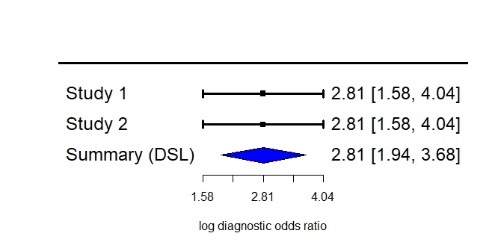1/3 dentin detection level |
| Visual examination | **Deery et al. (1995)**  **Souza Zaroni et al. (2006)**  **De Paula et al. (2011)**  **Summary** | **Nytun et al. (1992)**  **Lussi et al. (1993)**  **Deery et al. (1995)**  **Ricketts et al. (1995a)**  **Gray and Paterson et al. (1997)**  **Cortes et al. (2000)**  **Mestriner et al. (2005)**  **De Paula et al. (2011)**  **Summary** | **Angnes et al. (2005)**  **Reis et al. (2006)**  **Summary** |
| Conventional bitewing radiography (D -speed) | No Data Available | No Data Available | No Data Available |
| Conventional bitewing radiography  (E -speed) | No Data Available | 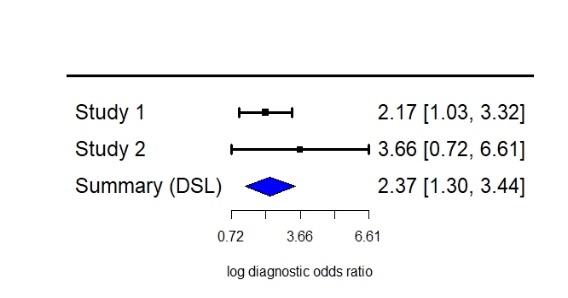  **Wenzel et al. (1991)**  **Cortes et al. (2000)**  **Summary** | No Data Available |

| Forest Plots | In vitro | | |
| --- | --- | --- | --- |
|  | Caries detection level | 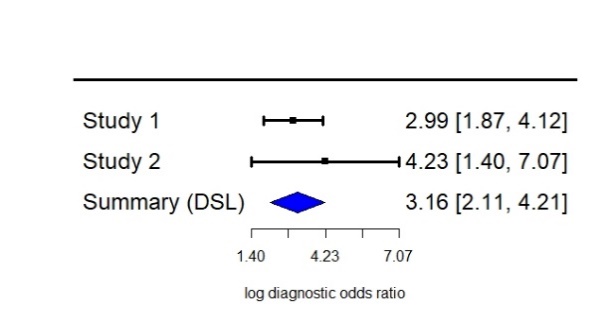Dentin detection level | 1/3 dentin detection level |
| Conventional bitewing radiography  (F -speed) | No Data Available | **Ketley and Holt et al. (1993)**  **Rodrigues et al. (2008)**  **Summary** | No Data Available |
| Digital bitewing radiography | 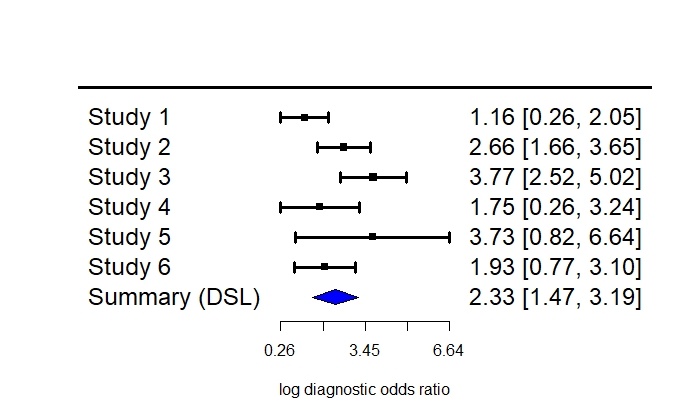No Data Available | 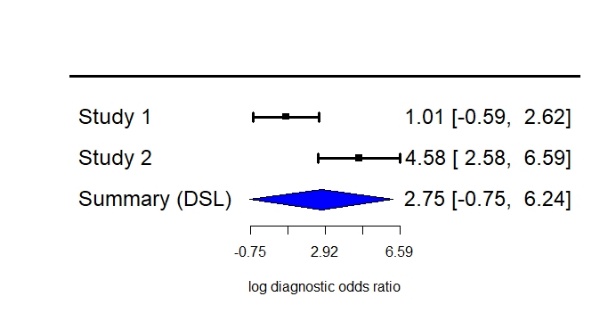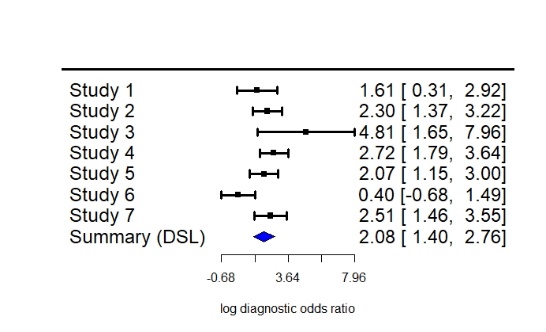  **Mestriner et al. (2005)**  **Buecher et al. (2015)**  **Summary** | No Data Available |
| Laser Fluorescence 2095 | **Francescut et al. (2003)**  **Cortes et al. (2003)**  **Lussi and Hellwig (2006)**  **Souza-Zaroni et al. (2006)**  **De Paula et al. (2011)**  **Rodrigues et al. (2011)**  **Summary** | **Francescut et al. (2003)**  **Cortes et al. (2003)**  **Baseren et al. (2003)**  **Lussi and Hellwig (2006)**  **Rodrigues et al. (2008)**  **De Paula et al. (2011)**  **Rodrigues et al. (2011)**  **Summary** | No Data Available |

| Forest Plots | In vitro | | |
| --- | --- | --- | --- |
|  | 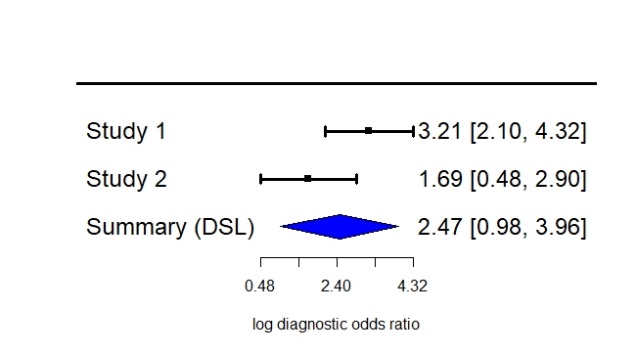Caries detection level | 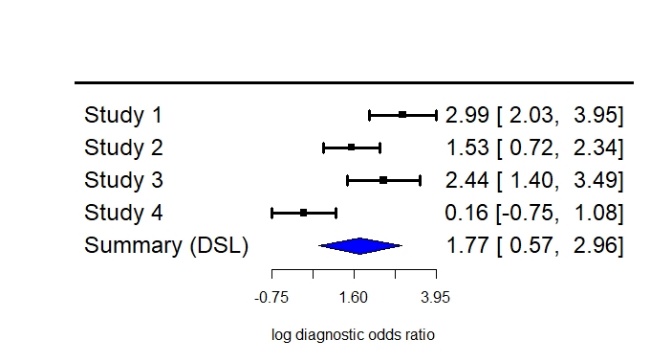Dentin detection level | 1/3 dentin detection level |
| Laser Fluorescence Pen 2190 | **Lussi and Hellwig et al. (2006)**  **Rodrigues and Hug et al. (2011)**  **Summary** | **Lussi and Hellwig et al. (2006)**  **Rodrigues et al. (2008)**  **Rodrigues and Hug et al. (2011)**  **Mortensen et al. (2014)**  **Summary** | No Data Available |
| FOTI | No Data Available | 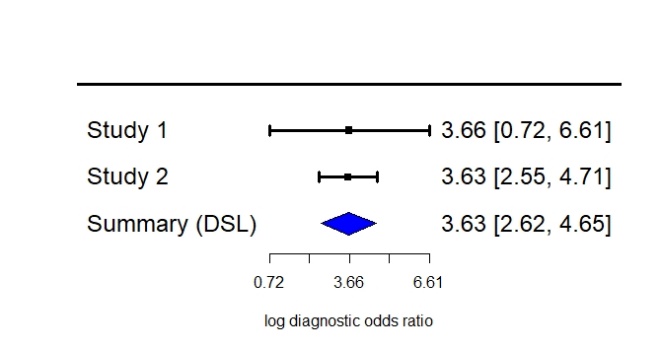  **Cortes et al. (2000)**  **Cortes et al. (2003)**  **Summary** | No Data Available |

## **Table S17 Forest plots (DOR) for different caries diagnostic methods- in vivo validation studies on occlusal surfaces**

| **Forest Plots** | **In vivo** | | |
| --- | --- | --- | --- |
|  | Caries detection level | Dentin detection level | 1/3 dentin detection level |
| **Visual examination** | 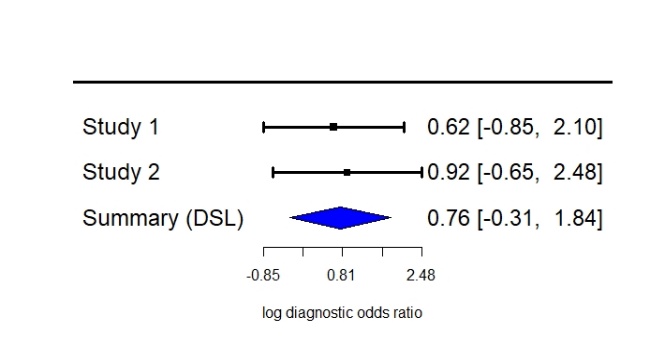  **Cotta et al. (2015)**  **Castilho et al. (2016)**  **SummaryT** | No Data Available | 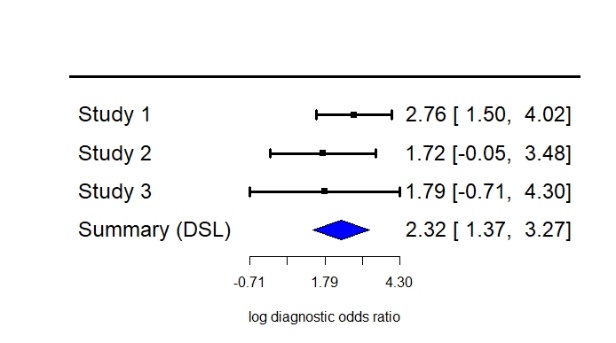  **Reis et al. (2006)**  **Cotta et al. (2015)**  **Castilho et al. (2016)**  **Summary** |
| **Conventional bitewing radiography**  **(D -speed)** | No Data Available | 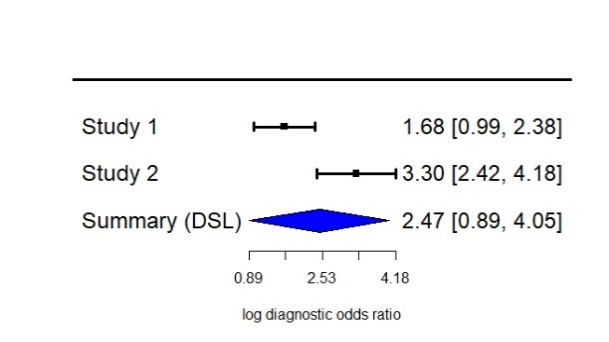  **Lussi et al. (1995)**  **Akarsu et al. (2006)**  **Summary** |  |
| **Conventional bitewing radiography**  **(E -speed)** | No Data Available | 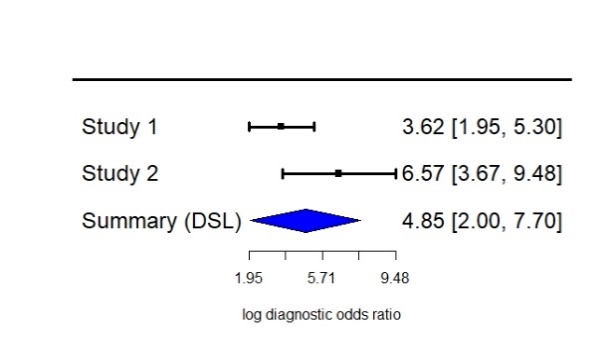  **Heinrich-Weltzien et al. (2002)**  **Bahrololoomi et al. (2015)**  **Summary** | No Data Available |
| **Conventional bitewing radiography**  **(F -speed)** | No Data Available | No Data Available | No Data Available |

| Forest Plots | In vivo | | |
| --- | --- | --- | --- |
|  | Caries detection level | Dentin detection level | 1/3 dentin detection level |
| Digital bitewing radiography | No Data Available | No Data Available | No Data Available |
| Laser Fluorescence 2095 | No Data Available | 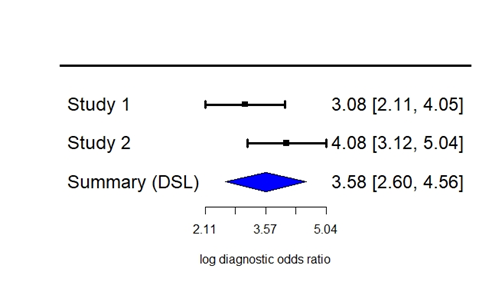  **Heinrich-Weltzien et al. (2002)**  **Akarsu et al. (2006)**  **Summary** | No Data Available |
| Laser Fluorescence Pen 2190 | No Data Available | No Data Available | No Data Available |
| FOTI | No Data Available | No Data Available | No Data Available |

# References

Abalos C, Herrera M, Jimenez-Planas A, Llamas R. (2009) Performance of laser fluorescence for detection of occlusal Dentinal caries lesions in permanent molars: an in vivo study with total validation of the sample. Caries Res 43(2):137-41

Abalos C, Mendoza A, Jimenez-Planas A, Guerrero E, Chaparro A, Garcia-Godoy F. (2012) Performance of laser fluorescence for the detection of enamel caries in non-cavitated occlusal surfaces: clinical study with total validation of the sample. Am J Dent 25(1):44-8

Abrams SH et al. (2017) Correlation with Caries Lesion Depth of the Canary System, Diagnodent and Icdas Ii. Open Dent J 11: 679-89

Abreu Junior M, Tyndall DA, Platin E, Ludlow JB, Phillips C. (1999) Two- and three-dimensional imaging modalities for the detection of caries. A comparison between film, digital radiography and tuned aperture computed tomography (TACT). Dentomaxillofacial Rad 28(3):152-7

Abreu M Jr, Tyndall DA, Ludlow JB (1999) Detection of Caries with Conventional Digital Imaging and Tuned Aperture Computed Tomography Using Crt Monitor and Laptop Displays. Oral Surg Oral Med Oral Pathol Oral Radiol Endod 88(2): 234-8

Achilleos EE, Rahiotis C, Kakaboura A, Vougiouklakis G. (2013) Evaluation of a new fluorescence-based device in the detection of incipient occlusal caries lesions. Lasers Med Sci 28(1):193-201

Akarsu S, Koprulu H. (2006) In vivo comparison of the efficacy of DIAGNOdent by visual inspection and radiographic diagnostic techniques in the diagnosis of occlusal caries. J Clin Dent 17(3):53-8

Aktan AM, Cebe MA, Ciftci ME, Sirin Karaarslan E. (2012) A novel LED-based device for occlusal caries detection. Lasers Med Sci 27(6):1157-63

Alammari M, Smith P, De Jong EDJ, Higham S. (2013) Quantitative light-induced fluorescence (QLF): a tool for early occlusal dental caries detection and supporting decision making in vivo. J Dent 41(2):127-32

Alkurt, MT, Peker I, Arisu HD, Bala O, Altunkaynak B. (2008). In vivo comparison of laser fluorescence measurements with conventional methods for occlusal caries detection. Lasers Med Sci 23(3), 307-312

Alomari QD, Qudeimat MA, Ghayyath AA (2015) Imaging of Occlusal Dentine Caries: A Comparison among Conventional Radiographs, Digital Radiographs, and Cone-Beam Computed Tomography Images. Oral Radiology 31(2): 73-80

Alomari QD, Qudeimat MA, Khalaf ME, Al-Tarakemah Y. (2015) The Effect of Combining Radiographs and DIAGNOdent With Visual Examination on Detection and Treatment Decisions of Noncavitated Occluso-Dentinal Caries. Oper Dent 40(3):313-21

Alwas-Danowska HM, Plasschaert AJ, Suliborski S. (2002) Verdonschot EH. Reliability and validity issues of laser fluorescence measurements in occlusal caries diagnosis. J Dent 30(4):129-34

Ando M. (2000) Comparative studies of several methods for the early detection of fissure lesions. Early detection of dental caries 279-99

Angnes G, Angnes V, Grande RH, Battistella M, Loguercio AD, Reis A. (2005) Occlusal caries diagnosis in permanent teeth: an in vitro study. Braz Oral Res 19(4):243-8

Angnes V, Angnes G, Batisttella M, Grande RH, Loguercio AD, Reis A. (2005) Clinical effectiveness of laser fluorescence, visual inspection and radiography in the detection of occlusal caries. Caries Res 39(6):490-5

Anttonen V, Seppa L, Hausen H. (2003) Clinical study of the use of the laser fluorescence device DIAGNOdent for detection of occlusal caries in children. Caries Res 37(1):17-23

Arslan U, Karaagaoglu E, Ozkan G, Kanli A. (2014) Evaluation of diagnostic tests using information theory for multi-class diagnostic problems and its application for the detection of occlusal caries lesions. Balkan Med J 31(3):214-8

Ashley PF, Blinkhorn AS, Davies RM. (1999) Occlusal caries diagnosis: an in vitro histological validation of the Electronic Caries Monitor (ECM) and other methods. J Dent 26(2):83-8

Astvaldsdottir A, Holbrook WP, Tranaeus S. (2004) Consistency of DIAGNOdent instruments for clinical assessment of fissure caries. Acta odontologica Scandinavica 62(4):193-8.

Bahrololoomi Z, Ezoddini F, Halvani N. (2015) Comparison of Radiography, Laser Fluorescence and Visual Examination for Diagnosing Incipient Occlusal Caries of Permanent First Molars. J Dent(Tehran, Iran) 12(5):324-32

Bamzahim M, Shi XQ, Angmar-Mansson B. (2002) Occlusal caries detection and quantification by DIAGNOdent and Electronic Caries Monitor: in vitro comparison. Acta Odont Scandi 60(6):360-4

Barberia E, Maroto M, Arenas M, Silva CC. (2008) A clinical study of caries diagnosis with a laser fluorescence system. J Am Dent Assoc 139(5):572-9

Baseren NM, Gokalp S. (2003) Validity of a laser fluorescence system (DIAGNOdent) for detection of occlusal caries in third molars: an in vitro study. J Oral Rehab 30(12):1190-4

Bottenberg P et al. (2016)Comparison of Occlusal Caries Detection Using the Icdas Criteria on Extracted Teeth or Their Photographs. BMC Oral Health 16(1): 93

Boye U, Walsh T, Pretty IA, Tickle M. (2012) Comparison of photographic and visual assessment of occlusal caries with histology as the reference standard. BMC oral health. 12:10

Boynton JR, et al.(2009) In Vivo Evaluation of Diagnodent for the Occlusal Dental Caries. Oper Den 34(2): 136-41

Bozdemir E, Karaarslan ES, Ozsevik AS, Ata Cebe M, Aktan AM. (2013) In vivo performance of two devices for occlusal caries detection. Photomed Laser Surg 31(7):322-7

Braga MM et al. (2007) Effect of Cut-Off Points on Performance of Laser Fluorescence for Detecting Occlusal Caries. J Clin Pediatr Dent 32(1):33-6

Braun A, Guiraud LM, Frankenberger R. (2017) Histological validation of ICDAS II and radiological assessment of occlusal carious lesions in permanent teeth. J Odont 105(1):46-53

Bucher K, Galler M, Seitz M, Hickel R, Kunzelmann KH, Kuhnisch J. (2015) Occlusal caries extension in relation to visual and radiographic diagnostic criteria: results from a microcomputed tomography study. Oper Dent 40(3):255-62

Burin C, Burin C, Loguercio AD, Grande RH, Reis A. (2005) Occlusal caries detection: a comparison of a laser fluorescence system and conventional methods. Ped Dent 27(4):307-12

Bussaneli DG, Restrepo M, Boldieri T, Pretel H, Mancini MW, Santos-Pinto L. (2015) Assessment of a new infrared laser transillumination technology (808 nm) for the detection of occlusal caries-an in vitro study. Lasers Med Sci 30(7):1873-9

Castilho LS, Cotta FV, Bueno AC, Moreira AN, Ferreira EF, Magalhaes CS. (2016) Validation of DIAGNOdent laser fluorescence and the International Caries Detection and Assessment System (ICDAS) in diagnosis of occlusal caries in permanent teeth: an in vivo study. European J Oral Sci 124(2):188-94

Chong MJ et al. (2003) Visual-Tactile Examination Compared with Conventional Radiography, Digital Radiography, and Diagnodent in the Diagnosis of Occlusal Occult Caries in Extracted Premolars. Pediatr Dent 25(4): 341-9

Chu CH, Lo EC, You DS. (2010) Clinical Diagnosis of Fissure Caries with Conventional and Laser-Induced Fluorescence Techniques. Lasers Med Sci 25(3): 355-62

Chu CH, You DS, Lo EC. (2009): Clinical Visual Detection of Fissure Caries with and without Diagnodent." Lasers Med Sci 24 (3): 473

Cortes DF, Ekstrand KR, Elias-Boneta AR, Ellwood RP. (2000) An in vitro comparison of the ability of fibre-optic transillumination, visual inspection and radiographs to detect occlusal caries and evaluate lesion depth. Caries Res 34(6):443-7

Cortes DF, Ellwood RP, Ekstrand KR. (2003) An in vitro comparison of a combined FOTI/visual examination of occlusal caries with other caries diagnostic methods and the effect of stain on their diagnostic performance. Caries Res 37(1):8-16

Costa AM, Bezzerra AC, Fuks AB. (2007) Assessment of the accuracy of visual examination, bite-wing radiographs and DIAGNOdent on the diagnosis of occlusal caries. EAPD 8(2):118-22

Costa AM, Paula LM, Bezerra AC. (2008) Use of Diagnodent for diagnosis of non-cavitated occlusal dentin caries. J Appl Oral Sci: revista FOB 16(1):18-23

Costa AM, Yamaguti PM, De Paula LM, Bezerra AC. (2002) In vitro study of laser diode 655 nm diagnosis of occlusal caries. ASDC J Dent Child 69(3):249-53, 33

Cotta F, de Castilho LS, Moreira AN, Paiva SM, Ferreira EF, Ferreira LC. (2015) Lesion Activity Assessment (LAA) in Conjunction With International Caries Detection and Assessment System (ICDAS) for Occlusal Caries Diagnosis in Permanent Teeth. Oper Dent 40(5):E189-96.

De Paula AB, Campos JA, Diniz MB, Hebling J, Rodrigues JA. (2011) In situ and in vitro comparison of laser fluorescence with visual inspection in detecting occlusal caries lesions. Lasers Med Sci 26(1):1-5

De Souza AL et al. (2014) The Caries Assessment Spectrum and Treatment (Cast) Instrument: Its Reproducibility in Clinical Studies. Int Dent J 64(4): 187-94

Deery C et al. (2006) Effect of Placing a Clear Sealant on the Validity and Reproducibility of Occlusal Caries Detection by a Laser Fluorescence Device: An in Vitro Study. Caries Res 40(3): 186-93

Deery C. (1995) The Effect of Placing a Clear Pit and Fissure Sealant on the Validity and Reproducibility of Occlusal Caries Diagnosis. Caries Res 29(5): 377-81

Diniz MB et al. (2010) Influence of Examiner's Clinical Experience on the Reproducibility and Accuracy of Radiographic Examination in Detecting Occlusal Caries. Clin Oral Investig 14(5): 515-23

Diniz MB, Boldieri T, Rodrigues JA, Santos-Pinto L, Lussi A, Cordeiro RC. (2012) The performance of conventional and fluorescence-based methods for occlusal caries detection: an in vivo study with histologic validation. J Am Dent Assoc 143(4):339-50

Diniz MB, Lima LM, Eckert G, Zandona AG, Cordeiro RC, Pinto LS.(2011) In vitro evaluation of ICDAS and radiographic examination of occlusal surfaces and their association with treatment decisions. Oper Dent 36(2):133-42

Diniz MB, Rodrigues JA, de Paula AB, Cordeiro Rde C. (2009) In vivo evaluation of laser fluorescence performance using different cut-off limits for occlusal caries detection. Lasers Med Sci 24(3):295-300

Diniz MB, Rodrigues JA, Hug I, Cordeiro Rde C, Lussi A. (2009) Reproducibility and accuracy of the ICDAS-II for occlusal caries detection. Community Dent Oral Epidemiol 37(5):399-404

Downer MC, Kay EJ (1996). Restorative treatment decisions from bitewing radiographs--performance of dental epidemiologists and general dental practitioners. Community Dent Oral Epidemiol; 24(2):101-5

Duruturk L et al. (2011) Clinical Evaluation of Diagnodent in Detection of Occlusal Caries in Newly Erupted Noncavitated First Permanent Molars in Caries-Active Children. Oper Dent 36(4): 348-55

Ekstrand KR et al. (1995) Relationship between External and Histologic Features of Progressive Stages of Caries in the Occlusal Fossa. Caries Res 29(4): 243-50

Ekstrand KR et al. (2007) Accuracy and Reproducibility of Conventional Radiographic Assessment and Subtraction Radiography in Detecting Demineralization in Occlusal Surfaces. Caries Res 41(2):121-28

Ekstrand KR, Martignon S, Ricketts DJ, Qvist V. (2007) Detection and activity assessment of primary coronal caries lesions: a methodologic study. Oper Dent 32(3):225-35

Ekstrand KR, Ricketts DN, Kidd EA. (1997) Reproducibility and accuracy of three methods for assessment of demineralization depth of the occlusal surface: an in vitro examination. Caries Res 31(3):224-31

El-Housseiny AA, Jamjoum H. (2001) Evaluation of visual, explorer, and a laser device for detection of early occlusal caries. J Clin Ped Dent 26(1):41-8

Ertas ET, Küçükyılmaz E, Ertaş H, Savaş S, Atıcı MY. (2014). A comparative study of different radiographic methods for detecting occlusal caries lesions. Caries Res 48(6), 566-574

Erten H, Uctasli MB, Akarslan ZZ, Uzun O, Baspinar E. (2005) The assessment of unaided visual examination, intraoral camera and operating microscope for the detection of occlusal caries lesions. Oper Dent 30(2):190-4

Erten H, Uctasli MB, Akarslan ZZ, Uzun O, Semiz M. (2006) Restorative treatment decision making with unaided visual examination, intraoral camera and operating microscope. Oper Dent 31(1):55-9

Espelid I, Tveit AB, Fjelltveit A. (1994) Variations among dentists in radiographic detection of occlusal caries. Caries Res 28(3):169-75.

Espelid I, Tveit AB. (2001) A Comparison of Radiographic Occlusal and Approximal Caries Diagnoses Made by 240 Dentists. Acta Odontol Scand 59(5):285-9

Ferreira Zandona AG, Analoui M, Beiswanger BB, Isaacs RL, Kafrawy AH, Eckert GJ. (1998) An in vitro comparison between laser fluorescence and visual examination for detection of demineralization in occlusal pits and fissures. Caries Res 32(3):210-8

Forgie AH, Pine CM, Pitts NB. (2003) The assessment of an intra-oral video camera as an aid to occlusal caries detection. Int Dent J 53(1):3-6

Francescut P, Lussi A. (2003) Correlation between fissure discoloration, Diagnodent measurements, and caries depth: an in vitro study. Ped Dent 25(6):559-64

Fung L, Smales R, Ngo H, Moun G. (2004) Diagnostic comparison of three groups of examiners using visual and laser fluorescence methods to detect occlusal caries in vitro. Australian dental journal 49(2):67-71; quiz 101

Galcera Civera V et al. (2007) Clinical and Radiographic Diagnosis of Approximal and Occlusal Dental Caries in a Low Risk Population. Med Oral Patol Oral Cir Bucal 12(3): E252-7

Ghaname ES et al. (2010) Correlation between Laser Fluorescence Readings and Volume of Tooth Preparation in Incipient Occlusal Caries in Vitro. J Esthet Restor Dent 22.1 (2010): 31-39

Gomez J, Zakian C, Salsone S, Pinto SC, Taylor A, Pretty IA, et al. (2013) In vitro performance of different methods in detecting occlusal caries lesions. J Dent 41(2):180-6

Gottlieb R et al. (2014) An Automated Dental Caries Detection and Scoring System for Optical Images of Tooth Occlusal Surface. Conference proceedings:. Annual International Conference of the IEEE Engineering in Medicine and Biology Society. IEEE Engineering in Medicine and Biology Society. Annual Conference. 2014 1925-28

Gray GB, Paterson RC. (1997) Fissure caries diagnosis and resulting treatment decisions by clinical community dental officers and general dental practitioners. The European journal of prosthodontics and restorative dentistry 5(1):23-9

Grossman ES, Cleaton-Jones PE, Cortes DF, Daya NP, Parak RB, Fatti LP. (2002) Accurate diagnosis of occlusal carious lesions--a stereo microscope evaluation of clinical diagnosis. SADJ : J South Afri Dent Ass 57(6):215-20

Haak R, Wicht MJ. (2004) Caries Detection and Quantification with Diagnodent: Prospects for Occlusal and Root Caries. Int. J. Comput. Dent. 7(4): 347-58

Heinrich-Weltzien R et al. (2003) Comparison of Different Diagnodent Cut-Off Limits for in Vivo Detection of Occlusal Caries. Oper Dent 28(6): 672-80

Heinrich-Weltzien R, Weerheijm KL, Kuhnisch J, Oehme T, Stosser L. (2002) Clinical evaluation of visual, radiographic, and laser fluorescence methods for detection of occlusal caries. ASDC J Dent Child 69(2):127-32, 3

Hintze H, Christoffersen L, Wenzel A. (1996) In vitro comparison of Kodak Ultra-speed, Ektaspeed, and Ektaspeed Plus, and Agfa M2 Comfort dental x-ray films for the detection of caries. Oral surgery, oral medicine, oral pathology, oral radiology, and endodontics 81(2):240-4

Hintze H, Wenze A. (1996) Clinical and laboratory radiographic caries diagnosis. A study of the same teeth. Dento maxillo facial radiology 25(3):115-8

Hintze H, Wenzel A, Frydenberg M. (2002) Accuracy of Caries Detection with Four Storage Phosphor Systems and E-Speed Radiographs. Dentomaxillofac Radiol 31(3): 170-5

Hintze H, Wenzel A, Larsen MJ. (1995) Stereomicroscopy, Film Radiography, Microradiography and Naked-Eye Inspection of Tooth Sections as Validation for Occlusal Caries Diagnosis. Caries Res 29(5): 359-63

Hintze H, Wenzel A. (2002) Influence of the validation method on diagnostic accuracy for caries. A comparison of six digital and two conventional radiographic systems. Dentomaxillofacial Rad 31(1):44-9

Huth KC, Neuhaus KW, Gygax M, Bucher K, Crispin A, Paschos E, et al. (2008) Clinical performance of a new laser fluorescence device for detection of occlusal caries lesions in permanent molars. J Dent 36(12):1033-40

Huysmans, MC et al. (1998) Surface-Specific Electrical Occlusal Caries Diagnosis: Reproducibility, Correlation with Histological Lesion Depth, and Tooth Type Dependence. Caries Res 32(5): 330-6

Ie YL, Verdonschot EH, Schaeken MJ, van't Hof MA. (1995) Electrical conductance of fissure enamel in recently erupted molar teeth as related to caries status. Caries Res 29(2):94-9

Iranzo-Cortes JE, Terzic S, Montiel-Company JM, Almerich-Silla JM. (2017) Diagnostic validity of ICDAS and DIAGNOdent combined: an in vitro study in pre-cavitated lesions. Lasers Med Sci 32(3):543-8

Jablonski-Momeni A et al. (2013) Performance of a New Fluorescence Camera for Detection of Occlusal Caries in Vitro. Lasers Med Sci 28(1): 101-9

Jablonski-Momeni A, Heinzel-Gutenbrunner M, Klein SM. In vivo performance of the VistaProof fluorescence-based camera for detection of occlusal lesions. (2014) Clin Oral Inv 18(7):1757-62

Jablonski-Momeni A, Schipper HM, Rosen SM, Heinzel-Gutenbrunner M, Roggendorf MJ, Stoll R. (2011) Performance of a fluorescence camera for detection of occlusal caries in vitro. J Odont 99(1):55-61

Jablonski-Momeni A, Stachniss V, Ricketts DN, Heinzel-Gutenbrunner M, Pieper K. (2008) Reproducibility and accuracy of the ICDAS-II for detection of occlusal caries in vitro. Caries Res 42(2):79-87

Jablonski-Momeni A, Stucke J, Steinberg T, Heinzel-Gutenbrunner M. (2012) Use of ICDAS-II, Fluorescence-Based Methods, and Radiography in Detection and Treatment Decision of Occlusal Caries Lesions: An In Vitro Study. Int J Dent 371595

Jallad M, Zero D, Eckert G, Ferreira Zandona A. (2015) In vitro Detection of Occlusal Caries on Permanent Teeth by a Visual, Light-Induced Fluorescence and Photothermal Radiometry and Modulated Luminescence Methods. Caries Res 49(5):523-30

Kamburoglu K, Senel B, Yuksel SP, Ozen T. .(2010) A comparison of the diagnostic accuracy of in vivo and in vitro photostimulable phosphor digital images in the detection of occlusal caries lesions Dentomaxillofac Radiol 39(1):17-22

Kay EJ, Watts A, Paterson RC, Blinkhorn AS. (1988) Preliminary investigation into the validity of dentists' decisions to restore occlusal surfaces of permanent teeth. Community Dent. Oral Epidemiol16(2):91-4

Ketley CE, Holt RD. (1993) Visual and radiographic diagnosis of occlusal caries in first permanent molars and in second primary molars. Br Dent J 174(10):364-70

Kordic A, Lussi A, Luder HU. (2003) Performance of visual inspection, electrical conductance and laser fluorescence in detecting occlusal caries in vitro. Schweizer Monatsschrift fur Zahnmedizin 113(8):852-9

Kouchaji C et al. (2012) Comparison between a Laser Fluorescence Device and Visual Examination in the Detection of Occlusal Caries in Children. Saudi Dent J 24(3-4): 169-74

Krause F, Jepsen S, Braun A. (2007) Comparison of two laser fluorescence devices for the detection of occlusal caries in vivo. Eur J Oral Sci 115(4):252-6

Krzyzostaniak J et al. (2014) Diagnostic Accuracy of Cone Beam Computed Tomography Compared with Intraoral Radiography for the Detection of Noncavitated Occlusal Carious Lesions. Caries Res 48(5): 461-6.

Krzyzostaniak J, Kulczyk T, Czarnecka B, Surdacka A. (2015) A comparative study of the diagnostic accuracy of cone beam computed tomography and intraoral radiographic modalities for the detection of noncavitated caries. Clin Oral Inv 19(3):667-72

Kühnisch J et al. (2007) In Vivo Detection of Non-Cavitated Caries Lesions on Occlusal Surfaces by Visual Inspection and Quantitative Light-Induced Fluorescence. Acta Odontol Scand 65(3): 183-8

Kuhnisch J, Bucher K, Henschel V, Hickel R. (2007) Reproducibility of DIAGNOdent 2095 and DIAGNOdent Pen measurements: results from an in vitro study on occlusal sites. Eur J Oral Sc 115(3):206-11

Kuhnisch J, Bucher K, Hickel R. (2007) The intra/inter-examiner reproducibility of the new DIAGNOdent Pen on occlusal sites. J Dent 35(6):509-12

Kuhnisch J, Ifland S, Tranaeus S, Angmar-Mansson B, Hickel R, Stosser L. (2006) Establishing quantitative light-induced fluorescence cut-offs for the detection of occlusal dentine lesions. Eur J Oral Sci 114(6):483-8

Kuhnisch J, Ifland S, Tranaeus S, Heinrich-Weltzien R. (2009) Comparison of visual inspection and different radiographic methods for dentin caries detection on occlusal surfaces. Dentomaxillofacial Rad 38(7):452-7

Kuhnisch J, Ziehe A, Brandstadt A, Heinrich-Weltzien R. (2004) An in vitro study of the reliability of DIAGNOdent measurements. J Oral Rehabil 31(9):895-9

Lara-Capi C et al. (2017) Digital Transillumination in Caries Detection Versus Radiographic and Clinical Methods: An in-Vivo Study. DMFR 46(4): 20160417

Lazarchik DA, Firestone AR, Heaven TJ, Filler SJ, Lussi A. (1995) Radiographic evaluation of occlusal caries: effect of training and experience. Caries Res 29(5):355-8

Lizarelli RF, Bregagnolo JC, Lizarelli RZ, Palhares JM, Villa GE. (2004) A comparative in vitro study to diagnose decayed dental tissue using different methods. Photomed Laser Surg 22(3):205-10

Lupi-Pegurier L et al. (2009) Occlusal Caries Diagnosis in Permanent Teeth: An in Vitro Study. Lasers Med Sci 24 (3): 490

Lussi A, Firestone A, Schoenberg V, Hotz P, Stich H. (1995) In vivo diagnosis of fissure caries using a new electrical resistance monitor. Caries Res 29(2):81-7

Lussi A, Francescut P. (2003) Performance of conventional and new methods for the detection of occlusal caries in deciduous teeth. Caries Res 37(1):2-7

Lussi A, Hellwig E. Performance of a new laser fluorescence device for the detection of occlusal caries in vitro. J Dent 34(7):467-71

Lussi A, Imwinkelried S, Pitts N, Longbottom C, Reich E. (1999) Performance and reproducibility of a laser fluorescence system for detection of occlusal caries in vitro. Caries Res 33(4):261-6

Lussi A, Megert B, Longbottom C, Reich E, Francescut P. (2001) Clinical performance of a laser fluorescence device for detection of occlusal caries lesions. Eur J Oral Sci 109(1):14-9

Lussi A. (1993) Comparison of different methods for the diagnosis of fissure caries without cavitation. Caries Res 27(5):409-16

Lussi A. et al. (1996) Impact of Including or Excluding Cavitated Lesions When Evaluating Methods for the Diagnosis of Occlusal Caries. Caries Res 30(6): 389-93

Lussi A. Validity of diagnostic and treatment decisions of fissure caries. Caries Res. 1991;25(4):296-303.

Machiulskiene V, Nyvad B, Baelum V. (1999) A Comparison of Clinical and Radiographic Caries Diagnoses in Posterior Teeth of 12-Year-Old Lithuanian Children. Caries Res 33(5)340-8

Manton DJ, Messer LB. (2007) The Effect of Pit and Fissure Sealants on the Detection of Occlusal Caries in Vitro. Eur Arch Paediatr Dent 8.1 (2007): 43-8

Markowitz K et al. (2015) In Vitro Study of the Diagnostic Performance of the Spectra Caries Detection Aid. J Clin Dent 26(1): 17-22

Maroto M, Arenas M, Cardoso Silva C. (2008) A Clinical Study of Caries Diagnosis with a Laser Fluorescence System. J Am Dent Assoc 139(5): 572-79

Marthaler TM (1966) A standardized system of recording dental conditions. Helv Odontol Acta; 10(1):1-18

Matos AB et al. (2012): In Vitro Evaluation of Laser Fluorescence Performance for Detection of Artificial Caries-Affected Dentin in Permanent Molars. Med Oral Patol Oral Cir Bucal 17:S176

Melo M, Pascual A, Camps I, Del Campo A, Ata-Ali J. (2017) Caries diagnosis using light fluorescence devices in comparison with traditional visual and tactile evaluation: a prospective study in 152 patients. J Odont 105(3):283-90.

Mendoza A et al. (2012) Performance of Laser Fluorescence for the Detection of Enamel Caries in Non-Cavitated Occlusal Surfaces: Clinical Study with Total Validation of the Sample. Am J Dent 25(1): 44-48

Mestriner SF, Vinha D, Mestriner Junior W. (2005) Comparison of different methods for the occlusal dentine caries diagnosis. J App Oral Sci: revista FOB 13(1):28-34

Mitropoulos P, Rahiotis C, Kakaboura A, Vougiouklakis G. (2012) The impact of magnification on occlusal caries diagnosis with implementation of the ICDAS II criteria. Caries Res 46(1):82-6

Mortensen D, Dannemand K, Twetman S, Keller MK. (2014) Detection of non-cavitated occlusal caries with impedance spectroscopy and laser fluorescence: an in vitro study. Open Dent j 8:28-32

Muller-Bolla M et al. (2017) Performance of a Recent Light Fluorescence Device for Detection of Occlusal Carious Lesions in Children and Adolescents. Eur Arch Paediatr Dent 18(3): 187-95

Neuhaus KW, Jost F, Perrin P, Lussi A. (2015) Impact of different magnification levels on visual caries detection with ICDAS. J Dent 43(12):1559-64

Nytun RB, Raadal M, Espelid I.(1992) Diagnosis of dentin involvement in occlusal caries based on visual and radiographic examination of the teeth. Scand J Dent Res100(3):144-8.

Nyvad B, Machiulskiene V, Bælum V. (1999) Reliability of a new caries diagnostic system differentiating between active and inactive caries lesions. Caries Res 33(4):252-60

Oancea R, Podariu AC, Vasile L, Sava-Rosianu R, Folescu R. (2013) In vitro evaluation of laser fluorescence devices for caries detection through stereomicroscopic imaging. Romanian journal of morphology and embryology 54(2):333-41

Olmez A, Tuna D, Oznurhan F. (2006)Clinical Evaluation of Diagnodent in Detection of Occlusal Caries in Children. J Clin Pediatr Dent 30(4): 287-91

Ouellet A, Hondrum SO, Pietz DM. Detection of occlusal carious lesions. Gen Dent 50(4):346-50

Ozkan G, Kanli A, Baseren NM, Arslan U, Tatar I. (2015) Validation of micro-computed tomography for occlusal caries detection: an in vitro study. Brazilian Oral Res 29(1): S1806-83242015000100309

Ozturk E, Sinanoglu A. (2015) Histological validation of cone-beam computed tomography versus laser fluorescence and conventional diagnostic methods for occlusal caries detection. Photomed Laser Surg 33(2):61-8

Patel SA, Shepard WD, Barros JA, Streckfus CF, Quock RL. (2014) In vitro evaluation of Midwest Caries ID: a novel light-emitting diode for caries detection. Oper Dent 39(6):644-51

Pereira AC, Eggertsson H, Martinez-Mier EA, Mialhe FL, Eckert GJ, Zero DT. (2009) Validity of caries detection on occlusal surfaces and treatment decisions based on results from multiple caries-detection methods. Eur J Oral Sci 117(1):51-7

Pereira AC, Verdonschot EH, Huysmans MC. (2001) Caries detection methods: can they aid decision making for invasive sealant treatment? Caries Res 35(2):83-9

Peycheva K, Boteva E. (2016) A Comparison of Different Methods for Fissure Caries Detection. Acta Med Bulg 43(1): 30-38

Pourhashemi SJ, Baniameri Z, Hasanzadeh A. (2013). Evaluation of DIAGNOdent Values Before and After the Application of Opaque Fissure Sealant to Permanent Teeth. J Islam Dent Assoc Iran 25(4), 219-222

Pourhashemi SJ, Jafari A, Motahhari P, Panjnoosh M, Kharrazi Fard MJ, Sanati I, et al. (2009) An in-vitro comparison of visual inspection, bite-wing radiography, and laser fluorescence methods for the diagnosis of occlusal caries. J Indian Soc Pedod Prev Dent 27(2):90-3

Qudeimat MA, Alomari QD, Altarakemah Y, Alshawaf N, Honkala EJ. (2016) Variables affecting the inter- and intra-examiner reliability of ICDAS for occlusal caries diagnosis in permanent molars. J Pub Health Dent 76(1):9-16

Ramezani L, Salemi F, Shokri A, Sichani HF, Mirzayi M, Bagheri M. (2016). A comparative study of the diagnostic accuracy of cone beam computed tomography and phosphor storage plate for detection of noncavitated occlusal caries Dent Med Probl 53(2), 186-192

Rando-Meirelles MP, de Sousa Mda L. (2011) Using laser fluorescence (DIAGNOdent) in surveys for the detection of noncavitated occlusal dentine caries. Commun Dent Health 28(1):17-21

Rechmann P, Charland D, Rechmann BM, Featherstone JD(2012). Performance of laser fluorescence devices and visual examination for the detection of occlusal caries in permanent molars. J Biomed Optics 17(3):036006

Reis A, Mendes FM, Angnes V, Angnes G, Grande RH, Loguercio AD. (2006) Performance of methods of occlusal caries detection in permanent teeth under clinical and laboratory conditions. J Dent 34(2):89-96

Reis A, Zach VL, Jr., de Lima AC, de Lima Navarro MF, Grande RH. (2004) Occlusal caries detection: a comparison of DIAGNOdent and two conventional diagnostic methods. J Clin Dent 15(3):76-82

Ricketts DN, Kidd EA, Smith BG, Wilson RF. (1995) Clinical and radiographic diagnosis of occlusal caries: a study in vitro. Journal of oral rehabilitation 22(1):15-20

Ricketts DN, Kidd EA, Wilson RF. (1995) A re-evaluation of electrical resistance measurements for the diagnosis of occlusal caries. Br Dent J 178(1):11-7

Rocha AS, Almeida SM, Boscolo FN, Haiter Neto F. (2005) Interexaminer agreement in caries radiographic diagnosis by conventional and digital radiographs. J App Oral Sci: revista FOB 13(4):329-33

Rodrigues JA, Diniz MB, Josgrilberg EB, Cordeiro RC. (2009) In vitro comparison of laser fluorescence performance with visual examination for detection of occlusal caries in permanent and primary molars. Lasers Med Sci 24(4):501-6

Rodrigues JA, Hug I, Diniz MB, Lussi A. (2008) Performance of fluorescence methods, radiographic examination and ICDAS II on occlusal surfaces in vitro. Caries Res 42(4):297-304

Rodrigues JA, Hug I, Neuhaus KW, Lussi A. (2011) Light-emitting diode and laser fluorescence-based devices in detecting occlusal caries. J Biomed Optics 16(10):107003

Rodrigues JA, Neuhaus KW, Diniz MB, Hug I, Stich H, Karlsson L, Lussi A. (2012). Comparison among gold standard techniques used for the validation of methods for occlusal caries detection. Microsc Res Tech (5), 605-608

Rodrigues Jde A, Hug I, Lussi A. (2009) The influence of PVC wrapping on the performance of two laser fluorescence devices on occlusal surfaces in vitro. Photomed Laser Surg 27(3):435-9

Seremidi K, Lagouvardos P, Kavvadia K. (2012) Comparative in vitro validation of VistaProof and DIAGNOdent pen for occlusal caries detection in permanent teeth. Oper Dent 37(3):234-45

Sheehy EC, Brailsford SR, Kidd EA, Beighton D, Zoitopoulos L. (2001) Comparison between visual examination and a laser fluorescence system for in vivo diagnosis of occlusal caries. Caries Res 35(6):421-6

Shi XQ, Welander U, Angmar-Mansson B. (2000) Occlusal caries detection with KaVo DIAGNOdent and radiography: an in vitro comparison. Caries Res 34(2):151-8

Shinohara T, Takase Y, Amagai T, Haruyama C, Igarashi A, Kukidome N, et al. (2006) Criteria for a diagnosis of caries through the DIAGNOdent. Photomed Laser Surg 24(1):50-8

Sinanoglu A, Ozturk E, Ozel E. (2014) Diagnosis of occlusal caries using laser fluorescence versus conventional methods in permanent posterior teeth: a clinical study. Photomed Laser Surg 32(3):130-7

Sisodia N, Manjunath MK. (2014) Impact of low level magnification on incipient occlusal caries diagnosis and treatment decision making. Journal of clinical and diagnostic research : JCDR 8(8):Zc32-5

Souza-Zaroni WC, Ciccone JC, Souza-Gabriel AE, Ramos RP, Corona SA, Palma-Dibb RG. (2006) Validity and reproducibility of different combinations of methods for occlusal caries detection: an in vitro comparison. Caries Res 40(3):194-201

Sridhar N, Tandon S, Rao N. (2009) A comparative evaluation of DIAGNOdent with visual and radiography for detection of occlusal caries: an in vitro study. Indian J Dent Res 20(3):326-31

Theocharopoulou A, Lagerweij MD. (2015). Use of the ICDAS system and two fluorescence-based intraoral devices for examination of occlusal surfaces. Eur J Paediatr Dent 16(1), 51-55

Thomas MF, Ricketts DN, Wilson RF. (2001) Occlusal caries diagnosis in molar teeth from bitewing and panoramic radiographs. Primary dental care : journal of the Faculty of General Dental Practitioners (UK) 8(2):63-9

Tonioli MB, Bouschlicher MR, Hillis SL. (2002) Laser fluorescence detection of occlusal caries. Amer Dent J 15(4):268-73

Townsend D. (2001) Detection of dentine caries using the oblique lateral radiograph. Int J Paed Dent 10(2):145-9

Tranaeus S, Lindgren LE, Karlsson L, Angmar-Mansson B. (2004) In vivo validity and reliability of IR fluorescence measurements for caries detection and quantification. Swed Dent J 28(4):173-82

Tveit AB, Espelid I, Fjelltveit A. Clinical diagnosis of occlusal dentin caries. Caries Res. 1994;28(5):368-72

Valera FB, Pessan JP, Valera RC, Mondelli J, Percinoto C. (2008) Comparison of visual inspection, radiographic examination, laser fluorescence and their combinations on treatment decisions for occlusal surfaces. Am J Dent 21(1):25-9

Van Hilsen Z, Jones RS. (2013) Comparing potential early caries assessment methods for teledentistry. BMC oral health 13:16

Verdonschot EH, Bronkhorst EM, Burgersdijk RC, Konig KG, Schaeken MJ, Truin GJ. (1992) Performance of some diagnostic systems in examinations for small occlusal carious lesions. Caries Res 26(1):59-64

Verdonschot EH, Wenzel A, Truin GJ, Konig KG. (1993) Performance of electrical resistance measurements adjunct to visual inspection in the early diagnosis of occlusal caries. J Dent 21(6):332-7

Wenzel A, Borg E, Hintze H, Grondahl HG. (1995) Accuracy of caries diagnosis in digital images from charge-coupled device and storage phosphor systems: an in vitro study. Dentomaxillofacial Rad 24(4):250-4

Wenzel A, Fejerskov O, Kidd E, Joyston-Bechal S, Groeneveld A. (1990) Depth of occlusal caries assessed clinically, by conventional film radiographs, and by digitized, processed radiographs. Caries Res 24(5):327-33

Wenzel A, Fejerskov O. Validity of diagnosis of questionable caries lesions in occlusal surfaces of extracted third molars.(1992) Caries Res 26(3):188-94.

Wenzel A, Hintze H, Mikkelsen L, Mouyen F. Radiographic detection of occlusal caries in noncavitated teeth. A comparison of conventional film radiographs, digitized film radiographs, and RadioVisioGraphy. Oral surgery, oral medicine, and oral pathology. 1991;72(5):621-6.

Wenzel A, Verdonschot EH, Truin GJ, Konig KG. (1994) Impact of the validator and the validation method on the outcome of occlusal caries diagnosis. Caries Res 28(5):373-7

Wenzel A, Verdonschot EH, Truin GJ, Konig KG. Accuracy of visual inspection, fiber-optic transillumination, and various radiographic image modalities for the detection of occlusal caries in extracted non-cavitated teeth (1992) J Dent Res 71(12):1934-7.

Wolwacz VF, Chapper A, Busato AL, Barbosa AN. (2004) Correlation between visual and radiographic examinations of non-cavitated occlusal caries lesions -- an in vivo study. Braz Oral Res 18(2):145-9

Zafersoy-Akarslan Z, Erten H, Uzun Ö, Semiz M. (2009) Reproducibility and agreement of clinical diagnosis of occlusal caries using unaided visual examination and operating microscope. J Can Dent Assoc 75(6)

Zaidi I, Somani R, Jaidka S, Nishad M, Singh S, Tomar D. (2016) Evaluation of different diagnostic modalities for diagnosis of dental caries: an in vivo study. Int J Clin Pediatr Dent 9(4), 320

Zeitouny M, Feghali M, Nasr A, Abou-Samra P, Saleh N, Bourgeois D. (2014) SOPROLIFE system: an accurate diagnostic enhancer. Sci World J. 924741
